# Reliability and validity of three questionnaires measuring context-specific sedentary behaviour and associated correlates in adolescents, adults and older adults

**DOI:** 10.1186/s12966-015-0277-2

**Published:** 2015-09-17

**Authors:** Cedric Busschaert, Ilse De Bourdeaudhuij, Veerle Van Holle, Sebastien FM Chastin, Greet Cardon, Katrien De Cocker

**Affiliations:** Department Movement and Sport Sciences, Ghent University, Watersportlaan 2, B-9000 Ghent, Belgium; School of Health and Life Science, Institute of Applied Health Research, Glasgow Caledonian University, Cowcaddens Road, Glasgow, G4 0BA Scotland UK; Fund for Scientific Research Flanders (FWO), Egmontstraat 5, 1000 Brussels, Belgium

**Keywords:** Adolescents, Adults, Older adults, Questionnaire, Validity, Reliability, Sedentary behaviour

## Abstract

**Background:**

Reliable and valid measures of total sedentary time, context-specific sedentary behaviour (SB) and its potential correlates are useful for the development of future interventions. The purpose was to examine test-retest reliability and criterion validity of three newly developed questionnaires on total sedentary time, context-specific SB and its potential correlates in adolescents, adults and older adults.

**Methods:**

Reliability and validity was tested in six different samples of Flemish (Belgium) residents. For the reliability study, 20 adolescents, 22 adults and 20 older adults filled out the age-specific SB questionnaire twice. Test-retest reliability was analysed using Kappa coefficients, Intraclass Correlation Coefficients and/or percentage agreement, separately for the three age groups. For the validity study, data were retrieved from 62 adolescents, 33 adults and 33 older adults, with activPAL™ as criterion measure. Spearman correlations and Bland-Altman plots (or non-parametric approach) were used to analyse criterion validity, separately for the three age groups and for weekday, weekend day and average day.

**Results:**

The test-retest reliability for self-reported total sedentary time indicated following values: ICC = 0.37-0.67 in adolescents; ICC = 0.73-0.77 in adults; ICC = 0.68-0.80 in older adults. Item-specific reliability results (e.g. context-specific SB and its potential correlates) showed good-to-excellent reliability in 67.94 %, 68.90 % and 66.38 % of the items in adolescents, adults and older adults respectively. All items belonging to sedentary-related equipment and simultaneous SB showed good reliability. The sections of the questionnaire with lowest reliability were: context-specific SB (adolescents), potential correlates of computer use (adults) and potential correlates of motorized transport (older adults). Spearman correlations between self-reported total sedentary time and the activPAL™ were different for each age group: ρ = 0.02-0.42 (adolescents), ρ = 0.06-0.52 (adults), ρ = 0.38-0.50 (older adults). Participants over-reported total sedentary time (except for weekend day in older adults) compared to the activPAL™, for weekday, weekend day and average day respectively by +57.05 %, +46.29 %, +53.34 % in adolescents; +40.40 %, +19.15 %, +32.89 % in adults; +10.10 %, −6.24 %, +4.11 % in older adults.

**Conclusions:**

The questionnaires showed acceptable test-retest reliability and criterion validity. However, over-reporting of total SB was noticeable in adolescents and adults. Nevertheless, these questionnaires will be useful in getting context-specific information on SB.

**Electronic supplementary material:**

The online version of this article (doi:10.1186/s12966-015-0277-2) contains supplementary material, which is available to authorized users.

## Background

Sedentary behaviour (SB), defined as any waking activity characterized by an energy expenditure ≤ 1.5 metabolic equivalents (METs) performed in a sitting or reclining posture [1], is ubiquitous in daily life among all age groups. Independent of physical activity (PA), SB is associated with physical and mental health risks in adolescents [2, 3], adults [2], and older adults [4, 5]. Despite these health-related consequences, Belgian adolescents, adults and older adults have high levels of daily objectively measured total sedentary time (8.02 h.d^−1^, 8.27 h.d^−1^ and 9.67 h.d^−1^ respectively), which is similar in duration to their international peers (9.00 h.d^−1^, 9.64 h.d^−1^ and 9.00 h.d^−1^ respectively) [6–10].

SB is a complex behaviour as it is habitual in nature and occurs in multiple contexts [11], across four domains [12] (e.g. watching TV, sitting at work, during motorized transport or while doing household tasks) [13]. The contexts of SB are similar for adolescents, adults and older adults (e.g. reading and TV-viewing), but there are also important age-specific contexts, e.g. school-context for adolescents, work-context for adults and sitting while caring (grandchildren) for older adults. As a result, measuring context-specific SB is important. Measurements of context-specific SB will increase the effectiveness of future interventions by identifying high-risk SB contexts which can be targeted. Furthermore, gathering information on all relevant contexts of SB, based on a consensus taxonomy [11], will provide an estimation of total sitting time, which will be valuable in identifying highly sedentary subgroups in large-scale observational studies in which the use of objective measurement devices is still not practical due to cost or participant burden [7, 11, 13, 14].

In addition to context-specific SB, it is also important to determine the potential correlates of context-specific SB, for different age groups. Future interventions will only be effective if they target the reasons why people tend to sit more in specific contexts, but currently there is a dearth of information about these correlates [15]. To date, research on correlates of SB has focused on TV viewing, computer use and playing videogames or SB in general [16, 17]. Focussing solely on correlates of screen-related behaviour may limit interventions designed to evoke meaningful changes in total SB. On the other hand, gathering correlates of total SB is relevant [12], but is too broad to design more-effective interventions in the future. Questionnaires incorporating potential correlates of all specific contexts of SB are needed to develop more effective interventions. Previous studies on correlates concentrated on socio-demographic correlates together with one other type of variables, for example biological or behavioural correlates. Owen et al. [12] noted the need for future research to identify correlates at multiple levels, including individual; social; organizational/community; environmental; and policy levels. In addition, previous correlate studies focused on a small number of SB contexts. The existing literature on correlates of SB are different for adolescents, adults and older adults [12]. To our knowledge there are currently no questionnaires identifying potential social-ecological correlates of all relevant contexts of SB in adolescents, adults and older adults.

Gathering information on context-specific SB and its correlates is mostly done using self-report measurements such as questionnaires or diaries [13]. SB questionnaires can be used from the age of 12 (i.e. adolescents) and are commonly used in large-scale observational studies [18]. In addition, questionnaires (self-report) are cost-effective, easily accessible to a large proportion of the population and participant burden is relatively low [19]. However, questionnaires can be susceptible to bias caused by cultural norms, social desirability and/or recall [19, 20]. Despite these drawbacks, questionnaires are more pragmatic and scalable than objective measurement tools in large-scale observational studies.

To address these gaps, we developed three age-specific questionnaires (adolescents, adults and older adults) in order to identify context-specific SB and its determinants/correlates. These age-specific questionnaires were based on the ‘ecologic model of four domains of SB’ [12], the Theory of Planned Behaviour (TPB) [21] and a consensus taxonomy of SBs [11], as they include multiple SB contexts (SB domains: leisure time, household, occupation and transport) and their potential correlates at multiple levels (e.g. attitude and subjective norm of TPB). The main goal of the current study was to test if the newly developed questionnaires were reliable and valid to determine context-specific SB and its potential correlates and total sedentary time in three different age groups. The objectives of the current study were to: (a) analyse the test-retest reliability of questionnaires designed to assess self-reported total sedentary time (for weekdays, weekend days and average days), context-specific SBs (for weekdays and weekend days) and their potential social-ecological correlates in adolescents, adults and older adults; and (b) assess the criterion validity of these questionnaires for self-reported total weekday, weekend day and average day sedentary time.

## Methods

### Subjects and procedures

Sampling approaches were different for the reliability and validity study. In both studies, data were collected in 2013–2014 among adolescents, adults and older adults. An information letter or a verbal explanation was provided to adolescents, adults and older adults participating in the studies. For adolescents parental permission was sought. The studies were approved by the Ghent University Hospital Ethics Committee.

#### Test-retest reliability

Twenty adolescents, 22 adults and 20 older adults, living in Flanders (Belgium), were recruited by using purposeful convenience sampling. Relatives and acquaintances of the research team were contacted to participate in the test-retest reliability study. Additional participants were recruited through purposive snowball sampling, so that participants with different backgrounds (e.g. living environment and socio-economic status) were invited to participate in the study. All participants completed the same questionnaire on context-specific SB and its potential correlates twice. In line with Duncan et al. [22], at least a 6 days interval elapsed between both measurements (mean interval: adolescents = 16 ± 9 days; adults = 14 ± 5 days; older adults = 9 ± 1 days). Adolescents and adults self-completed a paper version of the questionnaire at home (test-retest design). For adolescents and adults, the second questionnaire (retest) was delivered by a researcher when collecting the first questionnaire, so that replication was not possible. The researcher explained when the second questionnaire should be completed and wrote this date on the questionnaire. For older adults, structured interviews were conducted at home by trained researchers to collect data on both occasions.

#### Criterion validity

Eighty-one adolescents, 41 adults and 37 older adults, participated in the current criterion validity study. Adolescents were recruited via schools and adults and older adults were recruited via a city’s public service department in Flanders (Belgium). Participants were asked to wear a movement monitor (activPAL™) for 7 days. Afterwards they were asked to complete an age-specific questionnaire on context-specific SB and its correlates.

For the adolescent group, principals of four schools randomly selected one class per school to participate in the current validity study (participation rate = 100 %). A researcher delivered the movement monitors and explained how to use them to the selected classes. The adolescents wore the movement monitor for seven consecutive days (starting the day after the explanation). On the 8^th^ day the monitors were recollected and the questionnaire was filled out at school under supervision of a researcher (paper-pencil; duration: 30–45 min).

Adults’ contact information (full name, address and date of birth) was gathered via the public service department of the city of Sint-Niklaas (metropolitan city with approximately 73,000 inhabitants in Flanders). A randomly selected sample (*n* = 41) of adults willing to wear a movement monitor was contacted by telephone (participation rate = 64.2 %). At the first appointment at the participants’ homes, a researcher delivered the movement monitor and explained the purpose of the criterion validity study. The day after this visit, participants were asked to wear the movement monitor for seven consecutive days. On the 8^th^ day a researcher recollected the movement monitor at the participants’ homes and the questionnaire was completed by the participants on the same day (paper-pencil; duration: 30–45 min).

Contact information (full name, address and date of birth) from older adults was also gathered via the public service department of Sint-Niklaas. Individuals (65 years or older and living independently or in a service flat) were invited by telephone to take part in the study. Thirty-seven older adults agreed to participate and were subsequently visited at their home (participation rate = 37.4 %). At the first appointment at the participants’ homes, a researcher delivered the movement monitor and explained the purpose of the criterion validity study. The day after this visit, participants were asked to wear the movement monitor for seven consecutive days. On the 8^th^ day a researcher recollected the movement monitor at the participants’ homes and the questionnaire was completed on the same day through a structured interview led by a trained researcher (duration: 60–90 min).

### Measures

#### Questionnaires

The questionnaires were developed to assess context-specific SB and its correlates in three different age groups, namely adolescents, adults and older adults (see Additional files 1, 2, 3; Dutch questionnaires). In these questionnaires (and during structured interviews in case of older adults) participants received information about how they should report their behaviours, so that every period of sitting was only reported once (i.e. not duplicating SBs in case of simultaneous behaviours such as reading while listening to the radio).

The questionnaires consisted of three sections, namely (I) SB, (II) potential correlates of context-specific SB and (III) sedentary-related equipment and simultaneous behaviour variables. The content of each section is described below and details are shown in Additional files 4, 5, 6. All these items were included in the test-retest reliability analyses.

I. Sedentary behaviour
  Context-specific SB (adolescents: 12 contexts; adults: 11 contexts; older adults: 12 contexts) was assessed based on the last-7-day SB questionnaire (SIT-Q-7d) [13]. The SIT-Q-7d (Dutch version) has fair-to-good test-retest reliability (ICC = 0.68) and high criterion validity (Spearman’s rho = 0.52) when measuring total sedentary time (summing up all relevant SB contexts) in a Belgian adult population [13]. An overview of the included SB contexts in the current study for each age group is shown in Table 1. Participants of all age groups were asked to indicate how much time they spent sitting/lying down during different SBs in the last 7 days (weekday and weekend day separately). For adults, work was included for 5 days. For each age group, the variable ‘self-reported total sedentary time’ was calculated by summing all midpoint values of the age-specific SB contexts (separately for weekday and weekend day). Self-reported total sedentary time was calculated for an average day, using the following formula: ((self-reported total sedentary time on a weekday * 5) + (self-reported total sedentary time on a weekend day * 2))/7.

**Table 1:** Overview of the included sedentary behaviour contexts (behaviour-specific questions)

	Adolescents	Adults	Older adults
TV (WK, WKND)^(a)^	V	V	V
Computer (WK, WKND)^(a)^	V	V	V
Sitting while reading (WK, WKND)^(a)^	V	V	V
Sitting for hobbies (WK, WKND)^(a)^	V	V	V
Sitting for socializing (WK, WKND)^(a)^	V	V	V
Sitting while listening to music (WK, WKND)^(a)^	V	V	V
Sitting during meals (WK, WKND)^(a)^	V	V	V
Motorized transport (to and from school, WK, WKND)^(b)^	V	-	-
Motorized transport (to and from occupation, during occupation, WK, WKND)^(b)^	-	V	-
Motorized transport (WK, WKND)^(b)^	-	-	V
Sitting during school work at home (WK, WKND)^(a)^	V	-	-
Sitting while caring ((grand)children, family members,…) (WK, WKND)^(a)^	-	V	V
Sitting for using mobile phone (WK, WKND)^(a)^	V	-	-
Household tasks and making phone calls (WK, WKND)^(a)^	-	V	-
Making phone calls (WK, WKND)^(a)^	-	-	V
School (WK)^(a)^	V	-	-
Occupation^(c)^	-	V	-
Household tasks (WK, WKND)^(a)^	-	-	V
Gaming (WK, WKND)^(a)^	V	-	-
Afternoon nap (WK, WKND)^(a)^	-	-	V

*WK* weekdays, *WKND* weekend days, *V* present in the age-specific questionnaire, *−* not present in the age-specific questionnaire

Answer categories:

^(a)^‘None’, ‘1-15 min/day’, ‘15-30 min/day’, ‘30-60 min/day’, ‘1-2 h/day’, ‘2-3 h/day’, ‘3-4 h/day’, ‘4-5 h/day’, ‘5-6 h/day’, ‘6-7 h/day’ or ‘more than 7 h/day’

^(b)^‘None’, ‘1-15 min/day’, ‘15-30 min/day’, ‘30-45 min/day’, ‘45-60 min/day’, ‘60-90 min/day’, ‘90-120 min/day’, ‘2-2.5 h/day’, ‘2.5-3 h/day’, ‘3-4 h/day’, ‘4-5 h/day’, ‘5-6 h/day’, ‘6-7 h/day’ or ‘more than 7 h/day’

^(c)^‘Less than 2 h/day’, ‘2-3 h/day’, ‘3-4 h/day’, ‘4-5 h/day’, ‘5-6 h/day’, ‘6-7 h/day’, ‘7-8 h/day’, ‘more than 8 h/day’

II. Potential correlates of context-specific SB
  Potential (social-ecological) correlates of context-specific SB were assessed for the following contexts: TV viewing, gaming, computer use, motorized transport and school in adolescents; TV viewing, computer use, motorized transport, occupation, household tasks and making phone calls in adults; and TV viewing, computer use, motorized transport, household tasks and making phone calls in older adults. The following items of the newly developed questionnaires were based on existing questionnaires: psychosocial items regarding TV, gaming, computer, occupation, household tasks and making phone calls, and motorized transport [23]; questions regarding modelling and/or rules for screen-related behaviours [24]; hours/min of physical education at school [25]; occupation-related information [26]. The answer categories of the psychosocial items (ranging from ‘strongly disagree’ to ‘strongly agree’ OR ranging from ‘I consider it perfectly impossible’ to ‘I consider it perfectly possible’) and modelling (e.g. ranging from ‘never’ to ‘very often’) all utilised five-point Likert scales or continuous scales. The items regarding rules for screen-related behaviours required dichotomous yes/no responses.

III. Sedentary-related equipment and simultaneous behaviour variables
  The items related to sedentary-related equipment (i.e. (non-)portable electronic devices at home, in the bedroom) were based on an existing questionnaire [27] and categories of simultaneous behaviour (bivariate combinations of e.g. watching TV, using mobile phone, using computer/tablet, listening to music, having a conversation) using five-point Likert scales (ranging from ‘never’ to ‘very often’).

#### Movement monitor (criterion measure for validity study)

Total SB (weekdays and weekend days) was objectively measured using the activPAL™ movement monitor (activPAL software version 6.4.1 and 7.2.29). This electronic inclinometer has already been validated in adolescents [28], adults [29] and older adults [30], and is recommended when measuring SB [31]. The activPAL™ (15 grams, 53 × 35 × 7 mm) was worn on the thigh and made waterproof prior to the wearing period by covering it with medical transparent tape (i.e. 3M Tegaderm™, 3M Healthcare, St. Paul, MN). These waterproof attachments ensured that participants could wear the monitor during water activities (e.g. bathing and swimming), allowing continuous wear. Data from participants who wore the activPAL™ for at least 4 days (including 1 weekend day) was included in the analysis of the validity study. Participants recorded non-wear time (supplemented with reason) and the time of getting up and going to sleep in a diary. Objectively measured total SB (during waking hours) was also calculated for an average day, using the following formula: ((total SB on a weekday * 5) + (total SB on a weekend day * 2))/7.

Twelve adolescents were excluded from the analyses (technical problems activPAL™: *n* = 2; not meeting inclusion criteria regarding number of wearing days: *n* = 2; not wearing the activPAL™: *n* = 8). Furthermore, 4 adolescents were not present during distribution of the movement monitor and 3 adolescents were absent for filling out the questionnaire on the 8^th^ day. In the adults group, 8 participants were excluded from the analyses (no questionnaire returned: *n* = 2; ill while wearing monitor: *n* = 1; not filling out the questionnaire on the 8^th^ day: *n* = 5). For older adults, 4 participants were excluded from the analyses (no/incorrect diary: *n* = 2; not filling out the questionnaire on the 8^th^ day due to illness of participants: *n* = 2). This resulted in a final sample of 62 adolescents, 33 adults and 33 older adults for the criterion validity study.

### Statistical analyses

All analyses were conducted using IBM SPSS Statistics version 22 (SPSS Inc., Chicago, IL) and alpha levels of p < 0.05 were considered as significant.

#### Participants’ characteristics

Sample characteristics of adolescents, adults and older adults participating in the test-retest reliability study or the criterion validity study were reported as means ± SD (continuous variables) or percentages (categorical variables).

#### Test-retest reliability

Test-retest reliability was calculated for all included individual ‘raw’ items, separately for the three age groups. Furthermore, test-retest reliability was also assessed for self-reported total sedentary time on a weekday, weekend day and an average day. Three statistical tests were used for assessing the agreement between the first and second measurement. Kappa coefficients (κ) for dichotomous and qualitative variables, Intraclass Correlation Coefficients (ICC) for continuous, categorical five-point Likert scales, and percentage agreement (all items) were calculated. With regard to the ICC, two-way random effects single measures were calculated. Classification of reliability (ICC and κ) was interpreted as: ‘poor’ (≤0.40), ‘moderate’ (0.41-0.60), ‘good’ (0.61-0.80) or ‘excellent’ (≥0.81) [24, 32]. Furthermore, percentage agreement was measured, because ICCs and κ are influenced by the presence of variability in response options [24, 33]. Classification of percentage agreement was based on the following criteria: ‘poor’ (<60 %), ‘moderate’ (60-74 %), ‘good’ (75 %-89 %) or ‘excellent’ (90 %-100 %) [24]. Percentage agreement was only interpreted in 3 conditions: I) items with ICC or κ < 0.40, but an agreement > 60 %, II) items with ICC or κ < 0.60, but an agreement > 75 % and III) items with ICC or κ < 0.80, but an agreement > 90 % [24, 34]. Percentage agreement was also reported if the scale has zero variance items or if at least one variable in the analysis was a constant.

#### Criterion validity

The criterion validity of self-reported total sedentary time was determined separately for the three age groups, by calculating Spearman rank correlation coefficients (ρ) between self-reported total sedentary time and activPAL-derived sedentary time (weekday, weekend day and average day separately). Classification of ρ was interpreted as: ‘low’ (<0.30), ‘moderate’ (0.30-0.50) or ‘high’ (>0.50) [35]. In addition to the ρ, absolute agreement between self-reported and objectively measured sedentary time was measured by creating Bland-Altman plots [36]. These plots were created, based on linear regression analyses, through plotting the difference between self-reported total sedentary time and activPAL-derived sedentary time on the y-axis and the average of these self-reported and objectively measured sedentary time on the x-axis (separately for weekday, weekend day and average day). In these plots the trend line of the regression analysis together with the 95 % limits of agreement (LOA) were incorporated, so that conclusions could be made about the absolute agreement between the self-reported total sedentary time and the activPAL-derived sedentary time. However, this parametric approach is only applicable if residuals were normally distributed (examined by using Kolmogorov-Smirnov Test), which was only the case for all data of older adults. As a result, a nonparametric approach was used for all data (weekday, weekend day and average day) of adolescents and adults [36, 37]. In the context of this nonparametric approach, plots were created by plotting the difference between self-reported sedentary time and activPAL-derived sedentary time expressed as a percentage of activPAL-derived sedentary time on the y-axis. This percentage was plotted against activPAL-derived sedentary time on the x-axis.

## Results

### Participants’ characteristics

Sample characteristics (socio demographics) of adolescents, adults and older adults participating in the test-retest reliability study or the criterion validity study are reported in Table 2.

**Table 2:** Sample characteristics of adolescents, adults and older adults included in the test-retest reliability study or the criterion validity study

	Test-retest reliability			Criterion validity		
Items	Adolescents	Adults	Older adults	Adolescents	Adults	Older adults
Numbers (*n*)	20	22	20	62	33	33
Age (years) [m (SD)]	15.39 (1.36)	40.51 (12.39)	73.59 (5.50)	16.14 (1.10)	47.73 (10.51)	72.16 (4.35)
Male Gender (%)	57.90	45.50	50.00	41.90	36.40	60.60
Family situation						
Traditional (mother & father) (%)	75.00	/	/	71.00	/	/
Non-traditional (%)	25.00	/	/	29.00	/	/
Married (%)	/	54.50	85.00	/	66.70	69.70
Widow/widower (%)	/	-	15.00	/	-	9.10
Single (%)	/	4.50	-	/	6.10	21.20
Partner, but living apart (%)	/	9.10	-	/	-	-
Living with *partner* (%)	/	31.80	-	/	27.30	-
Educational level (% tertiary)	/	81.80	75.00	/	63.60	24.20
(Former) occupation						
Household (%)	/	/	20.00	/	/	9.10
White collar (%)	/	85.70	50.00	/	67.70	42.40
Blue collar (%)	/	4.80	30.00	/	25.80	48.50
No occupation (%)	/	9.50	/	/	6.50	/

Sample characteristics of participants in test-retest reliability study were assessed by taking into account the first measurement period

/ (not measured), - (not responded)

### Test-retest reliability

Item-specific results determined in terms of ICC values, kappa values and/or percentage agreement, are shown in Additional file 4 (adolescents), Additional file 5 (adults) and Additional file 6 (older adults). In these additional files, usability of all the individual items for future research is reported. Furthermore, a summary of these results (for each part of the age-specific questionnaires) is reported in Table 3.

**Table 3:** Results of the test-retest reliability study for adolescents, adults and older adults: overview per part of the age-specific questionnaires

Sections of questionnaire (age-specific)	Number of items	ICC (range)	Kappa (range)	Excellent reliability n (%)	Good reliability n (%)	Moderate reliability n (%)	Poor reliability n (%)
Adolescents							
Context-specific sedentary behaviours	24	−0.06; 0.92	/	2 (8.33)	3 (12.50)	10 (41.67)	9 (37.50)
Potential correlates of TV viewing	24	0.49; 0.96	0.44	10 (41.67)	10 (41.67)	4 (16.66)	/
Potential correlates of gaming	19	0.24; 0.92	0.63	4 (21.05)	8 (42.11)	6 (31.58)	1 (5.26)
Potential correlates of computer use	18	0.16; 0.95	0.49	7 (38.89)	5 (27.78)	5 (27.78)	1 (5.55)
Potential correlates of motorized transport	21	0.18; 0.99	0.30; 0.84	8 (38.10)	8 (38.10)	3 (14.28)	2 (9.52)
Potential correlates of school	3	0.57; 0.92	/	1 (33.33)	1 (33.33)	1 (33.33)	/
Sedentary-related equipment	16	0.38; 0.96	/	13 (81.25)	3 (18.75)	/	/
Simultaneous behaviour	6	0.71; 0.88	/	4 (66.67)	2 (33.33)	/	/
Overall	131	−0.06; 0.99	0.30; 0.84	49 (37.41)	40 (30.53)	29 (22.14)	13 (9.92)
Adults							
Context-specific sedentary behaviours	23	0.22; 0.95	/	6 (26.09)	10 (43.48)	6 (26.09)	1 (4.34)
Potential correlates of TV viewing	20	0.31; 0.93	/	7 (35.00)	8 (40.00)	4 (20.00)	1 (5.00)
Potential correlates of computer use	17	0.18; 0.92	/	2 (11.77)	5 (29.41)	5 (29.41)	5 (29.41)
Potential correlates of motorized transport	24	0.42; 1.00	0.29; 1.00	5 (20.83)	12 (50.00)	7 (29.17)	/
Potential correlates of occupation	33	0.06; 0.94	−0.07; 1.00	6 (18.18)	14 (42.43)	9 (27.27)	4 (12.12)
Potential correlates of household tasks and making phone calls	19	0.26; 0.85	0.31; 0.70	4 (21.05)	9 (47.37)	3 (15.79)	3 (15.79)
Sedentary-related equipment	22	0.29; 1.00	/	13 (59.09)	6 (27.27)	3 (13.64)	/
Simultaneous behaviour	6	0.61; 0.87	/	3 (50.00)	3 (50.00)	/	/
Overall	164	0.06; 1.00	−0.07; 1.00	46 (28.05)	67 (40.85)	37 (22.56)	14 (8.54)
Older adults							
Context-specific sedentary behaviours	26	−0.13; 0.95	/	9 (34.62)	7 (26.92)	5 (19.23)	5 (19.23)
Potential correlates of TV viewing	19	−0.19; 1.00	/	5 (26.31)	6 (31.58)	6 (31.58)	2 (10.53)
Potential correlates of computer use	15	−0.15; 0.93	/	4 (26.67)	3 (20.00)	5 (33.33)	3 (20.00)
Potential correlates of motorized transport	17	−0.20; 0.91	0.51	1 (5.88)	5 (29.41)	6 (35.30)	5 (29.41)
Potential correlates of household tasks and making phone calls	18	−0.19; 0.88	0.38; 1.00	5 (27.78)	10 (55.55)	3 (16.67)	/
Sedentary-related equipment	17	−0.07; 1.00	/	12 (70.59)	5 (29.41)	/	/
Simultaneous behaviour	7	0.78; 0.99	/	7 (100.00)	/	/	/
Overall	119	−0.20; 1.00	0.38; 1.00	43 (36.13)	36 (30.25)	25 (21.01)	15 (12.61)

Fifteen, 14 and 25 items (for adolescents, adults and older adults respectively) had low ICC or kappa values, due to low variability in response options, but high percentage agreement (percentage agreement was thus interpreted instead of ICC or kappa values for these items). The test-retest reliability study indicated for adolescents, adults and older adults respectively that 89 items (67.93 %), 113 items (68.90 %) and 79 items (66.38 %) showed good to excellent reliability; 29 items (22.15 %), 37 items (22.56 %) and 25 items (21.01 %) showed moderate reliability. Furthermore, 13 items (9.92 %), 14 items (8.54 %) and 15 items (12.61 %) were found to have poor reliability for adolescents, adults and older adults respectively. With regards to the potential correlates of context-specific SB, the test-retest reliability study for adolescents, adults and older adults had relatively low percentages of items with poor reliability, namely 4.71 % (4/85 items), 11.50 % (13/113 items), and 14.49 % (10/69 items) respectively. Furthermore, the results indicated that 62.50-95.66 % of the context-specific SBs had moderate-to-excellent reliability. More detailed information can be found in Table 3.

Next to the item-specific results, the test-retest reliability for self-reported total sedentary time indicated the following values for weekday, weekend day and an average day respectively: ICC = 0.37 (poor, 95 % confidence interval (CI) = −0.09; 0.70), ICC = 0.67 (good, 95 % CI = 0.32; 0.86), ICC = 0.45 (moderate, 95 % CI = 0.01; 0.74) in adolescents; ICC = 0.77 (good, 95 % CI = 0.52; 0.90), ICC = 0.73 (good, 95 % CI = 0.45; 0.88), ICC = 0.77 (good, 95 % CI = 0.52; 0.90) in adults; ICC = 0.80 (good, 95 % CI = 0.55; 0.92), ICC = 0.68 (good, 95 % CI = 0.34; 0.86), ICC = 0.80 (good, 95 % CI = 0.55; 0.92) in older adults.

### Criterion validity

An overview of the criterion validity results for adolescents, adults and older adults is presented in Table 4. Spearman rank correlation coefficients between self-reported total sedentary time and activPAL™-derived sedentary time showed the following results for a weekday, weekend day and an average day respectively: ρ = 0.42 (moderate, 95 % CI = 0.19; 0.61), ρ = 0.02 (low, 95 % CI = −0.23; 0.27), ρ = 0.29 (low, 95 % CI = 0.04; 0.50) in adolescents; ρ = 0.52 (high, 95 % CI = 0.22; 0.73), ρ = 0.06 (low, 95 % CI = −0.29; 0.40), ρ = 0.49 (moderate, 95 % CI = 0.18; 0.71) in adults; ρ = 0.50 (moderate, 95 % CI = 0.19; 0.72), ρ = 0.38 (moderate, 95 % CI = 0.04; 0.64), ρ = 0.48 (moderate, 95 % CI = 0.16; 0.71) in older adults.

**Table 4:** Overview of the criterion validity results in adolescents, adults and older adults

			Spearman’s rho (95 % CI)	*p*-value	Bland-Altman procedure		
	Self-reported total sedentary time	ActivPAL-derived sedentary time			Regression equation: D = b_0_ + (b_1_ * A)	Standard deviation of the residuals	D at A (95 % LOA)
Adolescents							
Weekday	971.85 (287.65)	618.11 (65.13)	0.42 (0.19; 0.61)	0.001	/	/	/
Weekend day	810.73 (351.56)	567.98 (96.02)	0.02 (−0.23; 0.27)	0.861	/	/	/
Average day	925.82 (289.36)	603.79 (56.34)	0.29 (0.04; 0.50)	0.023	/	/	/
Adults							
Weekday	648.18 (270.12)	479.18 (156.26)	0.52 (0.22; 0.73)	0.002	/	/	/
Weekend day	512.27 (238.02)	447.76 (99.07)	0.06 (−0.29; 0.40)	0.743	/	/	/
Average day	609.35 (242.73)	470.20 (125.46)	0.49 (0.18; 0.71)	0.004	/	/	/
Older adults							
Weekday	515.45 (176.26)	477.67 (111.33)	0.50 (0.19; 0.72)	0.003	−262.14 + (0.60 × A)	139.48	35.80 (−535.51; 11.24)
Weekend day	506.36 (160.32)	551.76 (115.60)	0.38 (0.04; 0.64)	0.030	−288.83 + (0.46 × A)	147.17	−45.46 (−577.29; −0.37)
Average day	512.86 (162.08)	498.84 (102.47)	0.48 (0.16; 0.71)	0.005	−287.19 + (0.60 × A)	126.01	16.32 (−534.18; −40.21)

Total sedentary times are expressed as minutes/day [mean (standard deviation)]

*D* difference between self-reported total sedentary time and activPAL-derived sedentary time*, A* average of self-reported total sedentary time and ActivPAL-derived sedentary time, *b*_*0*_ *i*ntercept*, b*_*1*_ slope*, LOA* limits of agreement (D ± 1.96 × standard deviation of the residuals), *CI* confidence interval*. /* for some variables, Kolmogorov-Smirnov Test was significant, so that Bland-Altman procedures could not be performed

**Fig. 1:**
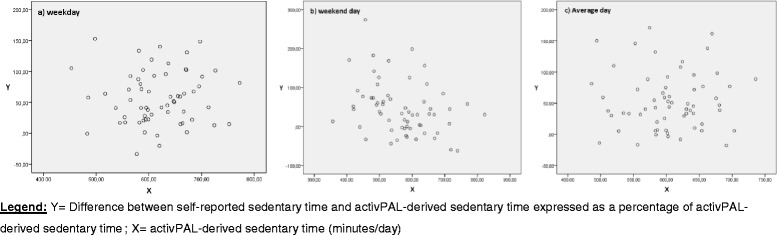
Non-parametric plots for adolescents: Difference between self-reported sedentary time and activPAL-derived sedentary time (%) against activPAL-derived sedentary time, separately for **a** weekday, **b** weekend day, and **c** average day

**Fig. 2:**
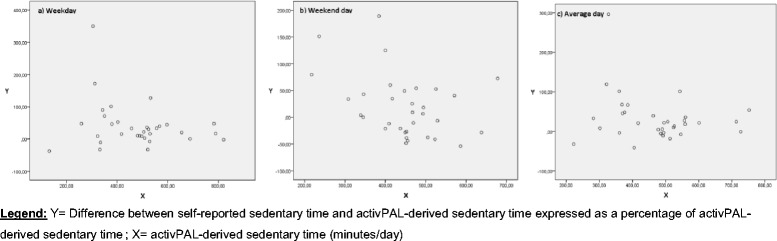
Non-parametric plots for adults: Difference between self-reported sedentary time and activPAL-derived sedentary time (%) against activPAL-derived sedentary time, separately for **a** weekday, **b** weekend day, and **c** average day

Adolescents and adults over-reported their total sedentary time compared to the criterion measurement (activPAL™) for a weekday, weekend day and an average day respectively: +57.05 %, +46.29 %, +53.34 % in adolescents; +40.40 %, +19.15 %, +32.89 % in adults. Accordingly, both adolescents and adults over-reported their total sedentary time more for weekdays than for weekend days. Figure 1a, b and c (adolescents) and Fig. 2a, b and c (adults) illustrate the criterion validity results in more detail (nonparametric approach). For adolescents and adults, weekend days (Figs. 1b and 2b) indicate higher variability at the lowest measured activPAL-derived sedentary time and lower variability at the highest measured activPAL-derived sedentary time. A decrease (expressed in %) in over-reporting using the SB questionnaire was noticed when activPAL-derived sedentary time increased.

**Fig. 3:**
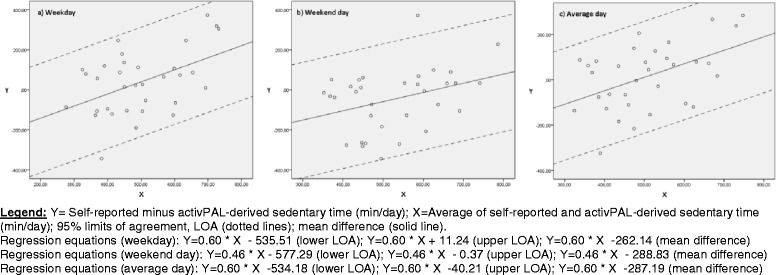
Parametric Bland-Altman plots for older adults: Self-reported total sedentary time and activPAL-derived sedentary time, separately for **a** weekday, **b** weekend day, and **c** average day

Older adults over-reported total sedentary time (except for a weekend day: −6.24 %) compared to the activPAL-derived sedentary time, for a weekday and an average day respectively: +10.10 %, +4.11 %. In Fig. 3a, b and c, in which the average of the two measurement methods was plotted against their difference, positive (significant) relationships were found for weekdays, weekend days and an average day. Differences between self-reported total sedentary time and activPAL-derived sedentary time were calculated by using the following formula recorded in Table 4: D = b_0_ + (b_1_ * A). The averages of the two measurement methods were: 496.56 min/weekday; 529.06 min/weekend day; and 505.85 min/average day. Accordingly, the mean difference between self-reported total sedentary time and activPAL-derived sedentary time for a weekday, weekend day and an average day respectively was: 35.80 min/day; −45.46 min/day; 16.32 min/day. Figure 3a, b and c revealed wide 95 % limits of agreements (3a = −236.20/335.50; 3b = −301.10/271.35; 3c = −248.96/271.11). These figures show that for high averages of self-reported and activPAL-derived sedentary time, self-reported total sedentary time overestimated the objectively measured sedentary time (weekday, weekend day and an average day).

## Discussion

The current study examined the test-retest reliability and criterion validity of three newly developed questionnaires on context-specific SB and its potential correlates and on total sedentary time for adolescents, adults and older adults separately. These questionnaires are innovative as they include all relevant age-specific SB contexts [7, 11, 13, 14] together with potential correlates of context-specific SB.

Test-retest reliability was measured for self-reported total sedentary time and for context-specific SBs (apart for the three included age groups). The results for *reliability of total sedentary time* were comparable with previous research and revealed moderate-to-good reliability, except for total sedentary time on a weekday in adolescents [38]. Low test-retest reliability for sitting on a weekday in adolescents was also found in a study analyzing the International Physical Activity Questionnaire (short version) in youth [39].

Regarding the *reliability of the context-specific SBs*, TV viewing (for adolescents, adults and older adults) and computer use (for adults and older adults) were the most reliable items in the newly developed questionnaires. Present results are in line with previous findings in the literature [7, 10, 40, 41]. This may be because TV viewing is an activity that is more structured and lasts for a longer uninterrupted time, compared to for example activities that occur more irregularly, such as motorized transport [7, 10]. The lowest values for reliability were found for the following items: motorized transport in leisure time on weekend days and sitting during classes at school per day (adolescents), travelling in leisure time on weekend days (adults), sitting while listening to music and sitting for hobbies (older adults). An Australian study measuring the test-retest reliability of the ‘adolescent sedentary activity questionnaire’ also reported lower ICCs for travel (weekend ICCs < weekday ICCs, except for high school girls) compared with the other included SB contexts [42]. Both the present findings (for adolescents and adults) and previous research in adolescents found poorer reliability results for travel during weekend days. This may be explained by the irregular pattern for this type of behaviour during weekend days. The low ICCs for some context-specific SBs might be explained by their between-week variability, as the test and retest did not record the same period [7]. However, the test-retest reliability for total sedentary time was found to be acceptable in all age groups. This suggests that the reliability of total sedentary time might not be considerably influenced by between-week variability, but the contexts of SB vary from week to week (e.g. one weekend self-reported sitting time may be high during motorized transport, but low the next week as the weather was good) [7]. This phenomenon was also present in a Belgian study, reporting good reliability for self-reported total sedentary time (ICC = 0.77) [7].

*Test-retest reliability* was also measured for *the potential correlates* of context-specific SB, *sedentary-related equipment* and *simultaneous behaviour*. In line with the present findings, two European studies in 10- to 12-year-olds and adults reported good test-retest reliability for correlates of screen-viewing behaviour [24, 43]. In all three age groups no single item related to sedentary-related equipment or simultaneous behaviour showed poor reliability. The findings of sedentary-related equipment are in line with the results of a US study reporting ICCs ranging from 0.38-0.88 [27]. The potential correlates regarding TV viewing and school (adolescents), motorized transport (adults), and household tasks and making phone calls (older adults) showed the best reliability. By contrast, SB contexts with the lowest reliability results for potential correlates were motorized transport for adolescents, computer use for adults, and motorized transport for older adults. However, these SB contexts still showed relatively low percentages of items with poor reliability (2.35–7.25 %). Potential correlates of context-specific SB with poor reliability should be used with caution and should not be used as single measures. Such items can however be useful when calculating scales (e.g. average score of items representing attitude towards computer use), but only if the internal consistency is acceptable.

Overall, moderate *validity* was found for *self-reported total sedentary time* measured in the three newly developed questionnaires [35]. Validity results for an average day were fair-to-moderate for adolescents and adults. This level of validity is higher than previously reported in youth [14, 44–46] and comparable [13, 47–49] or better [14, 49–53] than other validity results in adults. To our knowledge, with the exception of four studies [13, 48–50] that used activPAL devices, all of the other validity studies used accelerometers as objective measurement of sitting time. Furthermore, the validity was low for weekend days, but moderate-to-high for weekdays. These results indicate that when assessing total sedentary time, weekend SB may not be suitable as the sole measure. The study of Chastin et al. [50] compared sitting time (IPAQ long-form 7 days) with activPAL measures and found low validity in adults living in the UK (weekdays: *r* = 0.17; weekend days: *r* = 0.01) together with less accurately (underestimated) self-reported sitting time on weekend days compared with weekdays. The difference between weekend days and weekdays is in line with the findings of the current study and may be explained by less structured daily activities during weekend days compared to weekdays, making it more difficult to recall SB in the weekend. In the current study, validity results for older adults were moderate, and found to be comparable [54] or higher [7, 55–58] than other studies in the same age group, except for the computer-delivered 24-h recall, i.e. MARCA (Adult version of the Multimedia Activity Recall for Children and Adolescents) [57]. The higher correlations in the current study can be explained by the use of activPALs as a criterion measure, while accelerometers were used in many of the latter studies. Furthermore, the Bland-Altman plots for older adults revealed that the error may be relative to the average of self-reported and activPAL-derived sedentary time, as the plots suggest to have a ‘turning point’ around 500 min/day on the X-axis (i.e. low averages of self-reported and activPAL-derived sedentary time tend to have more underestimation).

Furthermore, the validity results revealed high percentages of over-reporting for a weekday, weekend day and an average day for adolescents and adults. Similar to our results, the SIT-Q-7d questionnaire overestimated total SB [13]. This may be the result of the inclusion of multiple contexts and thereby the possibility of double-reporting (due to simultaneous behaviour). Nowadays, adolescents and adults sometimes sit while doing several SBs simultaneously (e.g. watching TV and using a computer). Simultaneous behaviour is relatively new and can be present due to technological advances (e.g. the use of tablets, smartphones and laptops). The questionnaires attempted to avoid double-reporting by using several reminders regarding this issue. However, they may not have completely prevented double-reporting. The high percentages of over-reporting could also be caused by adolescents/adults who reported their sitting time more roughly, and therefore over-reported some SB activities as they may not have been capable to remember their SBs from the last seven days. Future studies and questionnaires should take simultaneous behaviour into account in the measurements of SB. An online tool measuring total SB by summing up all relevant domains/contexts of SB with a system of notifications on the screen when participants report unrealistic levels of total SB, is one of the possibilities to avoid over-reporting. Another possibility to tackle the consequences of simultaneous behaviour is the use of structured interviews. A US study by Matthews et al. [59] reported higher validity for adolescents and adults when an interviewer-led ‘previous-day recall (PDR)’ was compared with activPAL measurements (ρ = 0.60-0.81). Similar conclusions can be drawn in the current study, whereby higher validity and consequently lower percentages of over-reporting were found for the structured interviews among older adults than for the paper-pencil questionnaires completed by adolescents and adults, however, more research is needed to confirm that validity is higher when using interviews. Two methodological alterations may be useful to handle over-reporting of self-reported total sedentary time when using these newly developed questionnaires. First, self-reported total sedentary time can be truncated, so that this total sedentary time does not exceed the total waking time based on the average sleeping time reported in other studies enclosing participants with similar characteristics or questions about sleeping time in a sub-study. Secondly, the variables on simultaneous behaviour in the newly developed questionnaires can be used to identify behaviours frequently performed simultaneously, so that one of these SBs can be excluded in the subsequent calculation of self-reported total sedentary time.

A first limitation of the current study is that adults and older adults were free to choose if they would like to wear a movement monitor in the validity study. This may have resulted in self-selection bias (e.g. more motivated people) [60]. It is possible that these individuals were more accurate when filling out the questionnaires (both in reliability study as in validity study). Secondly, adolescents, adults and older adults filled out the questionnaires under different conditions (i.e. home environment versus school environment and paper-pencil versus structured interview), which may have reduced the comparability of the findings. Thirdly, the newly developed questionnaires were long (22–28 pages), so that administration duration was quite high (30–45 min for adolescents/adults and 60–90 min for older adults), however, this was necessary to capture context-specific information. Also, adolescents completed the questionnaire during school hours, however, no information about administration time is available for adults and older adults. Besides, occupational sitting time can be measured using the presented questionnaire. However, in many sectors (e.g. health, retail) work is not limited to weekdays, so for future research the inclusion of questions on occupational sitting time on weekends may be advised. Finally, the answer categories for sedentary time overlap, however, these are based on an existing SB questionnaire [13]. A major strength of the current study is the use of activPAL™ as the criterion measure in the validity study. The use of the activPAL™ for measuring SB has two major advantages: (I) continuous wearing (24 h/day), so limiting non-wear time, (II) classifying posture, so differentiating standing from sitting [13, 29]. Secondly, the recall period in the questionnaire for the SB measurements (last seven days) was identical with the wearing period of the movement monitor, so measuring the same period. Thirdly, the majority of the content of the three newly developed questionnaires was identical for the three age groups, however, age-specific items were incorporated, so that potential age differences between SB contexts can be studied. Finally, the newly developed questionnaires may provide valuable information for future interventions about context-specific SB and its potential correlates.

## Conclusions

All three newly developed questionnaires showed moderate validity for total sedentary time compared to existing questionnaires, except for weekend days in adolescents and adults [38]. However, high percentages of over-reporting of total sedentary time were found for adolescents and adults suggesting that estimations of total SB need to be interpreted with caution in these age groups. More research is needed to minimize over-reporting of total SB when using questionnaires in the future. Total sedentary time had moderate-to-good reliability. The reliability of most of the context-specific SBs was moderate-to-excellent, with screen-related behaviours (i.e. TV viewing and computer use) being the most reliable. Finally, most of the items (i.e. potential correlates of context-specific SBs, sedentary-related equipment and simultaneous behaviour) had moderate-to-excellent reliability. Overall, the newly developed age-specific questionnaires may enhance the knowledge on context-specific SB and its potential correlates.

## Additional files

Additional file 1:
**Questionnaire adolescents (Dutch).** (PDF 177 kb) Additional file 2:
**Questionnaire adults (Dutch).** (PDF 207 kb) Additional file 3:
**Questionnaire older adults (Dutch).** (PDF 248 kb) Additional file 4:
**Results of the test-retest reliability study of the adolescents: Intraclass Correlation Coefficients (ICC), kappa and percentage agreement (item-specific).** (PDF 405 kb) Additional file 5:
**Results of the test-retest reliability study of the adults: Intraclass Correlation Coefficients (ICC), kappa and percentage agreement (item-specific).** (PDF 424 kb) Additional file 6:
**Results of the test-retest reliability study of the older adults: Intraclass Correlation Coefficients (ICC), kappa and percentage agreement (item-specific). **(PDF 395 kb)

## Abbreviations

SB: Sedentary behaviour
METs: Metabolic equivalents
PA: Physical activity
TV: Television
ICC: Intraclass Correlation Coefficient
SIT-Q-7d: Last-7-day sedentary behaviour questionnaire
WK: Weekdays
WKND: Weekend days
SD: Standard deviation
κ: Kappa coefficient
ρ: Spearman rank correlation coefficient
LOA: Limits of agreement
MARCA: Multimedia activity recall for children and adolescents
UK: United Kingdom
PDR: Previous-day recall
IPAQ: International Physical Activity questionnaire
