# Supplementary material for: Reliability and validity of three questionnaires measuring context-specific sedentary behaviour and associated correlates in adolescents, adults and older adults
Source: Int J Behav Nutr Phys Act. 2015 Sep 17;12:117. doi: 10.1186/s12966-015-0277-2 (PMC4574538; doi:10.1186/s12966-015-0277-2)
Supplement: Additional file 1: — Questionnaire adolescents (Dutch). (PDF 177 kb) [file 12966_2015_277_MOESM1_ESM.pdf]

5147

Vragenlijst secundair onderwijs

Het is belangrijk elke vraag goed te lezen en te noteren hoe jij je persoonlijk voelt bij elke stelling. Verder dient deze vragenlijst zo correct mogelijk ingevuld te worden (er zijn geen foute antwoorden). De antwoorden worden vertrouwelijk verwerkt! Je deelname aan deze studie is vrijwillig. Als je deze vragenlijst niet wilt invullen, mag je ons dit zeggen.

Tijdens het invullen van de vragenlijst moet je **rekening** houden met volgende zaken:

- Gelieve slechts **1 antwoord** aan te duiden (indien meerdere antwoorden mogelijk zijn, zal dit bij de vraag vermeld staan).
- Indien er een antwoord voluit geschreven moet worden, gelieve dit in **drukletters** te doen. Bij andere vragen moet je een kruis ("X") in het vakje bij het juiste antwoord plaatsen. Indien je een **antwoord wil veranderen**, laat de "X" staan bij het foute antwoord en maak het vakje met het juiste antwoord volledig zwart.
- Onder **gezinsleden** verstaan we: ouders, broers of zussen, opvoeder,...

We willen je alvast bedanken voor het invullen van deze vragenlijst!

5147

A) ALGEMENE INFORMATIE

Enkele algemene vragen...

ANONIEME VERWERKING!!

- Naam (achternaam & voornaam): ☐☐☐☐☐☐☐☐☐☐☐☐☐☐☐☐☐☐☐☐
- Klas (voorbeeld klas 5A): ☐☐☐☐☐
- Geboortedatum (dag/maand/jaar): ☐☐ / ☐☐ / ☐☐☐☐
- Datum van vandaag (dag/maand/jaar): ☐☐ / ☐☐ / ☐☐☐☐

A1) Geslacht?

☐ Jongen ☐ Meisje

A2) Waar woon je?

☐ Bij je beide ouders
☐ De helft van de tijd bij je moeder, de andere helft bij je vader
☐ Alleen bij je moeder (maar niet bij je vader)
☐ Alleen bij je vader (maar niet bij je moeder)
☐ Bij je moeder en haar nieuwe partner
☐ Bij je vader en zijn nieuwe partner
☐ Bij je grootouders of een ander familielid
☐ In een instelling/op internaat
☐ Andere, verduidelijk: ____________________

A3) Hoeveel broers/zussen heb je?

___ broer(s) en/of ___ zus(sen)

A4) In welke omgeving woon je?

☐ Op het platteland
☐ In een dorp/gemeente
☐ Aan de rand van de stad
☐ In een stad

A5) Ben je het <u>afgelopen jaar</u> verhuisd naar een ander adres?

☐ Ja ☐ Neen

5147

Hoe bekijk je de nabije omgeving van jouw woonplaats (binnen straal van 1 km of op 10-15 minuten wandelen)?

	Helemaal niet akkoord	Eerder niet akkoord	Neutraal	Eerder akkoord	Helemaal akkoord
A6) De meeste woningen in mijn buurt zijn alleenstaande woningen (open bebouwing).	☐	☐	☐	☐	☐
A7) Er zijn veel winkels op wandelafstand van mijn huis.	☐	☐	☐	☐	☐
A8) Er is een halte van het openbaar vervoer (bus en/of tram) op wandelafstand van mijn huis.	☐	☐	☐	☐	☐
A9) Er is een park op wandelafstand van mijn huis.	☐	☐	☐	☐	☐
A10) Wandelen is gevaarlijk omwille van het verkeer in mijn buurt.	☐	☐	☐	☐	☐
A11) Wandelen in mijn buurt is gevaarlijk omwille van de hoge kans op misdaad.	☐	☐	☐	☐	☐
A12) In mijn buurt staan er bomen langs de straten.	☐	☐	☐	☐	☐
A13) Ik vind mijn buurt een goede plaats om in te leven.	☐	☐	☐	☐	☐
A14) De omgeving nodigt uit om te bewegen (geen heuvels, goed onderhouden voetpad,...).	☐	☐	☐	☐	☐

Hoeveel van onderstaande niet draagbare toestellen zijn aanwezig bij jouw thuis EN maak je ZELF ook gebruik van?

	Geen	1	2	3	4	5	Meer dan 5
A15) Televisies	☐	☐	☐	☐	☐	☐	☐
A16) Videospelers/Dvd-spelers	☐	☐	☐	☐	☐	☐	☐
A17) Muziekinstallaties (radio, CD-spelers, stereo,...)	☐	☐	☐	☐	☐	☐	☐
A18) Vaste computer	☐	☐	☐	☐	☐	☐	☐
A19) Spelconsoles op tv (Xbox, Playstation,...)	☐	☐	☐	☐	☐	☐	☐

Hoeveel van deze niet draagbare toestellen bevinden zich in jouw slaapkamer?

	Geen	1	2	3	4	5	Meer dan 5
A20) Televisies	☐	☐	☐	☐	☐	☐	☐
A21) Videospelers/Dvd-spelers	☐	☐	☐	☐	☐	☐	☐
A22) Muziekinstallaties (radio, CD-spelers, stereo,...)	☐	☐	☐	☐	☐	☐	☐
A23) Vaste computer	☐	☐	☐	☐	☐	☐	☐
A24) Spelconsoles op tv (Xbox, Playstation,...)	☐	☐	☐	☐	☐	☐	☐

Gelieve het aantal draagbare toestellen te noteren die aanwezig zijn bij jouw thuis EN waarvan je ZELF ook gebruik maakt.

	Geen	1	2	3	4	5	Meer dan 5
A25) Laptop	☐	☐	☐	☐	☐	☐	☐
A26) GSM (geen smartphone)	☐	☐	☐	☐	☐	☐	☐
A27) Smartphone	☐	☐	☐	☐	☐	☐	☐
A28) Muziekspelers (zoals iPod, MP3,...)	☐	☐	☐	☐	☐	☐	☐
A29)Tablet (iPad, Samsung Galaxy Tab,...)	☐	☐	☐	☐	☐	☐	☐
A30) Spelconsoles (PSP, Game Boy,...)	☐	☐	☐	☐	☐	☐	☐

Het is belangrijk dat je elke periode van zitten of liggen slechts 1 keer in rekening brengt. Indien je bijvoorbeeld 1 uur in de zetel zat om televisie te kijken, maar terwijl ook op de laptop bezig was, reken deze tijd dan als 1 uur televisie kijken indien dit jouw voornaamste bezigheid was. Reken deze tijd dan niet opnieuw als 1 uur computergebruik.

B) TELEVISIE (tv) KIJKEN IN DE VRIJE TIJD

TV kijken (zittend/liggend) = programma's, video's, DVD's en films afgespeeld op TV/computer !

Het spelen van spelletjes (gamen) op tv (Playstation, Xbox,...) telt hier **NIET** mee.

Indien je geen tv kijkt in jouw vrije tijd --> ga naar pagina 8: "S) GAMING (=spelletjes spelen) in de vrije tijd"

B1) Hoe lang zat/lag je gemiddeld neer op één dag tijdens de laatste 7 dagen bij het kijken naar tv in de vrije tijd? Tv-kijken tijdens het eten valt ook hieronder. Beantwoord deze vragen afzonderlijk voor weekdag en weekenddag.

tv-kijken	
**Weekdag**	**Weekenddag**
☐ Niet	☐ Niet
☐ 1 tot 15 minuten/dag	☐ 1 tot 15 minuten/dag
☐ 15 tot 30 minuten/dag	☐ 15 tot 30 minuten/dag
☐ 30 tot 60 minuten/dag	☐ 30 tot 60 minuten/dag
☐ 1 tot 2 uren/dag	☐ 1 tot 2 uren/dag
☐ 2 tot 3 uren/dag	☐ 2 tot 3 uren/dag
☐ 3 tot 4 uren/dag	☐ 3 tot 4 uren/dag
☐ 4 tot 5 uren/dag	☐ 4 tot 5 uren/dag
☐ 5 tot 6 uren/dag	☐ 5 tot 6 uren/dag
☐ 6 tot 7 uren/dag	☐ 6 tot 7 uren/dag
☐ Meer dan 7 uren/dag	☐ Meer dan 7 uren/dag

5147

Gelieve aan te duiden in welke mate je akkoord gaat met elk van onderstaande stellingen.

	Helemaal niet akkoord	Eerder niet akkoord	Neutraal	Eerder akkoord	Helemaal akkoord	Niet van toepassing
B2) Ik vind tv-kijken leuk.	☐	☐	☐	☐	☐	
B3) Tv-kijken verhindert me om andere belangrijkere dingen te doen.	☐	☐	☐	☐	☐	
B4) Ik kijk graag verschillende uren aan een stuk tv.	☐	☐	☐	☐	☐	
B5) Tv-kijken is mijn manier om te ontspannen.	☐	☐	☐	☐	☐	
B6) Mijn gezinsleden vinden dat ik te veel tv kijk.	☐	☐	☐	☐	☐	☐
B7) Ik vind van mezelf dat ik te veel tv kijk.	☐	☐	☐	☐	☐	
B8) Ik zou het zien zitten om de tijd die ik tv kijk te verminderen.	☐	☐	☐	☐	☐	
B9) Ik zou het zien zitten om tijdens het weekend overdag (tot 17 uur) geen tv te kijken.	☐	☐	☐	☐	☐	
B10) Ik zou het zien zitten om de tv niet aan te zetten tijdens het eten.	☐	☐	☐	☐	☐	
B11) Mijn gezinsleden moedigen me aan om minder tv te kijken.	☐	☐	☐	☐	☐	☐
B12) Mijn vrienden moedigen me aan om minder tv te kijken.	☐	☐	☐	☐	☐	

B13) Ik denk dat jongeren van mijn leeftijd beter niet meer dan...

- ☐ 30 minuten tv kijken per dag
- ☐ 1 uur tv kijken per dag
- ☐ 2 uur tv kijken per dag
- ☐ 3 uur tv kijken per dag
- ☐ 4 uur tv kijken per dag
- ☐ 5 uur tv kijken per dag

B14) Waar staat het tv-toestel waar je het meest naar kijkt?

- ☐ In de keuken
- ☐ In de woonkamer
- ☐ In een aparte kamer
- ☐ In de slaapkamer
- ☐ In een andere kamer (noteer) ____________________

B15) Hoeveel tijd kijken jouw gezinsleden gemiddeld naar tv in de vrije tijd?

OUDERS/OPVOEDERS		BROERS en/of ZUSSEN	
☐ Niet van toepassing	☐ 2 tot 3 uur/dag	☐ Niet van toepassing	☐ 2 tot 3 uur/dag
☐ Kijken geen tv	☐ 3 tot 4 uur/dag	☐ Kijken geen tv	☐ 3 tot 4 uur/dag
☐ 1 tot 15 minuten/dag	☐ 4 tot 5 uren/dag	☐ 1 tot 15 minuten/dag	☐ 4 tot 5 uren/dag
☐ 15 tot 30 minuten/dag	☐ 5 tot 6 uren/dag	☐ 15 tot 30 minuten/dag	☐ 5 tot 6 uren/dag
☐ 30 tot 60 minuten/dag	☐ 6 tot 7 uren/dag	☐ 30 tot 60 minuten/dag	☐ 6 tot 7 uren/dag
☐ 1 tot 2 uur/dag	☐ Meer dan 7 uren/dag	☐ 1 tot 2 uur/dag	☐ Meer dan 7 uren/dag

B16) Hoe vaak kijk je SAMEN met jouw gezinsleden naar tv in de vrije tijd?

A) OUDERS/OPVOEDERS

☐ Niet van toepassing ☐ Nooit ☐ Zelden ☐ Soms ☐ Vaak ☐ Heel vaak

B) BROERS en/of ZUSSEN

☐ Niet van toepassing ☐ Nooit ☐ Zelden ☐ Soms ☐ Vaak ☐ Heel vaak

Duid voor elke stelling aan in welke mate je het zou zien zitten om onderstaande zaken uit te voeren.

OPMERKING: Indien je bepaalde zaken al doet --> aanduiden "ik zie dit zeker zitten".

**In welke mate zou je het zien zitten om...**	**Ik zie dit helemaal niet zitten**	**Ik zie dit eerder niet zitten**	**Neutraal**	**Ik zie dit eerder wel zitten**	**Ik zie dit zeker zitten**
B17) taken rechtstaand uit te voeren terwijl je tv aan het kijken bent (licht fysiek actieve rechtsaande activiteiten).	☐	☐	☐	☐	☐
B18) een uur tv-kijken te vervangen door licht fysiek actieve rechtstaande activiteiten.	☐	☐	☐	☐	☐
B19) tijdens de reclame op tv kort recht te staan.	☐	☐	☐	☐	☐
B20) recht te staan om van zender te veranderen.	☐	☐	☐	☐	☐

B21) Hebben je ouders/opvoeders regels over hoeveel uur per dag je tv mag kijken?

☐ Ja ☐ Neen

Hoe beoordeel je volgende beweringen omtrent de voorzieningen bij jou thuis?

("niet van toepassing"= geen tv of geen zetels)

	**Helemaal niet akkoord**	**Eerder niet akkoord**	**Neutraal**	**Eerder akkoord**	**Helemaal akkoord**	**Niet van toepassing**
B22) De afstandsbediening van de tv ligt steeds binnen handbereik wanneer ik deze nodig heb.	☐	☐	☐	☐	☐	☐
B23) De zetels bij ons thuis zijn comfortabel om gedurende een lange tijd in te zitten.	☐	☐	☐	☐	☐	☐

5147

S) GAMING (=spelletjes spelen) in de vrije tijd

Onder gaming verstaan we volgende zaken:
1) zittend spelen van spelconsoles op televisie (Playstation, Xbox, Nintendo,...)
2) zittend spelen van spelletjes op de computer/tablet
3) zittend spelen van spelletjes op GSM/smartphone
==> Het rechtstaand spelen van spelletjes zoals Wii games (Wii Fit,...)/... , telt hier **NIET** mee!
Indien je nooit één van bovenstaande zaken doet, dan mag je verder naar pagina 10: "C) Computergebruik in de vrije tijd".

S1) Hoe lang zat/lag je gemiddeld neer op één dag tijdens de laatste 7 dagen bij gaming in de vrije tijd? Beantwoord deze vraag afzonderlijk voor weekdag en weekenddag.

Weekdag	Weekenddag
☐ Niet	☐ Niet
☐ 1 tot 15 minuten/dag	☐ 1 tot 15 minuten/dag
☐ 15 tot 30 minuten/dag	☐ 15 tot 30 minuten/dag
☐ 30 tot 60 minuten/dag	☐ 30 tot 60 minuten/dag
☐ 1 tot 2 uren/dag	☐ 1 tot 2 uren/dag
☐ 2 tot 3 uren/dag	☐ 2 tot 3 uren/dag
☐ 3 tot 4 uren/dag	☐ 3 tot 4 uren/dag
☐ 4 tot 5 uren/dag	☐ 4 tot 5 uren/dag
☐ 5 tot 6 uren/dag	☐ 5 tot 6 uren/dag
☐ 6 tot 7 uren/dag	☐ 6 tot 7 uren/dag
☐ Meer dan 7 uren/dag	☐ Meer dan 7 uren/dag

Gelieve aan te duiden in welke mate je akkoord gaat met elk van onderstaande stellingen (zittend gamen).

	Helemaal niet akkoord	Eerder niet akkoord	Neutraal	Eerder akkoord	Helemaal akkoord	Niet van toepassing
S2) Ik vind het leuk om te gamen.	☐	☐	☐	☐	☐	
S3) Ik vind het leuk om vele uren te gamen	☐	☐	☐	☐	☐	
S4) Het spelen van games verhindert me belangrijkere zaken te doen.	☐	☐	☐	☐	☐	
S5) Gamen is mijn manier om te ontspannen.	☐	☐	☐	☐	☐	
S6) Mijn ouders zouden blij zijn als ik minder tijd spendeer aan gaming.	☐	☐	☐	☐	☐	
S7) Mijn gezinsleden vinden dat ik te veel tijd spendeer aan gaming.	☐	☐	☐	☐	☐	
S8) Ik vind van mezelf dat ik te veel tijd spendeer aan gaming.	☐	☐	☐	☐	☐	☐
S9) Ik zou het zien zitten om de tijd die ik spendeer aan gaming te verminderen.	☐	☐	☐	☐	☐	
S10) Mijn gezinsleden moedigen me aan om minder te gamen.	☐	☐	☐	☐	☐	
S11) Mijn vrienden moedigen me aan om minder te gamen.	☐	☐	☐	☐	☐	

S12) Hoeveel tijd spenderen jouw gezinsleden gemiddeld al ZITTEND aan gaming in de vrije tijd?

OUDERS/OPVOEDERS		BROERS en/of ZUSSEN	
☐ Niet van toepassing	☐ 2 tot 3 uur/dag	☐ Niet van toepassing	☐ 2 tot 3 uur/dag
☐ Gamen niet	☐ 3 tot 4 uur/dag	☐ Gamen niet	☐ 3 tot 4 uur/dag
☐ 1 tot 15 minuten/dag	☐ 4 tot 5 uren/dag	☐ 1 tot 15 minuten/dag	☐ 4 tot 5 uren/dag
☐ 15 tot 30 minuten/dag	☐ 5 tot 6 uren/dag	☐ 15 tot 30 minuten/dag	☐ 5 tot 6 uren/dag
☐ 30 tot 60 minuten/dag	☐ 6 tot 7 uren/dag	☐ 30 tot 60 minuten/dag	☐ 6 tot 7 uren/dag
☐ 1 tot 2 uur/dag	☐ Meer dan 7 uren/dag	☐ 1 tot 2 uur/dag	☐ Meer dan 7 uren/dag

S13) Hoe vaak ben je ZITTEND aan het gamen SAMEN met onderstaande personen in de vrije tijd?

A) OUDERS/OPVOEDERS

☐ Niet van toepassing ☐ Nooit ☐ Zelden ☐ Soms ☐ Vaak ☐ Heel vaak

B) BROERS en/of ZUSSEN

☐ Niet van toepassing ☐ Nooit ☐ Zelden ☐ Soms ☐ Vaak ☐ Heel vaak

C) VRIENDEN

☐ Niet van toepassing ☐ Nooit ☐ Zelden ☐ Soms ☐ Vaak ☐ Heel vaak

Duid voor elke stelling aan in welke mate je het zou zien zitten om onderstaande zaken uit te voeren.

OPMERKING: indien je bepaalde zaken al doet --> aanduiden "ik zie dit zeker zitten".

**In welke mate zou je het zien zitten om...**	**Ik zie dit helemaal niet zitten**	**Ik zie dit eerder niet zitten**	**Neutraal**	**Ik zie dit eerder wel zitten**	**Ik zie dit zeker zitten**
S14) rechtstaand te gamen.	☐	☐	☐	☐	☐
S15) een uur gamen te vervangen door licht fysiek actieve rechtstaande activiteiten.	☐	☐	☐	☐	☐
S16) na 30 minuten zittend gamen gedurende enkele minuten rechtstaan.	☐	☐	☐	☐	☐

S17) Hebben jouw ouders/opvoeders regels over hoeveel uur per dag je mag gamen?

☐ Ja ☐ Neen

5147

C) COMPUTERGEBRUIK IN DE VRIJE TIJD

De tijd die je op school of thuis voor school aan de computer doorbrengt, telt hier **NIET** mee. Met computergebruik (zittend/liggend) bedoelen we: E-mailen, websites bezoeken, chatten en sociale netwerksites (facebook,...) bezoeken,... Belangrijk is dat je hier ook rekening houdt met het gebruik van **tablets** (handcomputer zoals iPad). Ook internetgebruik op een **smartphone** valt hieronder.

!! Opmerking: Het spelen van spelletjes op de computer telt bij ALLE onderstaande vragen NIET mee!!

Indien je bovenstaande zaken (computer, tablet en smartphone) niet gebruikt --> ga naar pagina 13: "D) Gemotoriseerd transport"

C1) Hoe lang zat/lag je gemiddeld neer op één dag tijdens de laatste 7 dagen bij het gebruik van de computer in de vrije tijd? Beantwoord deze vraag afzonderlijk voor weekdag en weekenddag.

Weekdag	Weekenddag
☐ Niet	☐ Niet
☐ 1 tot 15 minuten/dag	☐ 1 tot 15 minuten/dag
☐ 15 tot 30 minuten/dag	☐ 15 tot 30 minuten/dag
☐ 30 tot 60 minuten/dag	☐ 30 tot 60 minuten/dag
☐ 1 tot 2 uren/dag	☐ 1 tot 2 uren/dag
☐ 2 tot 3 uren/dag	☐ 2 tot 3 uren/dag
☐ 3 tot 4 uren/dag	☐ 3 tot 4 uren/dag
☐ 4 tot 5 uren/dag	☐ 4 tot 5 uren/dag
☐ 5 tot 6 uren/dag	☐ 5 tot 6 uren/dag
☐ 6 tot 7 uren/dag	☐ 6 tot 7 uren/dag
☐ Meer dan 7 uren/dag	☐ Meer dan 7 uren/dag

C2) Hoeveel tijd gebruiken jouw gezinsleden gemiddeld al zittend/liggend een computer in de vrije tijd?

OUDERS/OPVOEDERS		BROERS en/of ZUSSEN	
☐ Niet van toepassing	☐ 2 tot 3 uur/dag	☐ Niet van toepassing	☐ 2 tot 3 uur/dag
☐ Gebruiken geen computer	☐ 3 tot 4 uur/dag	☐ Gebruiken geen computer	☐ 3 tot 4 uur/dag
☐ 1 tot 15 minuten/dag	☐ 4 tot 5 uren/dag	☐ 1 tot 15 minuten/dag	☐ 4 tot 5 uren/dag
☐ 15 tot 30 minuten/dag	☐ 5 tot 6 uren/dag	☐ 15 tot 30 minuten/dag	☐ 5 tot 6 uren/dag
☐ 30 tot 60 minuten/dag	☐ 6 tot 7 uren/dag	☐ 30 tot 60 minuten/dag	☐ 6 tot 7 uren/dag
☐ 1 tot 2 uur/dag	☐ Meer dan 7 uren/dag	☐ 1 tot 2 uur/dag	☐ Meer dan 7 uren/dag

C3) Hoe vaak gebruik je een computer en dit op hetzelfde moment wanneer een ander gezinslid in dezelfde ruimte een andere computer gebruikt in de vrije tijd?

A) OUDERS/OPVOEDERS

☐ Niet van toepassing ☐ Nooit ☐ Zelden ☐ Soms ☐ Vaak ☐ Heel vaak

B) BROERS en/of ZUSSEN

☐ Niet van toepassing ☐ Nooit ☐ Zelden ☐ Soms ☐ Vaak ☐ Heel vaak

Duid voor elke stelling aan in welke mate je het zou zien zitten om onderstaande zaken uit te voeren.

OPMERKING: indien je bepaalde zaken al doet --> aanduiden "ik zie dit zeker zitten".

**In welke mate zou je het zien zitten om...**	**Ik zie dit helemaal niet zitten**	**Ik zie dit eerder niet zitten**	**Neutraal**	**Ik zie dit eerder wel zitten**	**Ik zie dit zeker zitten**
C4) rechtstaand de computer te gebruiken.	☐	☐	☐	☐	☐
C5) een uur computergebruik te vervangen door licht fysiek actieve rechtstaande activiteiten.	☐	☐	☐	☐	☐
C6) na 30 minuten de computer te hebben gebruikt gedurende enkele minuten recht te staan.	☐	☐	☐	☐	☐

C7) Hebben jouw ouders/opvoeders regels over hoeveel uur per dag je de computer mag gebruiken?

☐ Ja ☐ Neen

Gelieve aan te duiden in welke mate je akkoord gaat met elk van de volgende stellingen over computergebruik in de vrije tijd.

	Helemaal niet akkoord	Eerder niet akkoord	Neutraal	Eerder akkoord	Helemaal akkoord	Niet van toepassing
C8) Ik vind het leuk om in de vrije tijd de computer te gebruiken.	☐	☐	☐	☐	☐	
C9) De computer gebruiken verhindert me belangrijkere zaken te doen.	☐	☐	☐	☐	☐	
C10) Ik vind het plezant gedurende een lange tijd de computer te gebruiken.	☐	☐	☐	☐	☐	
C11) De computer gebruiken is mijn manier om te ontspannen.	☐	☐	☐	☐	☐	
C12) Mijn gezinsleden vinden dat ik te veel de computer gebruik.	☐	☐	☐	☐	☐	☐
C13) Ik vind van mezelf dat ik te veel de computer gebruik in mijn vrije tijd.	☐	☐	☐	☐	☐	
C14) Ik zou het zien zitten om de computer gedurende enkele dagen in de week niet te gebruiken in de vrije tijd.	☐	☐	☐	☐	☐	
C15) Ik zou het zien zitten om de tijd die ik spendeer aan computergebruik te verminderen in de vrije tijd.	☐	☐	☐	☐	☐	
C16) Mijn gezinsleden moedigen me aan om minder de computer te gebruiken in de vrije tijd.	☐	☐	☐	☐	☐	☐
C17) Mijn vrienden moedigen me aan om minder de computer te gebruiken in de vrije tijd.	☐	☐	☐	☐	☐	

D) GEMOTORISEERD TRANSPORT

> **1) Onder gemotoriseerd transport verstaan we: auto, brommer, moto, trein, tram of bus.**
> **2) Indien je nooit één van bovenstaande vervoersmiddelen gebruikt (als passagier of als bestuurder) dan mag je verder naar pagina 17: " E) School".**

VERPLAATSINGEN: VAN & NAAR SCHOOL

D1) Hoe lang ZAT je gemiddeld op één dag tijdens de laatste 7 dagen bij gemotoriseerde verplaatsingen van en naar school. Denk aan het gebruik van een auto, bus, trein, tram, moto of brommer; reken fietsen niet mee.

- ☐ Niet
- ☐ 1 tot 15 minuten/dag
- ☐ 15 tot 30 minuten/dag
- ☐ 30 tot 45 minuten/dag
- ☐ 45 tot 60 minuten/dag
- ☐ 60 tot 90 minuten/dag
- ☐ 90 tot 120 minuten/dag
- ☐ 2 tot 2.5 uren/dag
- ☐ 2.5 tot 3 uren/dag
- ☐ 3 tot 4 uren/dag
- ☐ 4 tot 5 uren/dag
- ☐ 5 tot 6 uren/dag
- ☐ 6 tot 7 uren/dag
- ☐ Meer dan 7 uren per dag

VERPLAATSINGEN: IN DE VRIJE TIJD

D2) Hoe lang ZAT je gemiddeld op één dag tijdens de laatste 7 dagen bij gemotoriseerde verplaatsingen BUITEN UW SCHOOLVERPLAATSINGEN. Hou hier dus rekening met jouw transport in de vrije tijd (bv. naar winkel, vrienden opzoeken,...). Denk aan het gebruik van een auto, bus, trein, tram, moto of brommer; reken opnieuw fietsen niet mee. Doe dit apart voor weekdag en weekenddag.

Weekdag	Weekenddag
☐ Niet	☐ Niet
☐ 1 tot 15 minuten/dag	☐ 1 tot 15 minuten/dag
☐ 15 tot 30 minuten/dag	☐ 15 tot 30 minuten/dag
☐ 30 tot 45 minuten/dag	☐ 30 tot 45 minuten/dag
☐ 45 tot 60 minuten/dag	☐ 45 tot 60 minuten/dag
☐ 60 tot 90 minuten/dag	☐ 60 tot 90 minuten/dag
☐ 90 tot 120 minuten/dag	☐ 90 tot 120 minuten/dag
☐ 2 tot 2.5 uren/dag	☐ 2 tot 2.5 uren/dag
☐ 2.5 tot 3 uren/dag	☐ 2.5 tot 3 uren/dag
☐ 3 tot 4 uren/dag	☐ 3 tot 4 uren/dag
☐ 4 tot 5 uren/dag	☐ 4 tot 5 uren/dag
☐ 5 tot 6 uren/dag	☐ 5 tot 6 uren/dag
☐ 6 tot 7 uren/dag	☐ 6 tot 7 uren/dag
☐ Meer dan 7 uren per dag	☐ Meer dan 7 uren per dag

D3) Gelieve aan te geven hoeveel tijd je gemiddeld per dag spendeerde aan wandelen buitenshuis op een rustig tempo (bv: slenteren = rustig stappen, wandelen op "gemak", niet gehaast) als deel van jouw transport (bv. iemand bezoeken, naar bus wandelen, naar winkel wandelen,...) tijdens de laatste 7 dagen. Let op: het wandelen moet dus een doel hebben (wandelen als ontspanning telt hier niet mee).

Weekdag	Weekenddag
☐ Niet	☐ Niet
☐ 1 tot 15 minuten/dag	☐ 1 tot 15 minuten/dag
☐ 15 tot 30 minuten/dag	☐ 15 tot 30 minuten/dag
☐ 30 tot 45 minuten/dag	☐ 30 tot 45 minuten/dag
☐ 45 tot 60 minuten/dag	☐ 45 tot 60 minuten/dag
☐ 1 tot 2 uur/dag	☐ 1 tot 2 uur/dag
☐ Meer dan 2 uur/dag	☐ Meer dan 2 uur/dag

Om onderstaande vragen te beantwoorden dien je rekening te houden met jouw transport in de **vrije tijd** (naar winkel,...) EN jouw transport naar **school**.

Gelieve aan te duiden in welke mate je akkoord gaat met elk van onderstaande stellingen.

	Helemaal niet akkoord	Eerder niet akkoord	Neutraal	Eerder akkoord	Helemaal akkoord	Niet van toepassing
D4) Ik vind het leuk om me met een gemotoriseerd voertuig te verplaatsen.	☐	☐	☐	☐	☐	
D5) Ik vind het leuk om als passagier tijdens gemotoriseerd transport aan bepaalde zaken te werken of uit te rusten (bv. schoolwerk, telefoneren,...).	☐	☐	☐	☐	☐	☐
D6) Ik voel me lui als ik op mijn bestemming aankom na mijn gemotoriseerd transport.	☐	☐	☐	☐	☐	
D7) Ik vind van mezelf dat ik te veel gebruik maak van gemotoriseerd transport.	☐	☐	☐	☐	☐	
D8) Ik zou het zien zitten om spontaan een halte vroeger uit te stappen uit tram/bus en het resterende eind te wandelen.	☐	☐	☐	☐	☐	
D9) Ik zou het zien zitten om spontaan de fiets te nemen of te wandelen ook al kan ik gebruik maken van tram/bus of meerijden met auto.	☐	☐	☐	☐	☐	
D10) Mijn gezinsleden moedigen me aan om me op een (meer) actieve manier (fietsen, wandelen) te verplaatsen.	☐	☐	☐	☐	☐	☐
D11) Mijn vrienden moedigen me aan om me op een (meer) actieve manier (fietsen, wandelen) te verplaatsen.	☐	☐	☐	☐	☐	
D12) Mijn gezinsleden vinden dat ik te veel gebruik maak van gemotoriseerd transport.	☐	☐	☐	☐	☐	☐

5147

D13) Wat zijn voor jou de voornaamste redenen om niet te wandelen of niet te fietsen? (meerdere antwoorden zijn mogelijk)

- ☐ De omgeving is niet veilig om te wandelen of te fietsen
- ☐ Actief transport (wandelen of fietsen) is niet aantrekkelijk (geen groene omgeving in de buurt, saai,...)
- ☐ Actief transport is vermoeiend (zweten, bagage dragen,...)
- ☐ De voorzieningen voor openbaar vervoer zijn goed uitgerust (bushaltes dicht bij de deur,...)
- ☐ Tijdsdruk
- ☐ Te grote afstand
- ☐ Weersomstandigheden
- ☐ Het combineren van verschillende taken (naar bakker, iemand bezoeken,....)
- ☐ Financiële redenen (goedkoper tarief studenten,...)
- ☐ Andere (noteer) ____________________
- ☐ Ik verplaats me altijd actief (wandelen en/of fietsen)

D14) Hoe verplaatsen jouw gezinsleden zich meestal...

a) naar het werk of school?

	Niet van toepassing	Gemotoriseerd transport	Actief transport (wandelen/fietsen)
Ouders/opvoeders	☐	☐	☐
Broers/zussen	☐	☐	☐

b) in de vrije tijd?

	Niet van toepassing	Gemotoriseerd transport	Actief transport (wandelen/fietsen)
Ouders/opvoeders	☐	☐	☐
Broers/zussen	☐	☐	☐

Duid voor elke stelling aan of je het zou zien zitten om onderstaande zaken uit te voeren.

OPMERKING: Indien je bepaalde zaken al doet --> aanduiden "ik zie dit zeker zitten".

In welke mate zou je het zien zitten om:	Ik zie dit helemaal niet zitten	Ik zie dit eerder niet zitten	Neutraal	Ik zie dit eerder wel zitten	Ik zie dit zeker zitten
D15) spontaan recht te staan op de bus, trein of tram in plaats van neer te zitten.	☐	☐	☐	☐	☐
D16) na 30 minuten zitten in bus, trein of tram even kort te gaan rechtstaan.	☐	☐	☐	☐	☐

D17) Hoe vaak verplaats je je SAMEN met jouw gezinsleden met gemotoriseerd transport? Doe dit apart voor transport van/naar het werk of school en transport in de vrije tijd (afzonderlijk voor weekdag en weekenddag).

Van/naar het werk of school	
**Ouders/opvoeders**	**Broers/zussen**
☐ Niet van toepassing<br>☐ Nooit<br>☐ Zelden<br>☐ Soms<br>☐ Vaak<br>☐ Heel vaak	☐ Niet van toepassing<br>☐ Nooit<br>☐ Zelden<br>☐ Soms<br>☐ Vaak<br>☐ Heel vaak

Vrije tijd			
**Ouders/opvoeders**		**Broers/zussen**	
**Weekdag**	**Weekenddag**	**Weekdag**	**Weekenddag**
☐ Niet van toepassing<br>☐ Nooit<br>☐ Zelden<br>☐ Soms<br>☐ Vaak<br>☐ Heel vaak	☐ Niet van toepassing<br>☐ Nooit<br>☐ Zelden<br>☐ Soms<br>☐ Vaak<br>☐ Heel vaak	☐ Niet van toepassing<br>☐ Nooit<br>☐ Zelden<br>☐ Soms<br>☐ Vaak<br>☐ Heel vaak	☐ Niet van toepassing<br>☐ Nooit<br>☐ Zelden<br>☐ Soms<br>☐ Vaak<br>☐ Heel vaak

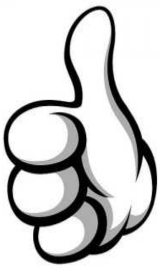

Je bent al voorbij de helft van de vragen

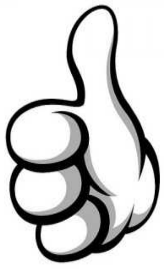

5147

E) SCHOOL

E1) Hoeveel tijd bracht je gemiddeld per dag al zittend door tijdens de lesuren op school tijdens de laatste 7 dagen? De tijd die je thuis doorbrengt in functie van school telt hier NIET mee.

- ☐ Niet
- ☐ 1 tot 15 minuten/dag
- ☐ 15 tot 30 minuten/dag
- ☐ 30 tot 60 minuten/dag
- ☐ 1 tot 2 uren/dag
- ☐ 2 tot 3 uren/dag
- ☐ 3 tot 4 uren/dag
- ☐ 4 tot 5 uren/dag
- ☐ 5 tot 6 uren/dag
- ☐ 6 tot 7 uren/dag
- ☐ Meer dan 7 uren/dag

E2) Hoeveel uur lichamelijke opvoeding of sport krijg je tijdens de week op school (= echte lesuren, GEEN naschoolse activiteiten)? (reken hier ook de zwemlessen bij)

☐☐ uur/uren ☐☐ minuten per week

E3) Wat doe je meestal tijdens de speeltijd? (1 antwoord)

- ☐ Zitten
- ☐ Staan
- ☐ Wandelen
- ☐ Sporten/spelen
- ☐ Er is geen speeltijd

E4) Wat doe je meestal tijdens de middagpauze? (1 antwoord)

- ☐ Zitten
- ☐ Staan
- ☐ Wandelen
- ☐ Sporten/spelen
- ☐ Er is geen middagpauze

F) ANDERE ZITTENDE/LIGGENDE ACTIVITEITEN

Hoe lang **zat/lag je gemiddeld op één dag** tijdens de **laatste 7 dagen** voor de volgende **zittende/liggende activiteiten**?

OPMERKING: A) MAALTIJDEN --> eten voor tv moet men hier **niet** meetellen & B) SCHOOLWERK --> huiswerk maken-studeren-werken op de computer voor school,... & C) zittend LEZEN--> enkel het lezen van papieren versies

		**Niet**	**1 tot 15 minuten per dag**	**15 tot 30 minuten per dag**	**30 tot 60 minuten per dag**	**1 tot 2 uur per dag**	**2 tot 3 uur per dag**	**3 tot 4 uur per dag**	**4 tot 5 uur per dag**	**5 tot 6 uur per dag**	**6 tot 7 uur per dag**	**Meer dan 7 uur per dag**
**F1) Zittend lezen - niet voor school!** (boek, tijdschrift,...)	weekdag	☐	☐	☐	☐	☐	☐	☐	☐	☐	☐	☐
	weekenddag	☐	☐	☐	☐	☐	☐	☐	☐	☐	☐	☐
**F2) Zitten bij hobby's** (vrijwilligers-werk, tekenschool,...)	weekdag	☐	☐	☐	☐	☐	☐	☐	☐	☐	☐	☐
	weekenddag	☐	☐	☐	☐	☐	☐	☐	☐	☐	☐	☐
**F3) Zitten bij sociaal contact** (vrienden bezoeken, cinema,...)	weekdag	☐	☐	☐	☐	☐	☐	☐	☐	☐	☐	☐
	weekenddag	☐	☐	☐	☐	☐	☐	☐	☐	☐	☐	☐
**F4) Zittend naar muziek luisteren** (radio, MP3,...)	weekdag	☐	☐	☐	☐	☐	☐	☐	☐	☐	☐	☐
	weekenddag	☐	☐	☐	☐	☐	☐	☐	☐	☐	☐	☐
**F5) Zitten tijdens maaltijden** (ontbijt-avond)	weekdag	☐	☐	☐	☐	☐	☐	☐	☐	☐	☐	☐
	weekenddag	☐	☐	☐	☐	☐	☐	☐	☐	☐	☐	☐
**F6) Zitten bij schoolwerk** thuis uitgevoerd	weekdag	☐	☐	☐	☐	☐	☐	☐	☐	☐	☐	☐
	weekenddag	☐	☐	☐	☐	☐	☐	☐	☐	☐	☐	☐
**F7) Zittend telefoneren** (bellen & SMS)	weekdag	☐	☐	☐	☐	☐	☐	☐	☐	☐	☐	☐
	weekenddag	☐	☐	☐	☐	☐	☐	☐	☐	☐	☐	☐

Hoe vaak doe je onderstaande situaties op HETZELFDE (tegelijkertijd) moment?

OPMERKING:
A) "niet van toepassing" kan enkel worden aangeduid als je een bepaald toestel niet gebruikt/heeft of één van beide activiteiten niet doet.

	Nooit	Zelden	Soms	Vaak	Heel vaak	Niet van toepassing
F8) tv kijken EN GSM gebruiken	☐	☐	☐	☐	☐	☐
F9) tv kijken EN computer/tablet	☐	☐	☐	☐	☐	☐
F10) computer EN GSM gebruiken	☐	☐	☐	☐	☐	☐
F11) computer EN muziek luisteren	☐	☐	☐	☐	☐	☐
F12) GSM (SMS) EN praten met vrienden/familie	☐	☐	☐	☐	☐	☐
F13) GSM EN muziek luisteren	☐	☐	☐	☐	☐	☐

5147

R) RECHTSTAANDE ACTIVITEITEN

Gelieve aan te geven hoeveel tijd je gemiddeld per dag spendeerde aan de onderstaande rechtstaande activiteiten/situaties tijdens de laatste 7 dagen.

		Niet	1 tot 15 minuten/dag	15 tot 30 minuten/dag	30 tot 45 minuten/dag	45 tot 60 minuten/dag	1 tot 2 uur/dag	Meer dan 2 uur/dag
**ONTSPANNING (RECHTSTAAND UITGEVOERD)**								
**R1) Kleine kinderen** (rustig samen spelen,...)	weekdag	☐	☐	☐	☐	☐	☐	☐
	weekenddag	☐	☐	☐	☐	☐	☐	☐
**TUIN (RECHTSTAAND UITGEVOERD)**								
R2) Planten water geven en/of dieren eten geven,...	weekdag	☐	☐	☐	☐	☐	☐	☐
	weekenddag	☐	☐	☐	☐	☐	☐	☐
**VERZORGING (RECHTSTAAND UITGEVOERD)**								
**R3) Verzorging** (douchen, scheren, make-up, tanden poetsen,...)	weekdag	☐	☐	☐	☐	☐	☐	☐
	weekenddag	☐	☐	☐	☐	☐	☐	☐
**VRIJE TIJD/HOBBY'S (RECHTSTAAND UITGEVOERD)**								
R4) Staand tekenen, staand muziek luisteren, staand SMS'en, feesten (niet dansen), zingen, gitaar spelen	weekdag	☐	☐	☐	☐	☐	☐	☐
	weekenddag	☐	☐	☐	☐	☐	☐	☐
**WANDELEN OP RUSTIG TEMPO (bv. slenteren= rustig stappen, niet gehaast)**								
R5) Wandelen op rustig tempo (bv. slenteren) in huis	weekdag	☐	☐	☐	☐	☐	☐	☐
	weekenddag	☐	☐	☐	☐	☐	☐	☐
**RECHTSTAAN TIJDENS DE LESUREN OP SCHOOL (praktijklessen, practica...)**								
R6) Rechtstaan tijdens lesuren, zoals practica	weekdag	☐	☐	☐	☐	☐	☐	☐

5147

G) GEZONDHEID

Deze vragen gaan over je standpunten ten aanzien van jouw gezondheid. Al je antwoorden worden ook hier **strikt vertrouwelijk verwerkt**!

G1) Wat vind je, over het algemeen genomen, van je gezondheid?

- ☐ Uitstekend
- ☐ Zeer goed
- ☐ Goed
- ☐ Matig
- ☐ Slecht

Hoe vaak heb je in de afgelopen 2 weken last gehad van één of meer van de volgende problemen?

	Helemaal niet	Verscheidene dagen	Meer dan de helft van de dagen	Bijna elke dag
G2) Weinig interesse of plezier in activiteiten	☐	☐	☐	☐
G3) Je neerslachtig, depressief of hopeloos voelen	☐	☐	☐	☐

5147

H) MATE VAN FYSIEKE ACTIVITEIT

Hoe ga je MEESTAL van en naar school? (duid slechts **1** antwoord aan & vul het totaal aantal minuten in = heen & terug opgeteld)

☐ Met de fiets [ ][ ][ ] minuten per dag

☐ Te voet [ ][ ][ ] minuten per dag

☐ Met de auto, trein, bus of motorfiets [ ][ ][ ] minuten per dag

☐ Met de step, autopet, rollerblades, skeelers [ ][ ][ ] minuten per dag

H5) Hoeveel uur besteed je in totaal aan beweeg- of sportactiviteiten die op school worden georganiseerd buiten de turnlessen?
Dit zijn bijvoorbeeld sportactiviteiten tijdens de speeltijd, over de middag, tijdens de opvang, na de schooluren, op woensdagmiddag, op klastornooien,...

PAS OP! Gelieve de turnlessen op school er niet bij te rekenen.

☐ Op school worden geen extra beweeg- of sportactiviteiten gegeven
☐ Af en toe
☐ 1 uur per maand
☐ 2 uur per maand
☐ 3 uur per maand
☐ 1 uur per week
☐ 2 uur per week
☐ 3 uur per week
☐ 4 uur per week
☐ Meer dan 4 uur per week

Hoe verplaats je je MEESTAL in je VRIJE TIJD? Bijvoorbeeld: hoe ga je naar de sportclub, winkel, je vrienden, de film,...

PAS OP! De verplaatsingen van en naar school mag je **niet** meerekenen
Fietsen en wandelen als sport mag je ook **niet** meerekenen.

		Niet	0-10 min	10-20 min	20-30 min	30-40 min	40-50 min	50-60 min	1u-1u10	Meer dan 1u10
**WEEKDAG**	Wandelen	☐	☐	☐	☐	☐	☐	☐	☐	☐
	Fietsen	☐	☐	☐	☐	☐	☐	☐	☐	☐
**WEEKENDDAG**	Wandelen	☐	☐	☐	☐	☐	☐	☐	☐	☐
	Fietsen	☐	☐	☐	☐	☐	☐	☐	☐	☐

Geef de belangrijkste sport (al dan niet in clubverband) die je tijdens je vrije tijd het meest beoefent.

PAS OP! De les lichamelijke opvoeding en sport op school tellen niet mee!

☐ Ik beoefen geen sport

☐ Mijn meest beoefende sport is ________________

Doe je deze sport elke week?

☐ Ja, elke week

Hoeveel keer per week? Plaats een kruisje in 1 vakje en vul ernaast in hoe lang je deze sport per keer doet

☐ 1 keer per week
☐ 2 keer per week
☐ 3 keer per week
☐ 4 keer per week
☐ 5 keer per week ____ uur ____ minuten per keer
☐ 6 keer per week
☐ 7 keer per week
☐ Meer dan 7 keer per week

☐ Neen, niet elke week

Hoe vaak doe je deze sport? Plaats een kruisje in 1 vakje en vul ernaast in hoe lang je deze sport per keer doet

☐ Enkele keren per jaar
☐ 1 keer per maand
☐ 2 keer per maand ____ uur ____ minuten per keer
☐ 3 keer per maand

5147

I) INFORMATIEF GEDEELTE

I1) Wat is jouw moedertaal? (gelieve 1 antwoord te geven)

- ☐ Nederlands
- ☐ Frans
- ☐ Turks
- ☐ Arabisch
- ☐ Engels
- ☐ Andere (noteer) ____________________

I2) In welke onderwijsvorm volg je momenteel les?

- ☐ Kunstsecundair onderwijs (KSO)
- ☐ Beroepssecundair onderwijs (BSO)
- ☐ Technisch secundair onderwijs (TSO)
- ☐ Algemeen secundair onderwijs (ASO)

Wat is het hoogst behaalde diploma of getuigschrift dat jouw ouders behaald hebben/of bezig zijn te behalen?

**VADER** (of inwonende partner van moeder)	**MOEDER** (of inwonende partner van vader)
☐ Niet van toepassing	☐ Niet van toepassing
☐ Weet ik niet	☐ Weet ik niet
☐ Lager onderwijs	☐ Lager onderwijs
☐ Beroeps secundair onderwijs (BSO)	☐ Beroeps secundair onderwijs (BSO)
☐ Technisch secundair onderwijs (TSO)	☐ Technisch secundair onderwijs (TSO)
☐ Algemeen secundair onderwijs (ASO)	☐ Algemeen secundair onderwijs (ASO)
☐ Hoger onderwijs, niet-universitair	☐ Hoger onderwijs, niet-universitair
☐ Universitair onderwijs	☐ Universitair onderwijs

I4) Wat is jouw lichaamslengte (cm)? ______ cm

I5) Wat is jouw lichaamsgewicht (kg)? ______ kg

I6) Hoeveel motorvoertuigen (wagens, moto's of brommers,...) die nog werken, zijn er aanwezig in jouw gezin? Gelieve de voertuigen waarvan je geen gebruik maakt OOK mee te rekenen.

____ (Noteer hier het aantal)

BEDANKT!
