# Supplementary material for: Reliability and validity of three questionnaires measuring context-specific sedentary behaviour and associated correlates in adolescents, adults and older adults
Source: Int J Behav Nutr Phys Act. 2015 Sep 17;12:117. doi: 10.1186/s12966-015-0277-2 (PMC4574538; doi:10.1186/s12966-015-0277-2)
Supplement: Additional file 2: — Questionnaire adults (Dutch). (PDF 207 kb) [file 12966_2015_277_MOESM2_ESM.pdf]

3300

UNIVERSITEIT GENT

Vragenlijst voor inwoners van Sint-Niklaas

Richtlijnen:

Het is belangrijk elke vraag goed te lezen en te noteren hoe u zich persoonlijk voelt bij elke stelling. Verder dient deze vragenlijst zo correct mogelijk ingevuld te worden. De antwoorden worden vertrouwelijk verwerkt!

Tijdens het invullen van de vragenlijst dient u **rekening** te houden met volgende zaken:

- Gelieve telkens slechts **1 antwoord** aan te duiden (indien meerdere antwoorden mogelijk zijn, zal dit bij de vraag vermeld staan).
- Gelieve de vragenlijst in te vullen met een blauwe of zwarte balpen.
- Onder gezinsleden verstaan we: partner, kinderen, oudere inwonende personen,...
- Gelieve onderaan deze pagina uw handtekening (met datum) te plaatsen indien u wenst deel te nemen aan deze studie.
- De antwoordmogelijkheid "*niet van toepassing*" wil zeggen dat deze vraag voor u niet mogelijk is om in te vullen (bv. als u geen kinderen of geen partner heeft).
- Indien er een antwoord voluit geschreven moet worden, gelieve dit in **drukletters** te doen. Bij de andere vragen moet u een kruis ("X") in het vakje bij uw juiste antwoord plaatsen. Indien u een **antwoord wil veranderen**, laat de "X" staan bij het foute antwoord en maak het vakje met het juiste antwoord volledig zwart.
- Er zijn **GEEN** foute antwoorden!

Ik, ... (**naam en voornaam**) ga akkoord, na het lezen van de informatiebrief, om deel te nemen aan de studie over zitten en licht intense activiteiten.

Handtekening (met datum) voor akkoord:

3300

A) ALGEMENE INFORMATIE

Gelieve onderstaande zaken in te vullen in drukletters (anonieme verwerking):

- Initialen van voornaam en achternaam: ☐☐☐☐☐☐
- Geboortedatum (dag/maand/jaar): ☐☐ / ☐☐ / ☐☐☐☐
- Datum van vandaag (dag/maand/jaar): ☐☐ / ☐☐ / ☐☐☐☐
- E-mail adres (indien mogelijk):

A1) Wat is uw geslacht?

☐ Man ☐ Vrouw

A2) Bent u verhinderd om recht te staan (bv. een recente operatie, rugklachten,...)?

☐ Ja ik kan niet rechtstaan ☐ Neen ik kan rechtstaan

Indien ja: De vragenlijst is omwille van uw gezondheidstoestand niet van toepassing voor u. We willen u toch bedanken voor uw interesse! Gelieve deze vragenlijst ook in dit geval zeker op te sturen naar ons.

A3) Hoe kunt u uw huidige gezinssituatie het best omschrijven? (gelieve slechts 1 antwoord aan te duiden)

☐ Vrijgezel
☐ Ik heb een partner, maar we wonen nog apart
☐ Samenwonend met partner
☐ Gehuwd
☐ Weduwe/weduwnaar

A4) Heeft u kinderen?

☐ Neen
☐ Ja

Indien ja, hoeveel kinderen wonen er nog bij u thuis? ___

A5) In welke omgeving woont u?

☐ Op het platteland
☐ In een dorp/gemeente
☐ Aan de rand van de stad
☐ In een stad

A6) Bent u het afgelopen jaar verhuisd naar een ander adres?

☐ Ja ☐ Neen

ZITTEN EN LICHT INTENSE ACTIVITEITEN

Het is belangrijk dat u elke periode van zitten of liggen **slechts 1 keer** in rekening brengt. Indien u bijvoorbeeld 1 uur in de zetel zat om een boek te lezen terwijl u naar muziek luisterde, reken deze tijd dan als 1 uur lezen indien dit uw voornaamste bezigheid was. Reken deze tijd dan niet opnieuw als 1 uur muziek luisteren. Voor elke context dient u aan te duiden hoeveel tijd u hierin spendeerde de **afgelopen 7 dagen** en verder dient u ook uw mening te geven over verschillende situaties per context.

B) TELEVISIE (tv) KIJKEN IN DE VRIJE TIJD

De volgende vragen gaan over zittend/liggend tv-kijken in uw vrije tijd. Met tv-kijken bedoelen we alle zaken (video's, films en gaming) die op tv worden afgespeeld. DVD's en films bekijken op uw computer worden ook als tv-kijken beschouwd. Onder gaming op tv verstaan we bijvoorbeeld het zittend spelen op de Playstation of Xbox.

Indien u geen tv kijkt in uw vrije tijd --> ga naar pagina 6: "*C) computergebruik in de vrije tijd*"

B1) Hoe lang zat u gemiddeld neer op één dag tijdens de laatste 7 dagen bij het kijken naar tv in de vrije tijd? Tv-kijken tijdens het eten valt ook hieronder. Beantwoord deze vragen afzonderlijk voor weekdag en weekenddag.

tv-kijken	
**Weekdag**	**Weekenddag**
☐ Niet	☐ Niet
☐ 1 tot 15 minuten/dag	☐ 1 tot 15 minuten/dag
☐ 15 tot 30 minuten/dag	☐ 15 tot 30 minuten/dag
☐ 30 tot 60 minuten/dag	☐ 30 tot 60 minuten/dag
☐ 1 tot 2 uren/dag	☐ 1 tot 2 uren/dag
☐ 2 tot 3 uren/dag	☐ 2 tot 3 uren/dag
☐ 3 tot 4 uren/dag	☐ 3 tot 4 uren/dag
☐ 4 tot 5 uren/dag	☐ 4 tot 5 uren/dag
☐ 5 tot 6 uren/dag	☐ 5 tot 6 uren/dag
☐ 6 tot 7 uren/dag	☐ 6 tot 7 uren/dag
☐ Meer dan 7 uren/dag	☐ Meer dan 7 uren/dag

3300

Gelieve aan te duiden in welke mate u akkoord gaat met elk van de onderstaande stellingen.

	Helemaal niet akkoord	Eerder niet akkoord	Neutraal	Eerder akkoord	Helemaal akkoord	Niet van toepassing
B2) Ik vind tv-kijken leuk.	☐	☐	☐	☐	☐	
B3) Tv-kijken verhindert me om andere belangrijkere dingen te doen.	☐	☐	☐	☐	☐	
B4) Ik kijk graag verschillende uren aan een stuk tv.	☐	☐	☐	☐	☐	
B5) Tv-kijken is mijn manier om te ontspannen na een werk- of schooldag.	☐	☐	☐	☐	☐	
B6) Mijn gezinsleden vinden dat ik te veel tv kijk.	☐	☐	☐	☐	☐	☐
B7) Ik vind van mezelf dat ik te veel tv kijk.	☐	☐	☐	☐	☐	
B8) Ik zou het zien zitten om de tijd die ik tv kijk te verminderen.	☐	☐	☐	☐	☐	
B9) Ik zou het zien zitten om tijdens het weekend overdag (tot 17 uur) geen tv te kijken.	☐	☐	☐	☐	☐	
B10) Ik zou het zien zitten om de tv niet aan te zetten tijdens het eten.	☐	☐	☐	☐	☐	
B11) Mijn gezinsleden moedigen me aan om minder tv te kijken.	☐	☐	☐	☐	☐	☐
B12) Mijn vrienden moedigen me aan om minder tv te kijken.	☐	☐	☐	☐	☐	

B13) Ik denk dat volwassenen beter niet meer dan...

- ☐ 30 minuten tv kijken per dag
- ☐ 1 uur tv kijken per dag
- ☐ 2 uur tv kijken per dag
- ☐ 3 uur tv kijken per dag
- ☐ 4 uur tv kijken per dag
- ☐ 5 uur tv kijken per dag

B14) Waar staat het tv-toestel waar u het meest naar kijkt? Gelieve slechts 1 antwoord te geven.

- ☐ In de keuken
- ☐ In de woonkamer
- ☐ In een aparte kamer
- ☐ In de slaapkamer
- ☐ In een andere kamer (noteer) ____________________

3300

B15) Hoeveel tijd kijken uw gezinsleden gemiddeld naar tv?

Partner	Kinderen die thuis wonen
☐ Niet van toepassing	☐ Niet van toepassing
☐ Kijkt geen tv	☐ Kijken geen tv
☐ Slechts enkele keren per week	☐ Slechts enkele keren per week
☐ Minder dan 30 minuten/dag	☐ Minder dan 30 minuten/dag
☐ 30 tot 60 minuten/dag	☐ 30 tot 60 minuten/dag
☐ 1 tot 2 uur/dag	☐ 1 tot 2 uur/dag
☐ 2 tot 3 uur/dag	☐ 2 tot 3 uur/dag
☐ 3 tot 4 uur/dag	☐ 3 tot 4 uur/dag
☐ Meer dan 4 uur/dag	☐ Meer dan 4 uur/dag

B16) Hoe vaak kijkt u SAMEN met uw gezinsleden naar tv?

Partner	Kinderen die thuis wonen
☐ Niet van toepassing	☐ Niet van toepassing
☐ Nooit	☐ Nooit
☐ Zelden	☐ Zelden
☐ Soms	☐ Soms
☐ Vaak	☐ Vaak
☐ Heel vaak	☐ Heel vaak

Er zijn verschillende situaties waarin u uw zittend gedrag tijdens tv-kijken kan verminderen of (meer) kan onderbreken door even recht te staan. Duid voor elke stelling aan of u het zou zien zitten om volgende zaken uit te voeren.

In welke mate zou u het zien zitten om:	Ik zie dit helemaal niet zitten	Ik zie dit eerder niet zitten	Neutraal	Ik zie dit eerder wel zitten	Ik zie dit zeker zitten
B17) taken rechtstaand uit te voeren terwijl u tv aan het kijken bent (zoals strijken, klusjes uitvoeren,...).	☐	☐	☐	☐	☐
B18) een uur tv-kijken te vervangen door lichte inspanningen (vb: kuisen, klusjes).	☐	☐	☐	☐	☐
B19) tijdens de reclame op tv kort recht te staan.	☐	☐	☐	☐	☐

C) COMPUTERGEBRUIK IN DE VRIJE TIJD

De volgende vragen gaan over uw zittend computergebruik in de vrije tijd. De tijd die u voor het werk of thuis voor uw job aan de computer doorbrengt, telt hier **NIET** mee. Met computergebruik bedoelen we: E-mailen, websites bezoeken, chatten, sociale netwerksites (facebook,...) bezoeken, spelletjes spelen,... Belangrijk is dat u hier ook rekening houdt met het gebruik van **tablets** (handcomputer zoals iPad). Ook internetgebruik op een **smartphone** valt hieronder.

Indien u bovenstaande zaken (computer, tablet en smartphone) niet gebruikt in uw vrije tijd --> ga naar **pagina 8: "D)** ***gemotoriseerd transport als woon-werkverplaatsing en in de vrije tijd"***

C1) Hoe lang zat u gemiddeld neer op één dag tijdens de laatste 7 dagen bij het gebruik van de computer in de vrije tijd? Beantwoord deze vraag afzonderlijk voor weekdag en weekenddag.

**Computer gebruiken in de vrije tijd**	
**Weekdag**	**Weekenddag**
☐ Niet	☐ Niet
☐ 1 tot 15 minuten/dag	☐ 1 tot 15 minuten/dag
☐ 15 tot 30 minuten/dag	☐ 15 tot 30 minuten/dag
☐ 30 tot 60 minuten/dag	☐ 30 tot 60 minuten/dag
☐ 1 tot 2 uren/dag	☐ 1 tot 2 uren/dag
☐ 2 tot 3 uren/dag	☐ 2 tot 3 uren/dag
☐ 3 tot 4 uren/dag	☐ 3 tot 4 uren/dag
☐ 4 tot 5 uren/dag	☐ 4 tot 5 uren/dag
☐ 5 tot 6 uren/dag	☐ 5 tot 6 uren/dag
☐ 6 tot 7 uren/dag	☐ 6 tot 7 uren/dag
☐ Meer dan 7 uren/dag	☐ Meer dan 7 uren/dag

C2) Hoeveel tijd gebruiken uw gezinsleden gemiddeld een computer (denk aan tablets, internet op smartphone, laptop, computer,...) in hun VRIJE TIJD?

**Partner**	**Kinderen die thuis wonen**
☐ Niet van toepassing	☐ Niet van toepassing
☐ Gebruikt geen computer	☐ Gebruiken geen computer
☐ Slechts enkele keren per week	☐ Slechts enkele keren per week
☐ Minder dan 30 minuten/dag	☐ Minder dan 30 minuten/dag
☐ 30 tot 60 minuten/dag	☐ 30 tot 60 minuten/dag
☐ 1 tot 2 uur/dag	☐ 1 tot 2 uur/dag
☐ 2 tot 3 uur/dag	☐ 2 tot 3 uur/dag
☐ 3 tot 4 uur/dag	☐ 3 tot 4 uur/dag
☐ Meer dan 4 uur/dag	☐ Meer dan 4 uur/dag

C3) Hoe vaak gebruikt u een computer en dit op hetzelfde moment wanneer een gezinslid in dezelfde ruimte een andere computer gebruikt (in VRIJE TIJD)?

**Partner**	**Kinderen die thuis wonen**
☐ Niet van toepassing	☐ Niet van toepassing
☐ Nooit	☐ Nooit
☐ Zelden	☐ Zelden
☐ Soms	☐ Soms
☐ Vaak	☐ Vaak
☐ Heel vaak	☐ Heel vaak

3300

Er zijn verschillende situaties waarin u uw zittend gedrag tijdens computergebruik in de vrije tijd kan verminderen of (meer) kan onderbreken door even recht te staan. Duid voor elke stelling aan of u het zou zien zitten om volgende zaken uit te voeren.

**In welke mate zou u het zien zitten om:**	**Ik zie dit helemaal niet zitten**	**Ik zie dit eerder niet zitten**	**Neutraal**	**Ik zie dit eerder wel zitten**	**Ik zie dit zeker zitten**
C4) rechtstaand de computer te gebruiken.	☐	☐	☐	☐	☐
C5) een uur computergebruik te vervangen door lichte inspanningen (vb: kuisen, klusjes).	☐	☐	☐	☐	☐
C6) na 30 minuten de computer te hebben gebruikt gedurende enkele minuten recht te staan.	☐	☐	☐	☐	☐

Gelieve aan te duiden in welke mate u akkoord gaat met elk van de volgende stellingen over computergebruik in de vrije tijd.

	**Helemaal niet akkoord**	**Eerder niet akkoord**	**Neutraal**	**Eerder akkoord**	**Helemaal akkoord**	**Niet van toepassing**
C7) Ik vind het leuk om in de vrije tijd de computer te gebruiken.	☐	☐	☐	☐	☐	
C8) De computer gebruiken verhindert me belangrijkere zaken te doen.	☐	☐	☐	☐	☐	
C9) Ik vind het plezant gedurende een lange tijd de computer te gebruiken.	☐	☐	☐	☐	☐	
C10) De computer gebruiken is mijn manier om te ontspannen na een werk- of schooldag.	☐	☐	☐	☐	☐	
C11) Mijn gezinsleden vinden dat ik te veel de computer gebruik.	☐	☐	☐	☐	☐	☐
C12) Ik vind van mezelf dat ik te veel de computer gebruik.	☐	☐	☐	☐	☐	
C13) Ik zou het zien zitten om de computer gedurende enkele dagen in de week niet te gebruiken in de vrije tijd.	☐	☐	☐	☐	☐	
C14) Ik zou het zien zitten om de tijd die ik spendeer aan computergebruik te verminderen in de vrije tijd.	☐	☐	☐	☐	☐	
C15) Mijn gezinsleden moedigen me aan om minder de computer te gebruiken in de vrije tijd.	☐	☐	☐	☐	☐	☐
C16) Mijn vrienden moedigen me aan om minder de computer te gebruiken in de vrije tijd.	☐	☐	☐	☐	☐	

3300

D) GEMOTORISEERD TRANSPORT ALS WOON-WERKVERPLAATSING EN IN DE VRIJE TIJD

1) Personen die nog studeren noteren de tijd die ze nodig hebben om naar hun **school/opleiding** te gaan.
2) Onder gemotoriseerd transport verstaan we: auto, brommer, moto, trein, tram of bus.
3) Indien u **nooit** één van bovenstaande vervoersmiddelen gebruikt dan mag u verder naar pagina 12: " *E) Zitten op het werk* ".

VERPLAATSINGEN VAN & NAAR HET WERK

D1) Hoe lang zat u gemiddeld op één dag tijdens de laatste 7 dagen bij verplaatsingen van en naar uw werk/school? Denk aan het gebruik van een brommer, auto, bus, trein, tram of moto; reken fietsen niet mee.

- ☐ Niet
- ☐ 1 tot 15 minuten/dag
- ☐ 15 tot 30 minuten/dag
- ☐ 30 tot 45 minuten/dag
- ☐ 45 tot 60 minuten/dag
- ☐ 60 tot 90 minuten/dag
- ☐ 90 tot 120 minuten/dag
- ☐ 2 tot 2.5 uren/dag
- ☐ 2.5 tot 3 uren/dag
- ☐ 3 tot 4 uren/dag
- ☐ 4 tot 5 uren/dag
- ☐ 5 tot 6 uren/dag
- ☐ 6 tot 7 uren/dag
- ☐ Meer dan 7 uren per dag

VERPLAATSINGEN TIJDENS HET WERK

D2) Hoe lang zat u gemiddeld op één dag tijdens de laatste 7 dagen bij verplaatsingen als deel van uw werk (= tijdens werkuren)? Denk aan het gebruik van een brommer, auto, bus, trein, tram of moto; reken fietsen niet mee.

- ☐ Niet
- ☐ 1 tot 15 minuten/dag
- ☐ 15 tot 30 minuten/dag
- ☐ 30 tot 45 minuten/dag
- ☐ 45 tot 60 minuten/dag
- ☐ 60 tot 90 minuten/dag
- ☐ 90 tot 120 minuten/dag
- ☐ 2 tot 2.5 uren/dag
- ☐ 2.5 tot 3 uren/dag
- ☐ 3 tot 4 uren/dag
- ☐ 4 tot 5 uren/dag
- ☐ 5 tot 6 uren/dag
- ☐ 6 tot 7 uren/dag
- ☐ Meer dan 7 uren per dag

VERPLAATSINGEN IN DE VRIJE TIJD

D3) Hoe lang zat u gemiddeld op één dag tijdens de laatste 7 dagen bij verplaatsingen BUITEN uw werk/school. Hou hier dus rekening met uw transport in de vrije tijd (vb. naar winkel, vrienden opzoeken,...). Denk aan het gebruik van een auto, bus, trein, tram, moto of brommer; reken opnieuw fietsen niet mee.

Weekdag	Weekenddag
☐ Niet	☐ Niet
☐ 1 tot 15 minuten/dag	☐ 1 tot 15 minuten/dag
☐ 15 tot 30 minuten/dag	☐ 15 tot 30 minuten/dag
☐ 30 tot 45 minuten/dag	☐ 30 tot 45 minuten/dag
☐ 45 tot 60 minuten/dag	☐ 45 tot 60 minuten/dag
☐ 60 tot 90 minuten/dag	☐ 60 tot 90 minuten/dag
☐ 90 tot 120 minuten/dag	☐ 90 tot 120 minuten/dag
☐ 2 tot 2.5 uren/dag	☐ 2 tot 2.5 uren/dag
☐ 2.5 tot 3 uren/dag	☐ 2.5 tot 3 uren/dag
☐ 3 tot 4 uren/dag	☐ 3 tot 4 uren/dag
☐ 4 tot 5 uren/dag	☐ 4 tot 5 uren/dag
☐ 5 tot 6 uren/dag	☐ 5 tot 6 uren/dag
☐ 6 tot 7 uren/dag	☐ 6 tot 7 uren/dag
☐ Meer dan 7 uren per dag	☐ Meer dan 7 uren per dag

D4) Gelieve aan te geven hoeveel tijd u gemiddeld per dag spendeerde aan wandelen op een rustig tempo (bv: slenteren) als deel van uw transport (bv. naar werk wandelen, naar bus wandelen, naar winkel wandelen,...) tijdens de laatste 7 dagen.

Weekdag	Weekenddag
☐ Niet	☐ Niet
☐ 1 tot 15 minuten/dag	☐ 1 tot 15 minuten/dag
☐ 15 tot 30 minuten/dag	☐ 15 tot 30 minuten/dag
☐ 30 tot 45 minuten/dag	☐ 30 tot 45 minuten/dag
☐ 45 tot 60 minuten/dag	☐ 45 tot 60 minuten/dag
☐ 1 tot 2 uur/dag	☐ 1 tot 2 uur/dag
☐ Meer dan 2 uur/dag	☐ Meer dan 2 uur/dag

Voor onderstaande vragen wordt er onder transport steeds het gebruik van gemotoriseerde vervoersmiddelen verstaan (auto, brommer, moto, trein, tram of bus). Om de vragen te beantwoorden dient u rekening te houden met uw transport in de vrije tijd (naar winkel,...) EN uw transport naar werk/school.

Gelieve aan te duiden in welke mate u akkoord gaat met elk van onderstaande stellingen.

	Helemaal niet akkoord	Eerder niet akkoord	Neutraal	Eerder akkoord	Helemaal akkoord	Niet van toepassing
D5) Ik voel me lui als ik op mijn bestemming aankom na mijn gemotoriseerd transport.	☐	☐	☐	☐	☐	
D6) Ik vind het leuk om me met een gemotoriseerd voertuig te verplaatsen.	☐	☐	☐	☐	☐	
D7) Ik vind het leuk om als passagier tijdens gemotoriseerd transport aan bepaalde zaken te werken of uit te rusten.	☐	☐	☐	☐	☐	
D8) Ik vind van mezelf dat ik te veel gebruik maak van gemotoriseerd transport.	☐	☐	☐	☐	☐	
D9) Ik zou het zien zitten om spontaan een halte vroeger uit te stappen in tram/bus en het resterende eind te wandelen.	☐	☐	☐	☐	☐	
D10) Ik zou het zien zitten om me spontaan wat verder te parkeren met de auto en het resterende eind te wandelen.	☐	☐	☐	☐	☐	
D11) Ik zou het zien zitten om spontaan de fiets te nemen of te wandelen ook al heb ik een auto ter beschikking.	☐	☐	☐	☐	☐	
D12) Mijn gezinsleden moedigen me aan om me op een (meer) actieve manier (fietsen, wandelen) te verplaatsen.	☐	☐	☐	☐	☐	☐
D13) Mijn vrienden moedigen me aan om me op een (meer) actieve manier (fietsen, wandelen) te verplaatsen.	☐	☐	☐	☐	☐	
D14) Mijn gezinsleden vinden dat ik te veel gebruik maak van gemotoriseerd transport.	☐	☐	☐	☐	☐	☐

3300

D15) Wat zijn voor u de voornaamste redenen om u niet actief (wandelen of fietsen) te verplaatsen? Meerdere antwoorden zijn mogelijk.

- ☐ De omgeving is niet veilig om te wandelen of te fietsen
- ☐ Actief transport (wandelen of fietsen) is niet aantrekkelijk (geen groene omgeving in de buurt, saai,...)
- ☐ Actief transport is vermoeiend (zweten, bagage dragen,...)
- ☐ De voorzieningen voor gemotoriseerd transport zijn goed uitgerust (bushaltes dicht bij de deur,...)
- ☐ Tijdsdruk
- ☐ Te grote afstand
- ☐ Weersomstandigheden
- ☐ Het combineren van verschillende taken (naar bakker, kinderen ergens afzetten,....)
- ☐ Financiële redenen (terugbetaling kilometers van trein, tram, bus of auto via het werk)
- ☐ Andere (noteer) ____________________

D16) Hoe verplaatsen uw gezinsleden zich meestal... (slechts 1 antwoord mogelijk per rij)

a) naar het werk of school?

	Niet van toepassing	Gemotoriseerd transport	Actief transport (wandelen/fietsen)
Partner	☐	☐	☐
Kinderen die thuis wonen	☐	☐	☐

b) in de vrije tijd?

	Niet van toepassing	Gemotoriseerd transport	Actief transport (wandelen/fietsen)
Partner	☐	☐	☐
Kinderen die thuis wonen	☐	☐	☐

Er zijn verschillende situaties waarin u uw zittend gedrag tijdens gemotoriseerd transport kan verminderen of (meer) kan onderbreken door even recht te staan. Duid voor elke stelling aan of u het zou zien zitten om volgende zaken uit te voeren.

In welke mate zou u het zien zitten om:	Ik zie dit helemaal niet zitten	Ik zie dit eerder niet zitten	Neutraal	Ik zie dit eerder wel zitten	Ik zie dit zeker zitten
D17) spontaan recht te staan op de bus, trein of tram in plaats van neer te zitten.	☐	☐	☐	☐	☐
D18) na 30 minuten zitten in bus, trein of tram even kort te gaan rechtstaan.	☐	☐	☐	☐	☐

Geef over onderstaande beweringen uw mening i.v.m. de omgeving waarin u zich begeeft tijdens uw gemotoriseerd transport. Beoordeel hier de omgeving op ongeveer 10 à 15 minuten wandelen of op 1 km van uw woning.

	Helemaal niet akkoord	Eerder niet akkoord	Neutraal	Eerder akkoord	Helemaal akkoord
D19) Als ik me buitenshuis begeef naar openbare plaatsen (winkels/station/...) kan ik gemakkelijk parkeren.	☐	☐	☐	☐	☐
D20) Via informatieborden langs de weg en/of radio krijg ik veel info over zittende activiteiten zoals cinema of sportwedstrijden.	☐	☐	☐	☐	☐

3300

D21) Hoe vaak verplaatst u zich SAMEN met uw gezinsleden met gemotoriseerd transport? Doe dit apart voor transport van/naar het werk of school en transport in de vrije tijd (afzonderlijk voor weekdag en weekenddag).

Van/naar het werk of school	
**Partner**	**Kinderen die thuis wonen**
☐ Niet van toepassing	☐ Niet van toepassing
☐ Nooit	☐ Nooit
☐ Zelden	☐ Zelden
☐ Soms	☐ Soms
☐ Vaak	☐ Vaak
☐ Heel vaak	☐ Heel vaak

Vrije tijd			
**Partner**		**Kinderen die thuis wonen**	
**Weekdag**	**Weekenddag**	**Weekdag**	**Weekenddag**
☐ Niet van toepassing	☐ Niet van toepassing	☐ Niet van toepassing	☐ Niet van toepassing
☐ Nooit	☐ Nooit	☐ Nooit	☐ Nooit
☐ Zelden	☐ Zelden	☐ Zelden	☐ Zelden
☐ Soms	☐ Soms	☐ Soms	☐ Soms
☐ Vaak	☐ Vaak	☐ Vaak	☐ Vaak
☐ Heel vaak	☐ Heel vaak	☐ Heel vaak	☐ Heel vaak

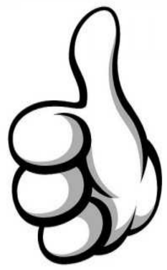

U bent al voorbij de helft van de vragen

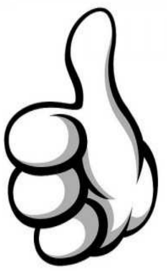

3300

E) ZITTEN OP HET WERK

Indien u momenteel geen werk hebt (bv: studenten, werkzoekenden,...), dan mag u de vragen in dit deel overslaan en verder gaan naar pagina 15: "*F) Huishoudelijke bezigheden en telefoneren*".

E1) Hoeveel tijd bracht u per dag gemiddeld al zittend door op het werk tijdens de laatste 7 dagen?

Minder dan 2 uur per dag	2 tot 3 uur/dag	3 tot 4 uur/dag	4 tot 5 uur/dag	5 tot 6 uur/dag	6 tot 7uur/dag	7 tot 8uur/dag	Meer dan 8 uur per dag
☐	☐	☐	☐	☐	☐	☐	☐

De volgende stellingen zijn verschillende meningen over uw zittend gedrag op het werk. Gelieve aan te duiden in welke mate u akkoord gaat met elk van de onderstaande stellingen.

	Helemaal niet akkoord	Eerder niet akkoord	Neutraal	Eerder akkoord	Helemaal akkoord	Niet van toepassing
E2) Mijn aandacht neemt af wanneer ik gedurende een lange tijd neerzit.	☐	☐	☐	☐	☐	
E3) Ik vind het plezant om langdurig neer te zitten tijdens mijn werkuren.	☐	☐	☐	☐	☐	
E4) Zitten op het werk is een ideale gelegenheid om uit te rusten.	☐	☐	☐	☐	☐	
E5) Mijn collega's vinden dat ik te veel zit.	☐	☐	☐	☐	☐	☐
E6) Ik vind van mezelf dat ik te veel zit op mijn werk.	☐	☐	☐	☐	☐	
E7) Ik zou het zien zitten om bepaalde taken (telefoneren,...) rechtstaand uit te voeren.	☐	☐	☐	☐	☐	
E8) Ik zou het zien zitten om na lang zitten even recht te staan.	☐	☐	☐	☐	☐	
E9)Ik zou het zien zitten om meer recht te staan tijdens de pauzes.	☐	☐	☐	☐	☐	
E10) Mijn collega's moedigen me aan om minder te zitten.	☐	☐	☐	☐	☐	☐
E11) Mijn collega's op mijn bureau en/of afdeling staan regelmatig recht tijdens de werkuren en/of pauzes.	☐	☐	☐	☐	☐	☐

E12) Indien u een vraag heeft voor een collega op een ander bureau, wat doet u dan meestal? (gelieve slechts 1 antwoord te geven)

- ☐ Ik ga persoonlijk naar mijn collega
- ☐ Ik stuur een E-mail
- ☐ Ik telefoneer naar mijn collega
- ☐ Ik gebruik een intern chatsysteem op het werk
- ☐ Niet van toepassing (bv. zelfstandig, niet werken op een bureau,...)

3300

E13) Hoeveel personen zitten er op uw bureau, uzelf ook meegerekend?

- ☐ Alleen mezelf
- ☐ 2 personen
- ☐ 3 personen
- ☐ 4 personen
- ☐ Meer dan 4 personen

Beantwoord volgende vragen omtrent uw werk.

	**Ja**	**Neen**
E14) Op mijn werk zijn er fietsen ter beschikking gesteld door mijn werkgever	☐	☐
E15) Op mijn werk kan ik genieten van een terugbetaling van openbaar vervoer en/of fietsvergoeding	☐	☐
E16) De (bureau)stoelen zijn comfortabel.	☐	☐
E17) Er is een ruime en/of makkelijke parking aanwezig.	☐	☐
E18) Er is een fietsenstalling aanwezig.	☐	☐
E19) Er zijn standing desks aanwezig (verhoogde bureaus waaraan rechtstaand gewerkt kan worden).	☐	☐

Er zijn verschillende situaties waarin u uw zittend gedrag op uw werk kan verminderen of (meer) kan onderbreken door even recht te staan. Duid voor elke stelling aan of u het zou zien zitten om volgende zaken uit te voeren.

**In welke mate zou u het zien zitten om:**	**Ik zie dit helemaal niet zitten**	**Ik zie dit eerder niet zitten**	**Neutraal**	**Ik zie dit eerder wel zitten**	**Ik zie dit zeker zitten**
E20) rechtstaand te werken aan uw bureau.	☐	☐	☐	☐	☐
E21) rechtstaand te telefoneren.	☐	☐	☐	☐	☐
E22) in plaats van te telefoneren naar een collega, er zelf naar toe te gaan.	☐	☐	☐	☐	☐
E23) na 30 minuten zitten enkele minuten recht te staan.	☐	☐	☐	☐	☐
E24) toestellen te verplaatsen zodat u moet recht staan om deze te bedienen (printer, telefoon,...).	☐	☐	☐	☐	☐

Duid aan in welke mate u akkoord gaat met volgende stellingen.

	**Helemaal niet akkoord**	**Eerder niet akkoord**	**Neutraal**	**Eerder akkoord**	**Helemaal akkoord**	**Niet van toepassing**
**Zittende activiteiten onderbreken door recht te staan op mijn werk zie ik als:** E25) storend voor collega's	☐	☐	☐	☐	☐	☐
E26) belachelijk	☐	☐	☐	☐	☐	
E27) goed voor mijn gezondheid	☐	☐	☐	☐	☐	
**Ik ben er zeker van dat ik (meer) kan rechtstaan als onderbreking tijdens mijn werk, ook al:** E28) doen mijn collega's dit niet	☐	☐	☐	☐	☐	☐
E29) word ik niet gesteund door mijn collega's	☐	☐	☐	☐	☐	☐

3300

Geef aan hoe u het liefst onderstaande taken op het werk zou uitvoeren.

	Rechtstaand	Zittend
E30) Telefoneren	☐	☐
E31) Kort vergaderen	☐	☐
E32) Bureauwerk	☐	☐
E33) Koffie pauzes	☐	☐
E34) Middagpauzes	☐	☐

E35) Gelieve aan te geven hoeveel tijd u gemiddeld per dag spendeerde aan de onderstaande activiteiten/situaties op het werk tijdens de laatste 7 dagen.
Rechtstaand werken= rechtstaand vergaderen/telefoneren/brainstormen/computer gebruiken/...

Rechtstaand werken	Wandelen aan rustig tempo (bv slenteren)
☐ Niet	☐ Niet
☐ 1 tot 15 minuten/dag	☐ 1 tot 15 minuten/dag
☐ 15 tot 30 minuten/dag	☐ 15 tot 30 minuten/dag
☐ 30 tot 45 minuten/dag	☐ 30 tot 45 minuten/dag
☐ 45 tot 60 minuten/dag	☐ 45 tot 60 minuten/dag
☐ 1 tot 2 uren/dag	☐ 1 tot 2 uren/dag
☐ 2 tot 3 uren/dag	☐ Meer dan dan 2 uur/dag
☐ 3 tot 4 uren/dag	
☐ 4 tot 5 uren/dag	
☐ 5 tot 6 uren/dag	
☐ 6 tot 7 uren/dag	
☐ Meer dan 7 uren/dag	

Beantwoord onderstaande vragen omtrent uw werk.

	Zeker akkoord	Akkoord	Niet akkoord	Zeker niet akkoord	Niet van toepassing
E36) Ik kan verschillende collega's zien zitten of staan aan hun bureau wanneer ik aan mijn bureau zit (reken hier geen bureaus mee met gesloten deuren).	☐	☐	☐	☐	☐
E37) Ik kom regelmatig collega's tegen wanneer ik door de gangen wandel in mijn gebouw.	☐	☐	☐	☐	☐
E38) Ik zie regelmatig collega's die aan het wandelen zijn in ons gebouw.	☐	☐	☐	☐	☐
E39) Ik zie regelmatig collega's die rechtstaan en aan het praten zijn in ons gebouw.	☐	☐	☐	☐	☐

3300

F) HUISHOUDELIJKE BEZIGHEDEN EN TELEFONEREN

F1) Hoeveel tijd bracht u gemiddeld per dag al zittend door tijdens huishoudelijke taken en thuis telefoneren tijdens de laatste 7 dagen? Mogelijke huishoudelijke taken zijn: koken, strijken, herstellingen uitvoeren,...

Zittend huishoudelijke taken doen (koken, herstellingen,...) & telefoneren	
Weekdag	Weekenddag
☐ Niet	☐ Niet
☐ 1 tot 15 minuten/dag	☐ 1 tot 15 minuten/dag
☐ 15 tot 30 minuten/dag	☐ 15 tot 30 minuten/dag
☐ 30 tot 60 minuten/dag	☐ 30 tot 60 minuten/dag
☐ 1 tot 2 uren/dag	☐ 1 tot 2 uren/dag
☐ 2 tot 3 uren/dag	☐ 2 tot 3 uren/dag
☐ 3 tot 4 uren/dag	☐ 3 tot 4 uren/dag
☐ 4 tot 5 uren/dag	☐ 4 tot 5 uren/dag
☐ 5 tot 6 uren/dag	☐ 5 tot 6 uren/dag
☐ 6 tot 7 uren/dag	☐ 6 tot 7 uren/dag
☐ Meer dan 7 uren/dag	☐ Meer dan 7 uren/dag

De volgende stellingen zijn verschillende meningen over zittende activiteiten in het huishouden en thuis telefoneren. Gelieve aan te duiden in welke mate u akkoord gaat met elk van de onderstaande stellingen.

	Helemaal niet akkoord	Eerder niet akkoord	Neutraal	Eerder akkoord	Helemaal akkoord	Niet van toepassing
F2) Ik vind het leuk om te zitten tijdens huishoudelijke taken.	☐	☐	☐	☐	☐	
F3) Wanneer ik zittend werk in het huishouden kan ik sneller werken.	☐	☐	☐	☐	☐	
F4) Ik zou liever meer rechtstaan tijdens huishoudelijke taken.	☐	☐	☐	☐	☐	
F5) Ik vind van mezelf dat ik meer kan rechtstaan tijdens huishoudelijke taken.	☐	☐	☐	☐	☐	
F6) Ik zou het zien zitten om rechtstaand te telefoneren.	☐	☐	☐	☐	☐	
F7) Ik zou het zien zitten om taken in de keuken rechtstaand uit te voeren.	☐	☐	☐	☐	☐	
F8) Mijn gezinsleden moedigen me aan om minder te zitten tijdens huishoudelijke taken.	☐	☐	☐	☐	☐	☐
F9) Huishoudelijke taken worden door mijn kinderen meestal zittend uitgevoerd.	☐	☐	☐	☐	☐	☐
F10) Huishoudelijke taken worden door mijn partner meestal zittend uitgevoerd.	☐	☐	☐	☐	☐	☐

3300

Geef aan hoe u het liefst onderstaande huishoudelijke taken zou uitvoeren.

	Rechtstaand	Zittend
F11) Telefoneren	☐	☐
F12) Aardappelen schillen/groenten snijden	☐	☐
F13) Strijken	☐	☐
F14) Was opvouwen	☐	☐
F15) Tanden poetsen	☐	☐
F16) Herstellingen uitvoeren	☐	☐

Er zijn verschillende situaties waarin u uw zittend gedrag in het huishouden kan verminderen of (meer) kan onderbreken door even recht te staan. Duid voor elke stelling aan of u het zou zien zitten om volgende zaken uit te voeren.

In welke mate zou u het zien zitten om:	Ik zie dit helemaal niet zitten	Ik zie dit eerder niet zitten	Neutraal	Ik zie dit eerder wel zitten	Ik zie dit zeker zitten
F17) meer huishoudelijke taken rechtstaand uit te voeren.	☐	☐	☐	☐	☐
F18) toestellen te verplaatsen zodat u moet rechtstaan om deze te kunnen bedienen (printer, afstandsbediening tv,...).	☐	☐	☐	☐	☐

Duid aan in welke mate u akkoord gaat met volgende stellingen.

	Helemaal niet akkoord	Eerder niet akkoord	Neutraal	Eerder akkoord	Helemaal akkoord	Niet van toepassing
**Ik ben er zeker van dat ik mijn zittende activiteiten thuis meer kan onderbreken, ook al:** F19) doen mijn gezinsleden dit niet.	☐	☐	☐	☐	☐	☐
F20) steunen/begrijpen mijn gezinsleden mij niet.	☐	☐	☐	☐	☐	☐

Hoe bekijkt u de nabije omgeving van uw woonplaats (binnen een straal van 1 km of op 10-15 minuten wandelen)?

	Helemaal niet akkoord	Eerder niet akkoord	Neutraal	Eerder akkoord	Helemaal akkoord
F21) De meeste woningen in mijn buurt zijn alleenstaande woningen (open bebouwing).	☐	☐	☐	☐	☐
F22) Er zijn veel winkels op wandelafstand van mijn huis.	☐	☐	☐	☐	☐
F23) Er is een halte van het openbaar vervoer (bus en/of tram) op wandelafstand van mijn huis.	☐	☐	☐	☐	☐
F24) Er is een park op wandelafstand van mijn huis.	☐	☐	☐	☐	☐
F25) Wandelen is gevaarlijk omwille van het verkeer in mijn buurt.	☐	☐	☐	☐	☐
F26) Wandelen in mijn buurt is gevaarlijk omwille van de hoge kans op misdaad.	☐	☐	☐	☐	☐
F27) In mijn buurt staan er bomen langs de straten.	☐	☐	☐	☐	☐
F28) Ik vind mijn buurt een goede plaats om in te leven.	☐	☐	☐	☐	☐
F29) De omgeving nodigt niet uit om te bewegen (veel heuvels, geen voetpad,...).	☐	☐	☐	☐	☐

Gelieve het aantal niet draagbare toestellen te noteren die aanwezig zijn bij u thuis EN waarvan u ZELF ook gebruik maakt.

	Geen	1	2	3	4	5	Meer dan 5
F30) Televisies MET betaalzenders	☐	☐	☐	☐	☐	☐	☐
F31) Televisies ZONDER betaalzenders	☐	☐	☐	☐	☐	☐	☐
F32) Videospelers/Dvd-spelers	☐	☐	☐	☐	☐	☐	☐
F33) Muziekinstallaties (radio, CD-spelers, stereo,...)	☐	☐	☐	☐	☐	☐	☐
F34) Computer (vaste)	☐	☐	☐	☐	☐	☐	☐
F35) Spelconsoles op tv (Playstation, Xbox,...)	☐	☐	☐	☐	☐	☐	☐
F36) Telefoontoestellen (vaste lijn)	☐	☐	☐	☐	☐	☐	☐

Hoeveel van bovenstaande toestellen bevinden zich in uw slaapkamer?

	Geen	1	2	3	4	5	Meer dan 5
F37) Televisies MET betaalzenders	☐	☐	☐	☐	☐	☐	☐
F38) Televisies ZONDER betaalzenders	☐	☐	☐	☐	☐	☐	☐
F39) Videospelers/Dvd-spelers	☐	☐	☐	☐	☐	☐	☐
F40) Muziekinstallaties (radio, CD-spelers, stereo,...)	☐	☐	☐	☐	☐	☐	☐
F41) Computer (vaste)	☐	☐	☐	☐	☐	☐	☐
F42) Spelconsoles op tv (Playstation, Xbox,...)	☐	☐	☐	☐	☐	☐	☐
F43) Telefoontoestellen (vaste lijn)	☐	☐	☐	☐	☐	☐	☐

Gelieve het aantal draagbare toestellen te noteren die aanwezig zijn bij u thuis EN waarvan u ZELF ook gebruik maakt.

	Geen	1	2	3	4	5	Meer dan 5
F44) Laptop	☐	☐	☐	☐	☐	☐	☐
F45) GSM (geen smartphone)	☐	☐	☐	☐	☐	☐	☐
F46) Smartphone	☐	☐	☐	☐	☐	☐	☐
F47) Muziekspelers (zoals iPod, MP3,...)	☐	☐	☐	☐	☐	☐	☐
F48) Spelconsoles (zoals PSP, game boy,...)	☐	☐	☐	☐	☐	☐	☐
F49) Tablet (iPad, Samsung Galaxy Tab,...)	☐	☐	☐	☐	☐	☐	☐

Hoe beoordeelt u volgende beweringen omtrent de voorzieningen bij u thuis?

	Helemaal niet akkoord	Eerder niet akkoord	Neutraal	Eerder akkoord	Helemaal akkoord	Niet van toepassing
F50) De afstandsbediening van de tv ligt steeds binnen handbereik wanneer ik deze nodig heb.	☐	☐	☐	☐	☐	☐
F51) De zetels bij ons thuis zijn comfortabel om gedurende een lange tijd in te zitten.	☐	☐	☐	☐	☐	☐

Gelieve aan te geven hoeveel tijd u gemiddeld per dag spendeerde aan de onderstaande rechtstaande activiteiten/situaties in het huishouden tijdens de laatste 7 dagen.

Voorbeelden van licht intense activiteiten in het huishouden (RECHTstaand uitgevoerd)	
Afstoffen	Aankleden/uitkleden
Koken	Spelen met kinderen (rustig)
Strijken	Eten opdienen
Afwassen, tafel opruimen	Dieren eten geven
Planten water geven	Persoonlijke hygiëne (scheren, make-up,...)
Rechtstaand telefoneren	De was doen, kleren ophangen voor te drogen,...

		Niet	1 tot 15 minuten/dag	15 tot 30 minuten/dag	30 tot 45 minuten/dag	45 tot 60 minuten/dag	1 tot 2 uur/dag	Meer dan 2 uur/dag
**HUISHOUDEN (RECHTSTAAND UITGEVOERD)**								
**F52) Kuisen** (afstoffen, strijken,....)	weekdag	☐	☐	☐	☐	☐	☐	☐
	weekenddag	☐	☐	☐	☐	☐	☐	☐
**F53) Keuken** (koken, voorbereiding,...)	weekdag	☐	☐	☐	☐	☐	☐	☐
	weekenddag	☐	☐	☐	☐	☐	☐	☐
**F54) Kinderen** (rustig samen spelen,...)	weekdag	☐	☐	☐	☐	☐	☐	☐
	weekenddag	☐	☐	☐	☐	☐	☐	☐
**TUIN (RECHTSTAAND UITGEVOERD)**								
F55) Planten water geven, dieren eten geven,...	weekdag	☐	☐	☐	☐	☐	☐	☐
	weekenddag	☐	☐	☐	☐	☐	☐	☐
**VERZORGING (RECHTSTAAND UITGEVOERD)**								
**F56) Verzorging** (wassen, scheren, make-up, tanden poetsen,...)	weekdag	☐	☐	☐	☐	☐	☐	☐
	weekenddag	☐	☐	☐	☐	☐	☐	☐
**VRIJE TIJD/HOBBY'S (RECHTSTAAND UITGEVOERD)**								
F57) Winkelen, staand tv-kijken, staand muziek luisteren,...	weekdag	☐	☐	☐	☐	☐	☐	☐
	weekenddag	☐	☐	☐	☐	☐	☐	☐
**WANDELEN OP RUSTIG TEMPO (bv. slenteren)**								
F58) Wandelen op rustig tempo (bv. slenteren) in huis	weekdag	☐	☐	☐	☐	☐	☐	☐
	weekenddag	☐	☐	☐	☐	☐	☐	☐

3300

G) ANDERE ZITTENDE/LIGGENDE ACTIVITEITEN

Hoe lang zat u gemiddeld op één dag tijdens de laatste 7 dagen voor de volgende zittende activiteiten? Onder **zittende activiteiten** worden ook **liggende activiteiten** verstaan. Elke periode van zitten of liggen mag slechts **1 keer in rekening** worden gebracht. Indien u bv. 1 uur in de zetel zat om een boek te lezen terwijl u naar muziek luisterde, reken deze tijd dan als 1 uur lezen indien dit uw voornaamste bezigheid was. Reken deze tijd dan niet opnieuw als 1 uur naar muziek luisteren!

		Niet	1 tot 15 minuten per dag	15 tot 30 minuten per dag	30 tot 60 minuten per dag	1 tot 2 uur per dag	2 tot 3 uur per dag	3 tot 4 uur per dag	4 tot 5 uur per dag	5 tot 6 uur per dag	6 tot 7 uur per dag	Meer dan 7 uur per dag
**G1) Zittend lezen** (boek, tijdschrift, krant,...)	weekdag	☐	☐	☐	☐	☐	☐	☐	☐	☐	☐	☐
	weekenddag	☐	☐	☐	☐	☐	☐	☐	☐	☐	☐	☐
**G2) Zittend zorgen** voor kinderen, familieleden,....	weekdag	☐	☐	☐	☐	☐	☐	☐	☐	☐	☐	☐
	weekenddag	☐	☐	☐	☐	☐	☐	☐	☐	☐	☐	☐
**G3) Zitten bij hobby's** (tekenen, kaarten,...)	weekdag	☐	☐	☐	☐	☐	☐	☐	☐	☐	☐	☐
	weekenddag	☐	☐	☐	☐	☐	☐	☐	☐	☐	☐	☐
**G4) Zitten bij sociaal contact** (vrienden bezoeken, café, cinema,....	weekdag	☐	☐	☐	☐	☐	☐	☐	☐	☐	☐	☐
	weekenddag	☐	☐	☐	☐	☐	☐	☐	☐	☐	☐	☐
**G5) Zittend naar muziek luisteren** (radio, CD, iPod,...)	weekdag	☐	☐	☐	☐	☐	☐	☐	☐	☐	☐	☐
	weekenddag	☐	☐	☐	☐	☐	☐	☐	☐	☐	☐	☐
**G6) Maaltijden** (ontbijt,...)	weekdag	☐	☐	☐	☐	☐	☐	☐	☐	☐	☐	☐
	weekenddag	☐	☐	☐	☐	☐	☐	☐	☐	☐	☐	☐

Hoe vaak doet u onderstaande situaties op HETZELFDE moment.

	Nooit	Zelden	Soms	Vaak	Heel vaak	Niet van toepassing
G7) tv kijken - GSM gebruiken	☐	☐	☐	☐	☐	☐
G8) tv kijken - computer/tablet	☐	☐	☐	☐	☐	☐
G9) computer - GSM gebruiken	☐	☐	☐	☐	☐	☐
G10) computer - muziek luisteren	☐	☐	☐	☐	☐	☐
G11) GSM - praten met vrienden/familie	☐	☐	☐	☐	☐	☐
G12) GSM - muziek luisteren	☐	☐	☐	☐	☐	☐

H) Gezondheid

Heeft u gedurende de voorbije maand vaak last gehad van een neerslachtig, depressief of hopeloos gevoel?

☐ Ja ☐ Neen

Heeft u gedurende de laatste maand minder plezier of interesse in wat u doet?

☐ Ja ☐ Neen

I) Fysieke activiteit

Beantwoord onderstaande vragen rekening houdend met volgende activiteiten: activiteiten op het werk, huishouden, werken in tuin, zich verplaatsen van en naar verschillende plaatsen, activiteiten in vrije tijd als ontspanning en sport.

Op hoeveel dagen heeft u tenminste 10 minuten per keer gewandeld als u denkt aan de laatste 7 dagen? Denk hierbij aan wandelen op het werk en thuis, wandelen om van de ene naar de andere plaats te komen, en al het andere wandelen dat u deed tijdens recreatie, sport of vrijetijdsbesteding.

☐ Geen wandelen

___ dag(en) per week

Op de dagen dat u ten minste 10 minuten per keer wandelde, hoeveel tijd heeft u daar dan gewoonlijk aan besteed?

___ uur/uren ___ minuten per dag

Op hoeveel dagen heeft u matige fysieke activiteiten verricht, zoals het dragen van lichte lasten, fietsen in een normaal tempo of dubbeltennis als u denkt aan de laatste 7 dagen? Laat wandelen hier buiten beschouwing. Matig intensieve lichamelijke fysieke activiteit laat u iets sneller ademen dan normaal. Denkt u weer alleen aan activiteiten die u ten minste 10 minuten per keer heeft verricht.

☐ Ik heb geen matig intensieve lichamelijke fysieke activiteiten verricht

___ dag(en) per week

Op de dagen dat u matig intensief lichamelijk fysiek actief was, hoeveel tijd heeft u daar dan gewoonlijk aan besteed?

___ uur/uren ___ minuten per dag

Op hoeveel dagen heeft u zware lichamelijke fysieke activiteiten verricht zoals zware lasten tillen, spitten, sporten, aerobics, voetballen of wielrennen als u denkt aan de laatste 7 dagen? Denk alleen aan de activiteiten die u ten minste 10 minuten per keer heeft verricht. Zware lichamelijke fysieke activiteiten zijn activiteiten die veel lichamelijke inspanning kosten en voor een veel snellere ademhaling zorgen.

☐ Ik heb geen zware lichamelijke fysieke activiteiten verricht

___ dag(en) per week

Op de dagen dat u zwaar lichamelijk fysiek actief was, hoeveel tijd heeft u daar dan gewoonlijk aan besteed?

___ uur/uren ___ minuten per dag

J) Informatief gedeelte

J1) Wat is uw moedertaal? (gelieve 1 antwoord te geven)

☐ Nederlands
☐ Frans
☐ Turks
☐ Arabisch
☐ Engels
☐ Andere (noteer) ____________________

J2) Wat is het hoogst behaalde diploma of getuigschrift dat u en eventueel uw partner behaald heeft?

UZELF	PARTNER
☐ Lager onderwijs<br>☐ Beroeps secundair onderwijs (BSO)<br>☐ Technisch secundair onderwijs (TSO)<br>☐ Algemeen secundair onderwijs (ASO)<br>☐ Hoger onderwijs, niet-universitair<br>☐ Universitair onderwijs	☐ Niet van toepassing / weet ik niet<br>☐ Lager onderwijs<br>☐ Beroeps secundair onderwijs (BSO)<br>☐ Technisch secundair onderwijs (TSO)<br>☐ Algemeen secundair onderwijs (ASO)<br>☐ Hoger onderwijs, niet-universitair<br>☐ Universitair onderwijs

3300

J3) Momenteel... (gelieve slechts 1 antwoord te geven)

- ☐ werk ik voltijds
- ☐ werk ik halftijds of parttime
- ☐ doe ik het huishouden
- ☐ ben ik werkloos/werkzoekende
- ☐ heb ik een loopbaanonderbreking
- ☐ ben ik gepensioneerd
- ☐ ben ik student

J4) Indien u werkt, in welke sector werkt u?

- ☐ Niet van toepassing
- ☐ Onderwijs
- ☐ Bediende
- ☐ Kaderpersoneel
- ☐ Zelfstandige (geen vrij beroep)
- ☐ Vrij beroep
- ☐ Arbeider

J5) Wat is uw lichaamslengte (cm)? ___ ___ ___ cm

J6) Wat is uw lichaamsgewicht (kg)? ___ ___ ___ kg

J7) Hoeveel motorvoertuigen (wagens, moto's, brommers,...) die nog werken, zijn er aanwezig in uw gezin? Gelieve de voertuigen waarvan u geen gebruik maakt OOK mee te rekenen.

___ ___ (Noteer hier het aantal)

Bent u bereid om gedurende 1 week twee kleine bewegingsmetertjes te dragen, zodat we uw mate van beweging in kaart kunnen brengen? Deze bewegingsmeters (gedragen op heup en bovenbeen) hinderen u niet met uw dagelijkse bezigheden. Indien u ons hiervoor de toestemming geeft, nemen we zo snel mogelijk contact met u op. Indien u na het dragen van de bewegingsmetertjes graag uw persoonlijke resultaten krijgt, sturen we u deze graag door!

- ☐ Ja
- ☐ Neen

Indien ja, op welk telefoonnummer kunnen we u bereiken?

BEDANKT!!!
