# Supplementary material for: Reliability and validity of three questionnaires measuring context-specific sedentary behaviour and associated correlates in adolescents, adults and older adults
Source: Int J Behav Nutr Phys Act. 2015 Sep 17;12:117. doi: 10.1186/s12966-015-0277-2 (PMC4574538; doi:10.1186/s12966-015-0277-2)
Supplement: Additional file 3: — Questionnaire older adults (Dutch). (PDF 248 kb) [file 12966_2015_277_MOESM3_ESM.pdf]

6171

Vragenlijst voor 65-plussers uit Sint-Niklaas

A) Instructies voor de INTERVIEWER:
- Gelieve slechts **1 antwoord** aan te duiden (indien meerdere antwoorden mogelijk zijn, zal dit bij de vraag vermeld staan).
- Gelieve de vragenlijst in te vullen met een **blauwe of zwarte balpen**.
- Indien er een antwoord voluit geschreven moet worden, gelieve dit in **drukletters** te doen. Bij andere vragen moet u een kruis ("X") in het vakje bij het juiste antwoord plaatsen. Indien de deelnemer een **antwoord wil veranderen**, laat de "X" staan bij het foute antwoord en maak het vakje met het juiste antwoord volledig zwart.
- Vragenlijst correct invullen en vragen goed lezen!
- De antwoordmogelijkheid **"niet van toepassing"** wil zeggen dat deze vraag voor de persoon niet mogelijk is om te beantwoorden (bv. geen partner). Deze antwoordmogelijkheid staat meestal niet op de versie die de persoon krijgt om mee te lezen, maar wel op de versie van de interviewer. Hiermee willen we uitsluiten dat de persoon onnodig deze antwoordmogelijkheid geeft (bv. wanneer hij/zij eigenlijk wel kan antwoorden). Hierdoor is het NIET de bedoeling dat u bij het overlopen van de antwoordmogelijkheden "niet van toepassing" vermeld. Het is de bedoeling wanneer de persoon geen partner heeft, u voor deze persoon (zonder de vraag te stellen) "niet van toepassing" aanduidt (zie **(*)** pagina 2).
- Bij bepaalde vragen moet de deelnemer **aangeven hoeveel tijd** hij/zij spendeerde aan bepaalde bezigheden. De antwoordmogelijkheden zijn zo opgebouwd: .... - 1 tot 2 uur/dag - 2 tot 3 uur/dag - .....
Indien hij/zij aangeeft dit 2 uur te doen, dan dient u 2 tot 3 uur/dag aan te duiden (want antwoordmogelijkheden zijn tot... en **niet** tot en met!). Reken bij de antwoordmogelijkheid "meer dan 7 uur" ook 7 uur bij.
- Lees bij elke vraag de bijhorende **[tips]** en **[verduidelijkingen]**!
- Geef bij de **start** van het interview enkel **pagina's 1 & 2!**
- Bij sommige vragen dient er een **antwoordkaart** te worden gegeven aan de deelnemer. Er zijn verschillende antwoordkaarten, maar de **nummer** staat steeds genoteerd bij de bijhorende vraag.
- Wanneer deelnemer bij een vraag moet zeggen hoeveel tijd zij/hij hier gemiddeld aan spendeerde per dag tijdens de laatste 7 dagen en deelnemer kan niet antwoorden omwille van een te grote variatie van dag tot dag, vraag dan "Hoeveel tijd spendeerde u in totaal gedurende de laatste 7 dagen aan .... **Deel** dit totaal dan zelf door 5 of 2!
- Laat de deelnemer bij deelname onderaan deze pagina zijn/haar **handtekening** (met datum) plaatsen. Overloop hiervoor eerst de informatiebrief, zodat de deelnemer weet waarover dit onderzoek gaat.

B) Instructies meegeven aan de DEELNEMER:
- Aangeven hoe ze zich **persoonlijk** voelen bij elke stelling.
- Meegeven dat de antwoorden **vertrouwelijk** worden verwerkt (er worden geen namen vrijgegeven)!
- Er zijn **GEEN** foute antwoorden!

Ik, ... **(naam en voornaam)** ga akkoord, na het doornemen van de informatiebrief met de onderzoeker, om deel te nemen aan de studie over zitten en licht intense activiteiten.

Handtekening (met datum) voor akkoord:

6171

A) ALGEMENE INFORMATIE

Enkele algemene vragen... (geef duidelijk mee dat antwoorden anoniem worden verwerkt):

- Initialen van voornaam en achternaam: ☐☐☐☐☐☐

- Geboortedatum (dag/maand/jaar): ☐☐ / ☐☐ / ☐☐☐☐

- Datum van vandaag (dag/maand/jaar): ☐☐ / ☐☐ / ☐☐☐☐

- E-mail adres (indien mogelijk):

____

- Telefoonnummer of GSM-nummer (wordt enkel gebruikt bij onduidelijke gegevens)

____

A1) Geslacht?

☐ Man ☐ Vrouw

A2) Hoe kunt u uw huidige gezinssituatie het best omschrijven? [overloop onderstaande mogelijkheden]

☐ Vrijgezel/alleenstaand (*)

☐ Ik heb een partner, maar we wonen nog apart

☐ Samenwonend met partner

☐ Gehuwd

☐ Weduwe/weduwnaar (*)

[**Tip voor de interviewer:** indien deelnemer vraag beantwoordt met (*) --> vragen in vragenlijst aangeduid met (*) **NIET** stellen. In dit geval duidt u aan bij de antwoordmogelijkheden dat deze vraag **NIET van toepassing** is voor deze persoon!]

A3) Heeft u (klein)kinderen?

☐ Neen

☐ Ja

A4) In welke omgeving woont u? [overloop onderstaande mogelijkheden]

☐ Op het platteland

☐ In een dorp/gemeente

☐ Aan de rand van de stad

☐ In een stad

A5) Bent u het afgelopen jaar verhuisd naar een ander adres?

☐ Ja ☐ Neen

A6) Bent u in staat om recht te staan (bv. een recente operatie gehad, rugklachten,...)?

☐ Ja ik kan rechtstaan ☐ Neen ik kan niet rechtstaan

Indien neen en/of proefpersoon aangeeft ernstig beperkt te zijn waardoor hij/zij nauwelijks kan rechtstaan (en dit duidelijk blijkt uit zijn/haar fysieke toestand) is hij/zij niet geschikt voor verdere deelname aan het onderzoek. Bijgevolg dient het interview te worden afgerond na deze vraag. [**LEES:** De vragenlijst is omwille van uw gezondheidstoestand niet van toepassing voor u, maar we willen u toch bedanken voor uw interesse!]

Indien ja, het interview kan worden verdergezet [geef het vervolg van de vragenlijst aan de persoon].

6171

ZITTEN EN LICHT INTENSE ACTIVITEITEN

LEES: Onderstaande vragen gaan over zittende activiteiten (zoals tv-kijken) en licht intense activiteiten (zoals kuisen en het uitvoeren van klusjes). Het is belangrijk dat u elke periode van zitten of liggen slechts 1 keer in rekening brengt. Indien u bijvoorbeeld 1 uur in de zetel zat om een boek te lezen terwijl u naar muziek luisterde, reken deze tijd dan als 1 uur lezen indien dit uw voornaamste bezigheid was. Reken deze tijd dan niet opnieuw als 1 uur muziek luisteren. Deze vragen zijn onderverdeeld voor verschillende zittende activiteiten afzonderlijk (tv-kijken, computer- gebruik,...). Voor elke zittende activiteit dient u aan te duiden hoeveel tijd u hierin spendeerde de **afgelopen 7 dagen** en verder dient u ook uw mening te geven over verschillende situaties per zittende activiteit.

B) TELEVISIE (tv) KIJKEN

LEES: We starten met tv-kijken. De volgende vragen gaan over zittend/liggend tv-kijken. Met tv-kijken bedoelen we alle zaken (video's EN films) die op tv worden afgespeeld. DVD's en films bekijken op uw computer worden ook als tv-kijken beschouwd.

Indien de deelnemer aangeeft geen tv te kijken --> ga naar pagina 7: "C) computergebruik"

B1) Hoe lang zat u gemiddeld neer op één dag tijdens de laatste 7 dagen bij het kijken naar tv? Beantwoord deze vragen afzonderlijk voor weekdag en weekenddag.

[**VERDUIDELIJKING INTERVIEWER:** tv- kijken tijdens het eten valt ook hieronder. Laat de deelnemer de vraag afzonderlijk beantwoorden voor een weekdag en een weekenddag.]

[**TIP VOOR DE INTERVIEWER:** Er wordt gepeild naar een gemiddeld aantal uur per dag. Wanneer de deelnemer niet kan antwoorden omwille van een te grote variatie van dag tot dag, vraag dan 'Hoeveel tijd spendeerde u in totaal gedurende de laatste 7 dagen aan tv-kijken?" Deel dit totaal dan zelf door 5 of 2!]

[overloop onderstaande mogelijkheden]

tv-kijken	
**Weekdag**	**Weekenddag**
☐ Niet	☐ Niet
☐ 1 tot 15 minuten/dag	☐ 1 tot 15 minuten/dag
☐ 15 tot 30 minuten/dag	☐ 15 tot 30 minuten/dag
☐ 30 tot 60 minuten/dag	☐ 30 tot 60 minuten/dag
☐ 1 tot 2 uren/dag	☐ 1 tot 2 uren/dag
☐ 2 tot 3 uren/dag	☐ 2 tot 3 uren/dag
☐ 3 tot 4 uren/dag	☐ 3 tot 4 uren/dag
☐ 4 tot 5 uren/dag	☐ 4 tot 5 uren/dag
☐ 5 tot 6 uren/dag	☐ 5 tot 6 uren/dag
☐ 6 tot 7 uren/dag	☐ 6 tot 7 uren/dag
☐ Meer dan 7 uren/dag	☐ Meer dan 7 uren/dag

6171

Geef met behulp van de antwoordkaart (1) aan in welke mate u akkoord gaat met elk van onderstaande stellingen.

[**TIP interviewer:** Overloop elke stelling afzonderlijk]	**Helemaal niet akkoord**	**Eerder niet akkoord**	**Neutraal**	**Eerder akkoord**	**Helemaal akkoord**	**Niet van toepassing**
B2) Ik vind tv-kijken leuk.	☐	☐	☐	☐	☐	
B3) Tv-kijken verhindert me om andere belangrijkere dingen te doen.	☐	☐	☐	☐	☐	
B4) Ik kijk graag verschillende uren aan een stuk tv.	☐	☐	☐	☐	☐	
B5) Tv-kijken is mijn manier om te ontspannen.	☐	☐	☐	☐	☐	
**(*)** B6) Mijn partner vindt dat ik te veel tv kijk.	☐	☐	☐	☐	☐	☐
B7) Ik vind van mezelf dat ik te veel tv kijk.	☐	☐	☐	☐	☐	
B8) Ik zou het zien zitten om de tijd die ik tv kijk te verminderen.	☐	☐	☐	☐	☐	
B9) Ik zou het zien zitten om tijdens het weekend overdag (tot 17 uur) geen tv te kijken.	☐	☐	☐	☐	☐	
B10) Ik zou het zien zitten om de tv niet aan te zetten tijdens het eten.	☐	☐	☐	☐	☐	
**(*)** B11) Mijn partner moedigt me aan om minder tv te kijken.	☐	☐	☐	☐	☐	☐
B12) Mijn vrienden moedigen me aan om minder tv te kijken.	☐	☐	☐	☐	☐	

[**TIP interviewer:** Indien de persoon aangeeft bijvoorbeeld overdag geen tv te kijken, dienen ze "helemaal akkoord" te antwoorden. Dit geldt ook zo voor alle gelijkaardige vragen die beginnen met: "zou u het zien zitten om....". Dus als de persoon aangeeft dit al te doen, dan dient u de antwoordmogelijkheid "helemaal akkoord" aan te duiden".]

6171

B13) Ik denk dat mensen van mijn leeftijd beter niet meer dan... [overloop onderstaande mogelijkheden]

- ☐ 30 minuten tv kijken per dag
- ☐ 1 uur tv kijken per dag
- ☐ 2 uur tv kijken per dag
- ☐ 3 uur tv kijken per dag
- ☐ 4 uur tv kijken per dag
- ☐ 5 uur tv kijken per dag

B14) Waar staat het tv-toestel waar u het meest naar kijkt? [overloop onderstaande mogelijkheden]

- ☐ In de keuken
- ☐ In de woonkamer
- ☐ In een aparte kamer
- ☐ In de slaapkamer
- ☐ In een andere kamer (noteer) ______________________

(*) B15) Hoeveel tijd kijkt uw <u>partner</u> gemiddeld naar tv? [overloop onderstaande mogelijkheden]

- ☐ Niet van toepassing
- ☐ Kijkt geen tv
- ☐ 1 tot 15 minuten/dag
- ☐ 15 tot 30 minuten/dag
- ☐ 30 tot 60 minuten/dag
- ☐ 1 tot 2 uur/dag
- ☐ 2 tot 3 uur/dag
- ☐ 3 tot 4 uur/dag
- ☐ 4 tot 5 uren/dag
- ☐ 5 tot 6 uren/dag
- ☐ 6 tot 7 uren/dag
- ☐ Meer dan 7 uren/dag

(*) B16) Hoe vaak kijkt u SAMEN met uw partner naar tv? [overloop onderstaande mogelijkheden]

- ☐ Niet van toepassing
- ☐ Nooit
- ☐ Zelden
- ☐ Soms
- ☐ Vaak
- ☐ Heel vaak

Er zijn verschillende situaties waarin u uw zittend gedrag tijdens tv-kijken kan verminderen of (meer) kan onderbreken door even recht te staan. Geef met behulp van de antwoordkaart (2) aan in welke mate u het zou zien zitten om volgende zaken uit te voeren.

LEES: In welke mate zou u het zien zitten om.... (voor elke stelling afzonderlijk)

[**TIP interviewer:** Overloop elke stelling afzonderlijk]	**Ik zie dit helemaal niet zitten**	**Ik zie dit eerder niet zitten**	**Neutraal**	**Ik zie dit eerder wel zitten**	**Ik zie dit zeker zitten**
B17) taken rechtstaand uit te voeren terwijl u tv aan het kijken bent (zoals strijken, klusjes uitvoeren, rustig spelen met (klein)kinderen,...).	☐	☐	☐	☐	☐
B18) een uur tv-kijken te vervangen door lichte inspanningen (vb: kuisen, klusjes).	☐	☐	☐	☐	☐
B19) tijdens de reclame op tv kort recht te staan.	☐	☐	☐	☐	☐
B20) recht te staan om van zender te veranderen.	☐	☐	☐	☐	☐

[**TIP interviewer:** Indien de persoon aangeeft bepaalde zaken reeds te doen --> aanduiden "ik zie dit zeker zitten".]

C) COMPUTERGEBRUIK

LEES: De vragen omtrent tv-kijken zijn afgerond en nu gaan we over naar computergebruik. De volgende vragen gaan over uw zittend computergebruik. Met computergebruik bedoelen we: E-mailen, websites bezoeken, chatten, sociale netwerksites (facebook,...) bezoeken, spelletjes spelen,... op een vaste computer of een laptop. Belangrijk is dat u hier ook rekening houdt met het gebruik van **tablets** (handcomputer zoals iPad). Ook internetgebruik op een **smartphone** valt hieronder.

[**TIP interviewer:** indien deelnemer niet weet wat tablets of smartphones zijn, moet je hier niet verder op ingaan. Benadruk nog eens dat het om computergebruik gaat (laptop en vaste computer).]

⚠ **Indien de deelnemer aangeeft bovenstaande zaken (computer, tablet en smartphone) niet te gebruiken --> ga naar pagina 10: "D) gemotoriseerd transport"**

C1) Hoe lang zat/lag u gemiddeld neer op één dag tijdens de laatste 7 dagen bij het gebruik van de computer? Beantwoord deze vraag afzonderlijk voor weekdag en weekenddag.

[**VERDUIDELIJKING INTERVIEWER:** Laat de deelnemer de vraag afzonderlijk beantwoorden voor een weekdag en een weekenddag.]

[**TIP VOOR DE INTERVIEWER:** Er wordt gepeild naar een gemiddeld aantal uur per dag. Wanneer de deelnemer niet kan antwoorden omwille van een te grote variatie van dag tot dag, vraag dan 'Hoeveel tijd spendeerde u in totaal gedurende de laatste 7 dagen aan computer-gebruik?" Deel dit totaal dan zelf door 5 of 2!]

[overloop onderstaande mogelijkheden]

Computer gebruiken	
**Weekdag**	**Weekenddag**
☐ Niet	☐ Niet
☐ 1 tot 15 minuten/dag	☐ 1 tot 15 minuten/dag
☐ 15 tot 30 minuten/dag	☐ 15 tot 30 minuten/dag
☐ 30 tot 60 minuten/dag	☐ 30 tot 60 minuten/dag
☐ 1 tot 2 uren/dag	☐ 1 tot 2 uren/dag
☐ 2 tot 3 uren/dag	☐ 2 tot 3 uren/dag
☐ 3 tot 4 uren/dag	☐ 3 tot 4 uren/dag
☐ 4 tot 5 uren/dag	☐ 4 tot 5 uren/dag
☐ 5 tot 6 uren/dag	☐ 5 tot 6 uren/dag
☐ 6 tot 7 uren/dag	☐ 6 tot 7 uren/dag
☐ Meer dan 7 uren/dag	☐ Meer dan 7 uren/dag

(*) C2) Hoeveel tijd gebruikt uw <u>partner</u> gemiddeld een computer (denk aan tablets, internet op smartphone, laptop, computer,...)? [overloop onderstaande mogelijkheden]

- ☐ Niet van toepassing
- ☐ Kijkt geen tv
- ☐ 1 tot 15 minuten/dag
- ☐ 15 tot 30 minuten/dag
- ☐ 30 tot 60 minuten/dag
- ☐ 1 tot 2 uur/dag
- ☐ 2 tot 3 uur/dag
- ☐ 3 tot 4 uur/dag
- ☐ 4 tot 5 uren/dag
- ☐ 5 tot 6 uren/dag
- ☐ 6 tot 7 uren/dag
- ☐ Meer dan 7 uren/dag

(*) C3) Hoe vaak gebruikt u een computer en dit op <u>hetzelfde moment</u> wanneer uw partner in dezelfde ruimte een andere computer gebruikt? [overloop onderstaande mogelijkheden]

- ☐ Niet van toepassing
- ☐ Nooit
- ☐ Zelden
- ☐ Soms
- ☐ Vaak
- ☐ Heel vaak

Er zijn verschillende situaties waarin u uw zittend gedrag tijdens computergebruik kan verminderen of (meer) kan onderbreken door even recht te staan. Geef met behulp van de antwoordkaart (2) aan in welke mate u het zou zien zitten om volgende zaken uit te voeren.

<u>LEES:</u> In welke mate zou u het zien zitten om .... (voor elke stelling afzonderlijk)

[**<u>TIP interviewer</u>**: indien persoon aangeeft bepaalde zaken reeds te doen --> aanduiden "ik zie dit zeker zitten".]

[**<u>TIP interviewer:</u>** Overloop elke stelling afzonderlijk]	**Ik zie dit helemaal niet zitten**	**Ik zie dit eerder niet zitten**	**Neutraal**	**Ik zie dit eerder wel zitten**	**Ik zie dit zeker zitten**
C4) rechtstaand de computer te gebruiken.	☐	☐	☐	☐	☐
C5) een uur computergebruik te vervangen door lichte inspanningen (vb: kuisen, klusjes).	☐	☐	☐	☐	☐
C6) na 30 minuten de computer te hebben gebruikt gedurende enkele minuten recht te staan.	☐	☐	☐	☐	☐

6171

Geef met behulp van de antwoordkaart (1) aan in welke mate u akkoord gaat met elk van de volgende stellingen over computergebruik.

[**TIP interviewer:** Overloop elke stelling afzonderlijk]	**Helemaal niet akkoord**	**Eerder niet akkoord**	**Neutraal**	**Eerder akkoord**	**Helemaal akkoord**	**Niet van toepassing**
C7) Ik vind het leuk om de computer te gebruiken.	☐	☐	☐	☐	☐	
C8) De computer gebruiken verhindert me belangrijkere zaken te doen.	☐	☐	☐	☐	☐	
C9) Ik vind het plezant gedurende een lange tijd de computer te gebruiken.	☐	☐	☐	☐	☐	
C10) De computer gebruiken is mijn manier om te ontspannen.	☐	☐	☐	☐	☐	
**(*)** C11) Mijn partner vindt dat ik te veel de computer gebruik.	☐	☐	☐	☐	☐	☐
C12) Ik vind van mezelf dat ik te veel de computer gebruik.	☐	☐	☐	☐	☐	
C13) Ik zou het zien zitten om de computer gedurende enkele dagen in de week niet te gebruiken.	☐	☐	☐	☐	☐	
C14) Ik zou het zien zitten om de tijd die ik spendeer aan computergebruik te verminderen.	☐	☐	☐	☐	☐	
**(*)** C15) Mijn partner moedigt me aan om minder de computer te gebruiken.	☐	☐	☐	☐	☐	☐
C16) Mijn vrienden moedigen me aan om minder de computer te gebruiken.	☐	☐	☐	☐	☐	

[**TIP interviewer:** Indien de persoon aangeeft bijvoorbeeld reeds enkele dagen in de week de computer niet te gebruiken, dienen ze "helemaal akkoord" te antwoorden. Dit geldt ook zo voor alle gelijkaardige vragen die beginnen met: "zou u het zien zitten om....". Dus als de persoon aangeeft dit al te doen, dan dient u de antwoordmogelijkheid "helemaal akkoord" aan te duiden".]

D) GEMOTORISEERD TRANSPORT

1) Onder gemotoriseerd transport verstaan we: auto, brommer, moto, trein, tram of bus.
2) Bij elke vraag staat één van onderstaande cirkels (toon bijhorende fotokaart horende bij cirkel):

FOTO GEMOTORISEERD TRANSPORT OF FOTO ACTIEF TRANSPORT

Maak in het begin van dit stuk duidelijk dat fotokaart aantoont over welke vervoersmanieren het gaat!
3) Indien u nooit één van bovenstaande vervoersmiddelen gebruikt (als passagier of als bestuurder) dan mag u verder naar pagina 15: " E) Huishoudelijke bezigheden en telefoneren".

VERPLAATSINGEN

D1) Hoe lang zat u gemiddeld op één dag tijdens de laatste 7 dagen bij gemotoriseerde verplaatsingen. Hou hier dus rekening met uw transport voor verschillende zaken (vb. naar winkel, vrienden opzoeken,...). Denk aan het gebruik van een auto, bus, trein, tram, moto of brommer; reken fietsen niet mee.

[**VERDUIDELIJKING INTERVIEWER:** bevraag dit apart voor weekdag en weekenddag & elektrische fiets valt hier niet onder & deze tijd omvat zowel het transport waarbij men passagier en bestuurder was.]

[**TIP INTERVIEWER:** er wordt gepeild naar een gemiddeld aantal uur per dag. Wanneer de deelnemer niet kan antwoorden omwille van een te grote variatie van dag tot dag, vraag dan "Hoeveel tijd spendeerde u in totaal gedurende de laatste 7 dagen aan het zitten tijdens verplaatsingen met een gemotoriseerd voertuig. Deel dit totaal dan zelf door 5 of 2!]

FOTO GEMOTORISEERD TRANSPORT

[overloop onderstaande mogelijkheden]

Weekdag	Weekenddag
☐ Niet	☐ Niet
☐ 1 tot 15 minuten/dag	☐ 1 tot 15 minuten/dag
☐ 15 tot 30 minuten/dag	☐ 15 tot 30 minuten/dag
☐ 30 tot 45 minuten/dag	☐ 30 tot 45 minuten/dag
☐ 45 tot 60 minuten/dag	☐ 45 tot 60 minuten/dag
☐ 60 tot 90 minuten/dag	☐ 60 tot 90 minuten/dag
☐ 90 tot 120 minuten/dag	☐ 90 tot 120 minuten/dag
☐ 2 tot 2.5 uren/dag	☐ 2 tot 2.5 uren/dag
☐ 2.5 tot 3 uren/dag	☐ 2.5 tot 3 uren/dag
☐ 3 tot 4 uren/dag	☐ 3 tot 4 uren/dag
☐ 4 tot 5 uren/dag	☐ 4 tot 5 uren/dag
☐ 5 tot 6 uren/dag	☐ 5 tot 6 uren/dag
☐ 6 tot 7 uren/dag	☐ 6 tot 7 uren/dag
☐ Meer dan 7 uren per dag	☐ Meer dan 7 uren per dag

FOTO GEMOTORISEERD TRANSPORT

D2) Hoeveel tijd van de zojuist vermelde tijd was u zelf bestuurder van een gemotoriseerd voertuig (brommer, moto of auto)? [overloop mogelijkheden in onderstaande tabel]

[**VERDUIDELIJKING INTERVIEWER:** bevraag dit opnieuw apart voor weekdag en weekenddag & elektrische fiets valt hier opnieuw niet onder & maak duidelijk dat ze zich baseren op de vorige vraag.]

[**TIP INTERVIEWER:** er wordt gepeild naar een gemiddeld aantal uur per dag. Wanneer de deelnemer niet kan antwoorden omwille van een te grote variatie van dag tot dag, vraag dan "Hoeveel tijd spendeerde u in totaal gedurende de laatste 7 dagen aan het zitten tijdens verplaatsingen wanneer u zelf bestuurder was van een gemotoriseerd voertuig. Deel dit totaal dan zelf door 5 of 2!]

Weekdag	Weekenddag
☐ Niet	☐ Niet
☐ 1 tot 15 minuten/dag	☐ 1 tot 15 minuten/dag
☐ 15 tot 30 minuten/dag	☐ 15 tot 30 minuten/dag
☐ 30 tot 45 minuten/dag	☐ 30 tot 45 minuten/dag
☐ 45 tot 60 minuten/dag	☐ 45 tot 60 minuten/dag
☐ 60 tot 90 minuten/dag	☐ 60 tot 90 minuten/dag
☐ 90 tot 120 minuten/dag	☐ 90 tot 120 minuten/dag
☐ 2 tot 2.5 uren/dag	☐ 2 tot 2.5 uren/dag
☐ 2.5 tot 3 uren/dag	☐ 2.5 tot 3 uren/dag
☐ 3 tot 4 uren/dag	☐ 3 tot 4 uren/dag
☐ 4 tot 5 uren/dag	☐ 4 tot 5 uren/dag
☐ 5 tot 6 uren/dag	☐ 5 tot 6 uren/dag
☐ 6 tot 7 uren/dag	☐ 6 tot 7 uren/dag
☐ Meer dan 7 uren per dag	☐ Meer dan 7 uren per dag

D3) Gelieve aan te geven hoeveel tijd u gemiddeld per dag spendeerde aan wandelen buitenshuis op een rustig tempo (bv: slenteren = rustig stappen, wandelen op "gemak", niet gehaast) als deel van uw transport (bv. iemand bezoeken, naar bus wandelen, naar winkel wandelen,...) tijdens de laatste 7 dagen. [overloop onderstaande mogelijkheden]

FOTO ACTIEF TRANSPORT

[**VERDUIDELIJKING INTERVIEWER:** het gaat hier enkel over wandelen op een rustig tempo buitenshuis & wandelen moet doel hebben zoals bv. naar winkel gaan en wandelen als ontspanning/in vrije tijd hoort hier niet bij & apart bevragen voor weekdag en weekenddag]

Weekdag	Weekenddag
☐ Niet	☐ Niet
☐ 1 tot 15 minuten/dag	☐ 1 tot 15 minuten/dag
☐ 15 tot 30 minuten/dag	☐ 15 tot 30 minuten/dag
☐ 30 tot 45 minuten/dag	☐ 30 tot 45 minuten/dag
☐ 45 tot 60 minuten/dag	☐ 45 tot 60 minuten/dag
☐ 1 tot 2 uur/dag	☐ 1 tot 2 uur/dag
☐ Meer dan 2 uur/dag	☐ Meer dan 2 uur/dag

6171

LEES: Voor onderstaande vragen wordt er onder transport steeds het gebruik van gemotoriseerde vervoersmiddelen verstaan (auto, brommer, moto, trein, tram of bus) waarvan u als bestuurder of als passagier gebruik van maakt. Elektrische fiets valt hier ook NIET onder. FOTO GEMOTORISEERD TRANSPORT

Geef met behulp van antwoordkaart (1) aan in welke mate u akkoord gaat met elk van onderstaande stellingen.

[**TIP interviewer:** Overloop elke stelling afzonderlijk]	**Helemaal niet akkoord**	**Eerder niet akkoord**	**Neutraal**	**Eerder akkoord**	**Helemaal akkoord**	**Niet van toepassing**
D4) Ik vind het leuk om me met een gemotoriseerd voertuig te verplaatsen.	☐	☐	☐	☐	☐	
D5) Ik vind het leuk om als passagier tijdens gemotoriseerd transport aan bepaalde zaken te werken of uit te rusten (bv. naaiwerk, telefoneren,...).	☐	☐	☐	☐	☐	☐
D6) Ik voel me lui als ik op mijn bestemming aankom na mijn gemotoriseerd transport	☐	☐	☐	☐	☐	
D7) Ik vind van mezelf dat ik te veel gebruik maak van gemotoriseerd transport.	☐	☐	☐	☐	☐	
D8) Ik zou het zien zitten om spontaan een halte vroeger uit te stappen uit tram/bus en het resterende eind te wandelen.	☐	☐	☐	☐	☐	
D9) Ik zou het zien zitten om me spontaan wat verder te parkeren met de auto en het resterende eind te wandelen.	☐	☐	☐	☐	☐	
D10) Ik zou het zien zitten om spontaan de fiets te nemen of te wandelen ook al heb ik een auto ter beschikking.	☐	☐	☐	☐	☐	
**(*)** D11) Mijn partner moedigt me aan om me op een (meer) actieve manier (fietsen, wandelen) te verplaatsen.	☐	☐	☐	☐	☐	☐
D12) Mijn vrienden moedigen me aan om me op een (meer) actieve manier (fietsen, wandelen) te verplaatsen.	☐	☐	☐	☐	☐	
**(*)** D13) Mijn partner vindt dat ik te veel gebruik maak van gemotoriseerd transport.	☐	☐	☐	☐	☐	☐

[**TIP interviewer:** Indien de persoon aangeeft bijvoorbeeld reeds spontaan een halte vroeger uit te stappen, dienen ze "helemaal akkoord" te antwoorden. Dit geldt ook zo voor alle gelijkaardige vragen die beginnen met: "zou u het zien zitten om....". Dus als de persoon aangeeft dit al te doen, dan dient u de antwoordmogelijkheid "helemaal akkoord" aan te duiden".]

FOTO ACTIEF TRANSPORT

D14) Wat zijn voor u de voornaamste redenen om u niet actief (wandelen of fietsen) te verplaatsen?

[**VERDUIDELIJKING INTERVIEWER:** lees één voor één de antwoordmogelijkheden & meerdere antwoorden zijn mogelijk]

- ☐ De omgeving is niet veilig om te wandelen of te fietsen
- ☐ Actief transport (wandelen of fietsen) is niet aantrekkelijk (geen groene omgeving in de buurt, saai,...)
- ☐ Actief transport is vermoeiend (zweten, bagage dragen,...)
- ☐ De voorzieningen voor openbaar vervoer zijn goed uitgerust (bushaltes dicht bij de deur,...)
- ☐ Tijdsdruk
- ☐ Te grote afstand
- ☐ Weersomstandigheden
- ☐ Het combineren van verschillende taken (naar bakker, iemand bezoeken,....)
- ☐ Financiële redenen (terugbetaling openbaar vervoer, prijsverlagingen voor 65-plussers)
- ☐ Ik kan/durf niet te fietsen
- ☐ Ik kan niet lang wandelen
- ☐ Als ik wandel krijg ik pijn in knie, rug,...
- ☐ Als ik fiets krijg ik pijn in knie, rug,...
- ☐ Andere (noteer) ______________________
- ☐ Ik verplaats me altijd actief (wandelen en/of fietsen)

(*) D15) Hoe verplaatst uw partner zich meestal...

Gemotoriseerd transport	Actief transport (wandelen/fietsen)	Niet van toepassing (geen partner)
☐	☐	☐

Er zijn verschillende situaties waarin u uw zittend gedrag tijdens gemotoriseerd transport kan verminderen of (meer) kan onderbreken door even recht te staan. Geef met behulp van de antwoordkaart (2) aan in welke mate u het zou zien zitten om volgende zaken uit te voeren.

FOTO GEMOTORISEERD TRANSPORT

LEES: in welke mate zou u het zien zitten om... (voor elke stelling afzonderlijk)

[**TIP interviewer**: indien persoon aangeeft bepaalde zaken reeds te doen --> aanduiden "ik zie dit zeker zitten".]

[**TIP interviewer:** Overloop elke stelling afzonderlijk]	Ik zie dit helemaal niet zitten	Ik zie dit eerder niet zitten	Neutraal	Ik zie dit eerder wel zitten	Ik zie dit zeker zitten
D16) spontaan recht te staan op de bus, trein of tram in plaats van neer te zitten.	☐	☐	☐	☐	☐
D17) na 30 minuten zitten in bus, trein of tram even kort te gaan rechtstaan.	☐	☐	☐	☐	☐

FOTO GEMOTORISEERD TRANSPORT

Geef over onderstaande beweringen uw mening i.v.m. de omgeving waarin u zich begeeft tijdens uw gemotoriseerd transport. Beoordeel hier de omgeving op ongeveer 10 à 15 minuten wandelen of op 1 km van uw woning. Doe dit opnieuw met behulp van een antwoordkaart (1).

[**TIP interviewer:** Overloop elke stelling afzonderlijk]	**Helemaal niet akkoord**	**Eerder niet akkoord**	**Neutraal**	**Eerder akkoord**	**Helemaal akkoord**
D18) Als ik me buitenshuis begeef naar openbare plaatsen (winkels/station/...) kan ik gemakkelijk parkeren.	☐	☐	☐	☐	☐
D19) Via informatieborden langs de weg en/of radio krijg ik veel info over cinema/theater of sportwedstrijden waar ik veel zit.	☐	☐	☐	☐	☐

(*) D20) Hoe vaak verplaatst u zich SAMEN met uw partner met gemotoriseerd transport?

[**VERDUIDELIJKING INTERVIEWER:** bevraag dit apart voor weekdag en weekenddag]

[overloop onderstaande mogelijkheden]

FOTO GEMOTORISEERD TRANSPORT

**Weekdag**	**Weekenddag**
☐ Nooit<br>☐ Zelden<br>☐ Soms<br>☐ Vaak<br>☐ Heel vaak	☐ Nooit<br>☐ Zelden<br>☐ Soms<br>☐ Vaak<br>☐ Heel vaak

☐ Niet van toepassing (geen partner)

LEES: U bent al voorbij de helft van de vragenlijst!

6171

E) HUISHOUDELIJKE BEZIGHEDEN EN TELEFONEREN

LEES: nu gaan we u enkele vragen stellen over huishoudelijke taken en telefoneren in huis.

E1) Hoeveel tijd bracht u gemiddeld per dag al zittend/liggend door tijdens huishoudelijke taken en ook al zittend/liggend tijdens thuis telefoneren tijdens de laatste 7 dagen? Mogelijke huishoudelijke taken zijn: koken, strijken, kleine herstellingen uitvoeren,...

[**VERDUIDELIJKING INTERVIEWER:** onder kleine herstellingen verstaan we bijvoorbeeld een lamp of batterij vervangen & bevraag huishoudelijke taken afzonderlijk van telefoneren]

[**TIP INTERVIEWER:** er wordt gepeild naar een gemiddeld aantal uur per dag. Wanneer de deelnemer niet kan antwoorden omwille van een te grote variatie van dag tot dag, vraag dan "Hoeveel tijd spendeerde u in totaal gedurende de laatste 7 dagen aan huishoudelijke taken OF telefoneren in huis? Deel dit totaal dan zelf door 5 of 2!]

[overloop onderstaande mogelijkheden]

**Huishoudelijke taken (zittend)**	
Weekdag	Weekenddag
☐ Niet	☐ Niet
☐ 1 tot 15 minuten/dag	☐ 1 tot 15 minuten/dag
☐ 15 tot 30 minuten/dag	☐ 15 tot 30 minuten/dag
☐ 30 tot 60 minuten/dag	☐ 30 tot 60 minuten/dag
☐ 1 tot 2 uren/dag	☐ 1 tot 2 uren/dag
☐ 2 tot 3 uren/dag	☐ 2 tot 3 uren/dag
☐ 3 tot 4 uren/dag	☐ 3 tot 4 uren/dag
☐ 4 tot 5 uren/dag	☐ 4 tot 5 uren/dag
☐ 5 tot 6 uren/dag	☐ 5 tot 6 uren/dag
☐ 6 tot 7 uren/dag	☐ 6 tot 7 uren/dag
☐ Meer dan 7 uren/dag	☐ Meer dan 7 uren/dag

**Telefoneren (zittend)**	
Weekdag	Weekenddag
☐ Niet	☐ Niet
☐ 1 tot 15 minuten/dag	☐ 1 tot 15 minuten/dag
☐ 15 tot 30 minuten/dag	☐ 15 tot 30 minuten/dag
☐ 30 tot 60 minuten/dag	☐ 30 tot 60 minuten/dag
☐ 1 tot 2 uren/dag	☐ 1 tot 2 uren/dag
☐ 2 tot 3 uren/dag	☐ 2 tot 3 uren/dag
☐ 3 tot 4 uren/dag	☐ 3 tot 4 uren/dag
☐ 4 tot 5 uren/dag	☐ 4 tot 5 uren/dag
☐ 5 tot 6 uren/dag	☐ 5 tot 6 uren/dag
☐ 6 tot 7 uren/dag	☐ 6 tot 7 uren/dag
☐ Meer dan 7 uren/dag	☐ Meer dan 7 uren/dag

6171

De volgende stellingen zijn verschillende meningen over zittende activiteiten in het huishouden en thuis telefoneren. Geef met behulp van antwoordkaart (1) aan in welke mate u akkoord gaat met elk van de onderstaande stellingen.

[**TIP interviewer:** Overloop elke stelling afzonderlijk]	**Helemaal niet akkoord**	**Eerder niet akkoord**	**Neutraal**	**Eerder akkoord**	**Helemaal akkoord**	**Niet van toepassing**
E2) Ik vind het leuk om te zitten tijdens huishoudelijke taken.	☐	☐	☐	☐	☐	
E3) Wanneer ik zittend werk in het huishouden kan ik sneller werken.	☐	☐	☐	☐	☐	
E4) Ik zou liever meer rechtstaan tijdens huishoudelijke taken.	☐	☐	☐	☐	☐	
E5) Ik vind van mezelf dat ik meer kan rechtstaan tijdens huishoudelijke taken.	☐	☐	☐	☐	☐	
E6) Ik zou het zien zitten om rechtstaand te telefoneren.	☐	☐	☐	☐	☐	
E7) Ik zou het zien zitten om taken in de keuken rechtstaand uit te voeren.	☐	☐	☐	☐	☐	
**(*)** E8) Mijn partner moedigt me aan om minder te zitten tijdens huishoudelijke taken.	☐	☐	☐	☐	☐	☐
**(*)** E9) Huishoudelijke taken (en telefoneren) worden door mijn partner meestal zittend uitgevoerd.	☐	☐	☐	☐	☐	☐

[**TIP interviewer:** Indien de persoon aangeeft bijvoorbeeld reeds rechtstaand te telefoneren, dienen ze "helemaal akkoord" te antwoorden. Dit geldt ook zo voor alle gelijkaardige vragen die beginnen met: "zou u het zien zitten om....". Dus als de persoon aangeeft dit al te doen, dan dient u de antwoordmogelijkheid "helemaal akkoord" aan te duiden".]

Geef aan of u het liefst volgende huishoudelijke taken al zittend of al rechtstaand zou uitvoeren.

	**Liefst rechtstaand**	**Liefst zittend**
E10) Telefoneren	☐	☐
E11) Aardappelen schillen/groenten snijden	☐	☐
E12) Strijken	☐	☐
E13) Was opvouwen	☐	☐
E14) Tanden poetsen	☐	☐
E15) Kleine herstellingen uitvoeren	☐	☐

[**VERDUIDELIJKING INTERVIEWER:** hoe zien ze dit zitten in de toekomst & als ze bijvoorbeeld de was nooit opvouwen, bevraag dit dan hypothetisch.]

6171

Er zijn verschillende situaties waarin u uw zittend gedrag in het huishouden kan verminderen of (meer) kan onderbreken door even recht te staan. Geef met behulp van de antwoordkaart (2) aan in welke mate u het zou zien zitten om volgende zaken uit te voeren.

[**TIP interviewer**: indien persoon aangeeft bepaalde zaken reeds te doen -> aanduiden "ik zie dit zeker zitten".]

LEES: in welke mate zou u het zien zitten om... (voor elke stelling afzonderlijk)

[**TIP interviewer:** Overloop elke stelling afzonderlijk]	**Ik zie dit helemaal niet zitten**	**Ik zie dit eerder niet zitten**	**Neutraal**	**Ik zie dit eerder wel zitten**	**Ik zie dit zeker zitten**
E16) meer huishoudelijke taken rechtstaand uit te voeren.	☐	☐	☐	☐	☐
E17) toestellen te verplaatsen zodat u moet rechtstaan om deze te kunnen bedienen (printer, afstandsbediening tv,...).	☐	☐	☐	☐	☐

(*) Geef met behulp van de antwoordkaart (1) aan in welke mate u akkoord gaat met volgende stellingen.
Ik ben er zeker van dit ik mijn zittende activiteiten thuis meer kan onderbreken, ook al (herhaal voor elke stelling)

	**Helemaal niet akkoord**	**Eerder niet akkoord**	**Neutraal**	**Eerder akkoord**	**Helemaal akkoord**	**Niet van toepassing**
E18) doet mijn partner dit niet.	☐	☐	☐	☐	☐	☐
E19) steunt/begrijpt mijn partner mij niet.	☐	☐	☐	☐	☐	☐

Hoe bekijkt u de nabije omgeving van uw woonplaats (binnen een straal van 1 km of op 10-15 minuten wandelen)? Geef met behulp van de antwoordkaart (1) aan in welke mate u akkoord gaat met onderstaande stellingen.

[**TIP interviewer:** Overloop elke stelling afzonderlijk]	**Helemaal niet akkoord**	**Eerder niet akkoord**	**Neutraal**	**Eerder akkoord**	**Helemaal akkoord**
E20) De meeste woningen in mijn buurt zijn alleenstaande woningen (open bebouwing).	☐	☐	☐	☐	☐
E21) Er zijn veel winkels op wandelafstand van mijn huis.	☐	☐	☐	☐	☐
E22) Er is een halte van het openbaar vervoer (bus en/of tram) op wandelafstand van mijn huis.	☐	☐	☐	☐	☐
E23) Er is een park op wandelafstand van mijn huis.	☐	☐	☐	☐	☐
E24) Wandelen is gevaarlijk omwille van het verkeer in mijn buurt.	☐	☐	☐	☐	☐
E25) Wandelen in mijn buurt is gevaarlijk omwille van de hoge kans op misdaad.	☐	☐	☐	☐	☐
E26) In mijn buurt staan er bomen langs de straten.	☐	☐	☐	☐	☐
E27) Ik vind mijn buurt een goede plaats om in te leven.	☐	☐	☐	☐	☐
E28) De omgeving nodigt uit om te bewegen (geen heuvels, goed onderhouden voetpad,...).	☐	☐	☐	☐	☐

Hoeveel van onderstaande niet draagbare toestellen zijn aanwezig bij u thuis EN maakt u ZELF ook gebruik van?

[**VERDUIDELIJKING INTERVIEWER (voor alle onderstaande vragen):** indien deelnemer geen antwoord kan geven, overloop stap voor stap de woning & het moeten toestellen zijn waar ze zelf ook gebruik van maken!]

	Geen	1	2	3	4	5	Meer dan 5
E29) Televisies	☐	☐	☐	☐	☐	☐	☐
E30) Videospelers/Dvd-spelers	☐	☐	☐	☐	☐	☐	☐
E31) Muziekinstallaties (radio, CD-spelers, stereo,...)	☐	☐	☐	☐	☐	☐	☐
E32) Computer (vaste)	☐	☐	☐	☐	☐	☐	☐
E33) Telefoontoestellen (vaste lijn)	☐	☐	☐	☐	☐	☐	☐

Hoeveel van bovenstaande niet draagbare toestellen bevinden zich in uw slaapkamer?

	Geen	1	2	3	4	5	Meer dan 5
E34) Televisies	☐	☐	☐	☐	☐	☐	☐
E35) Videospelers/Dvd-spelers	☐	☐	☐	☐	☐	☐	☐
E36) Muziekinstallaties (radio, CD-spelers, stereo,...)	☐	☐	☐	☐	☐	☐	☐
E37) Computer (vaste)	☐	☐	☐	☐	☐	☐	☐
E38) Telefoontoestellen (vaste lijn)	☐	☐	☐	☐	☐	☐	☐

Gelieve het aantal draagbare toestellen te noteren die aanwezig zijn bij u thuis EN waarvan u ZELF ook gebruik maakt.

[**VERDUIDELIJKING INTERVIEWER:** indien deelnemer geen smartphone/tablet/... kent, hier niet verder op ingaan]

	Geen	1	2	3	4	5	Meer dan 5
E39) Laptop	☐	☐	☐	☐	☐	☐	☐
E40) GSM (geen smartphone)	☐	☐	☐	☐	☐	☐	☐
E41) Smartphone	☐	☐	☐	☐	☐	☐	☐
E42) Muziekspelers (zoals iPod, MP3,...)	☐	☐	☐	☐	☐	☐	☐
E43)Tablet (iPad, Samsung Galaxy Tab,...)	☐	☐	☐	☐	☐	☐	☐

Hoe beoordeelt u volgende beweringen omtrent de voorzieningen bij u thuis?

[**VERDUIDELIJKING INTERVIEWER:** gebruik hiervoor antwoordkaart (6) & "niet van toepassing"= geen tv of geen zetels]

	Helemaal niet akkoord	Eerder niet akkoord	Neutraal	Eerder akkoord	Helemaal akkoord	Niet van toepassing
E44) De afstandsbediening van de tv ligt steeds binnen handbereik wanneer ik deze nodig heb.	☐	☐	☐	☐	☐	☐
E45) De zetels bij ons thuis zijn comfortabel om gedurende een lange tijd in te zitten.	☐	☐	☐	☐	☐	☐

6171

F) ANDERE ZITTENDE/LIGGENDE ACTIVITEITEN

LEES: tot nu toe hebben we de voorgaande contexten uitgebreid besproken. Nu gaan we andere contexten bevragen, maar hier willen we enkel weten hoeveel tijd u aan deze contexten spendeerde tijdens de laatste 7 dagen.
Hoe lang zat u gemiddeld op één dag tijdens de laatste 7 dagen voor de volgende zittende activiteiten? Onder **zittende activiteiten** worden ook **liggende activiteiten** verstaan. Elke periode van zitten of liggen mag slechts **1 keer in rekening** worden gebracht. Indien u bv. 1 uur in de zetel zat om een boek te lezen terwijl u naar muziek luisterde, reken deze tijd dan als 1 uur lezen indien dit uw voornaamste bezigheid was. Reken deze tijd dan niet opnieuw als 1 uur naar muziek luisteren!

[**VERDUIDELIJKING INTERVIEWER:**
- Geef antwoordkaart (4) aan deelnemer & bevraag dit apart voor weekdagen en weekenddagen
- Vermeld duidelijk de gegeven voorbeelden
- **HOBBY'S**: **vermeld** ook sudoku's, kruiswoordpuzzels,...
- Er wordt gepeild naar een gemiddeld aantal uur per dag. Wanneer de deelnemer niet kan antwoorden omwille van een te grote variatie van dag tot dag, vraag dan "Hoeveel tijd spendeerde u in totaal gedurende de laatste 7 dagen aan... Deel dit totaal dan zelf door 5 of 2!
- **MAALTIJDEN**: eten voor tv moet men hier **niet** meetellen]

		Niet	1 tot 15 minuten per dag	15 tot 30 minuten per dag	30 tot 60 minuten per dag	1 tot 2 uur per dag	2 tot 3 uur per dag	3 tot 4 uur per dag	4 tot 5 uur per dag	5 tot 6 uur per dag	6 tot 7 uur per dag	Meer dan 7 uur per dag
**F1) Zittend lezen** (boek, krant,...)	weekdag	☐	☐	☐	☐	☐	☐	☐	☐	☐	☐	☐
	weekenddag	☐	☐	☐	☐	☐	☐	☐	☐	☐	☐	☐
**F2) Zittend zorgen** voor kleinkinderen, anderen	weekdag	☐	☐	☐	☐	☐	☐	☐	☐	☐	☐	☐
	weekenddag	☐	☐	☐	☐	☐	☐	☐	☐	☐	☐	☐
**F3) Zitten bij hobby's** (vrijwilligers-werk, kaarten, naaien,...)	weekdag	☐	☐	☐	☐	☐	☐	☐	☐	☐	☐	☐
	weekenddag	☐	☐	☐	☐	☐	☐	☐	☐	☐	☐	☐
**F4) Zitten bij sociaal contact** (vrienden bezoeken, café,....)	weekdag	☐	☐	☐	☐	☐	☐	☐	☐	☐	☐	☐
	weekenddag	☐	☐	☐	☐	☐	☐	☐	☐	☐	☐	☐
**F5) Zittend naar muziek luisteren** (radio, CD,...)	weekdag	☐	☐	☐	☐	☐	☐	☐	☐	☐	☐	☐
	weekenddag	☐	☐	☐	☐	☐	☐	☐	☐	☐	☐	☐
**F6) Maaltijden** (ontbijt,...)	weekdag	☐	☐	☐	☐	☐	☐	☐	☐	☐	☐	☐
	weekenddag	☐	☐	☐	☐	☐	☐	☐	☐	☐	☐	☐
**F7)** Middagdutje	weekdag	☐	☐	☐	☐	☐	☐	☐	☐	☐	☐	☐
	weekenddag	☐	☐	☐	☐	☐	☐	☐	☐	☐	☐	☐

Hoe vaak doet u onderstaande situaties op HETZELFDE (tegelijkertijd) moment? (geef antwoordkaart 5)

	Nooit	Zelden	Soms	Vaak	Heel vaak	Niet van toepassing
F8) tv kijken - GSM gebruiken	☐	☐	☐	☐	☐	☐
F9) tv kijken - computer/tablet (handcomputer)	☐	☐	☐	☐	☐	☐
F10) computer - GSM gebruiken	☐	☐	☐	☐	☐	☐
F11) computer - muziek luisteren	☐	☐	☐	☐	☐	☐
F12) GSM - praten met vrienden/familie	☐	☐	☐	☐	☐	☐
F13) GSM - muziek luisteren	☐	☐	☐	☐	☐	☐
F14) Naaien (of ander handwerk) - tv kijken	☐	☐	☐	☐	☐	☐

[**Tip interviewer:** "niet van toepassing" kan enkel worden aangeduid als persoon een bepaald toestel niet gebruikt/heeft of één van beide activiteiten niet doet.]

[**Verduidelijking interviewer:** F12: met praten met vrienden/familie bedoelen we het rechtstreekse sociale contact en dus niet opbellen.]

6171

R) Rechtstaande activiteiten

Gelieve aan te geven hoeveel tijd u gemiddeld per dag spendeerde aan de onderstaande rechtstaande activiteiten/situaties tijdens de laatste 7 dagen (geef antwoordkaart 3).

**VERMELD onderstaande voorbeelden van licht intense activiteiten in het huishouden (RECHTstaand uitgevoerd)**		
Afstoffen **(A)**	Aankleden/uitkleden	Planten water geven
Koken	Spelen met (klein)kinderen (rustig)	Rechtstaand telefoneren
Strijken **(A)**	Eten opdienen	Persoonlijke hygiëne (scheren, make-up,...)
Afwassen, tafel opruimen	Dieren eten geven	De was doen, kleren ophangen voor te drogen,... **(A)**

		Niet	1 tot 15 minuten/dag	15 tot 30 minuten/dag	30 tot 45 minuten/dag	45 tot 60 minuten/dag	1 tot 2 uur/dag	Meer dan 2 uur/dag
**HUISHOUDEN (RECHTSTAAND UITGEVOERD)**								
**R1) Kuisen** (afstoffen,strijken,....) **Opmerking**: enkel voorbeelden in tabel vallen hieronder **(A)**	weekdag	☐	☐	☐	☐	☐	☐	☐
	weekenddag	☐	☐	☐	☐	☐	☐	☐
**R2) Keuken** (koken, voorbereiding,...)	weekdag	☐	☐	☐	☐	☐	☐	☐
	weekenddag	☐	☐	☐	☐	☐	☐	☐
**R3) (Klein)-kinderen** (rustig samen spelen,...)	weekdag	☐	☐	☐	☐	☐	☐	☐
	weekenddag	☐	☐	☐	☐	☐	☐	☐
**TUIN (RECHTSTAAND UITGEVOERD)**								
R4) Planten water geven, dieren eten geven,...	weekdag	☐	☐	☐	☐	☐	☐	☐
	weekenddag	☐	☐	☐	☐	☐	☐	☐
**VERZORGING (RECHTSTAAND UITGEVOERD)**								
**R5) Verzorging** (wassen, scheren, make-up, tanden poetsen,...)	weekdag	☐	☐	☐	☐	☐	☐	☐
	weekenddag	☐	☐	☐	☐	☐	☐	☐
**VRIJE TIJD/HOBBY'S (RECHTSTAAND UITGEVOERD)**								
R6) Winkelen (niet stappen), staand tv-kijken, staand muziek luisteren, lichte stretching, rustige yoga, biljart, staan in kerk,...	weekdag	☐	☐	☐	☐	☐	☐	☐
	weekenddag	☐	☐	☐	☐	☐	☐	☐
**WANDELEN OP RUSTIG TEMPO (bv. slenteren= rustig stappen, niet gehaast)**								
R7) Wandelen op rustig tempo (bv. slenteren) in huis	weekdag	☐	☐	☐	☐	☐	☐	☐
	weekenddag	☐	☐	☐	☐	☐	☐	☐

G) Gezondheid

LEES: deze vragen gaan over uw standpunten ten aanzien van uw gezondheid. Al uw antwoorden worden ook hier strikt vertrouwelijk verwerkt!

Heeft u gedurende de voorbije maand vaak last gehad van een neerslachtig, depressief of hopeloos gevoel?

☐ Ja ☐ Neen

Heeft u gedurende de laatste maand minder plezier of interesse in wat u doet?

☐ Ja ☐ Neen

H) Fysieke activiteit

LEES: denk nu aan alle bewegingsactiviteiten die u de laatste 7 dagen heeft gedaan en dit rekening houdend met volgende activiteiten: huishouden, werken in tuin, zich verplaatsen van en naar verschillende plaatsen, activiteiten in vrije tijd als ontspanning en sport, vrijwilligerswerk,....
Ook indien u denkt geen actief persoon te zijn, vragen we u goed na te denken over onderstaande vragen.

1) Op hoeveel dagen heeft u tenminste 10 minuten aan één stuk gewandeld als u denkt aan de laatste 7 dagen? Denk hierbij aan wandelen thuis, wandelen om van de ene naar de andere plaats te komen, en al het andere wandelen dat u deed tijdens recreatie, sport of vrijetijdsbesteding.

[**VERDUIDELIJKING INTERVIEWER:** deelnemer mag enkel rekening houden met wandelen dat ten minste 10 min heeft geduurd.]

☐ Geen wandelen **Indien geen wandelen --> ga naar 2)**

___ dag(en) per week

Op de dagen dat u ten minste 10 minuten aan één stuk wandelde, hoeveel tijd heeft u daar dan gewoonlijk aan besteed?

___ uur/uren ___ minuten per dag

[**VERDUIDELIJKING INTERVIEWER:** deelnemer mag enkel rekening houden met wandelen dat ten minste 10 min heeft geduurd. Rapporteer de tijd van de hele verplaatsing, dus heen en terug.]

[**TIP VOOR DE INTERVIEWER:** Er wordt gepeild naar een gemiddeld aantal uur per dag. Wanneer de deelnemer niet kan antwoorden omwille van een te grote variatie van dag tot dag, vraag dan 'Hoeveel tijd spendeerde u in totaal gedurende de laatste 7 dagen aan wandelen om ergens heen te gaan?" Deel dit totaal dan zelf door 7!]

2) Op hoeveel dagen heeft u matige fysieke activiteiten verricht, zoals tuinieren, kuisen (vb: ruiten wassen), fietsen op regelmatig tempo, zwemmen op regelmatig tempo of andere fitness activiteiten aan een matige intensiteit als u denkt aan de laatste 7 dagen? Laat wandelen hier buiten beschouwing. Matig intensieve lichamelijke fysieke activiteit laat u iets sneller ademen dan normaal. Denkt u weer alleen aan activiteiten die u ten minste 10 minuten aan één stuk heeft verricht.

[**VERDUIDELIJKING INTERVIEWER:** deelnemer mag enkel rekening houden met matige fysieke inspanningen die ten minste 10 min hebben geduurd.]

☐ Ik heb geen matig intensieve lichamelijke fysieke activiteiten verricht **Ga naar 3)**

___ dag(en) per week

Op de dagen dat u matig intensief lichamelijk fysiek actief was, hoeveel tijd heeft u daar dan gewoonlijk aan besteed?

___ uur/uren ___ minuten per dag

[**VERDUIDELIJKING INTERVIEWER:** deelnemer mag enkel rekening houden met matig fysieke inspanningen die ten minste 10 min hebben geduurd.]

[**TIP VOOR DE INTERVIEWER:** Er wordt gepeild naar een gemiddeld aantal uur per dag. Wanneer de deelnemer niet kan antwoorden omwille van een te grote variatie van dag tot dag, vraag dan 'Hoeveel tijd spendeerde u in totaal gedurende de laatste 7 dagen aan uitvoeren van matig fysieke inspanningen?" Deel dit totaal dan zelf door 7!]

6171

3) Op hoeveel dagen heeft u zware lichamelijke fysieke activiteiten verricht zoals zware lasten tillen, zwaardere taken in tuin/bouw, hout hakken, aerobics, joggen/lopen of snel fietsen als u denkt aan de laatste 7 dagen? Denk alleen aan de activiteiten die u ten minste 10 minuten aan één stuk heeft verricht. Zware lichamelijke fysieke activiteiten zijn activiteiten die veel lichamelijke inspanning kosten en voor een veel snellere ademhaling zorgen.

[**VERDUIDELIJKING INTERVIEWER:** deelnemer mag enkel rekening houden met zware lichamelijke fysieke activiteiten die ten minste 10 min hebben geduurd.]

☐ Ik heb geen zware lichamelijke fysieke activiteiten verricht **Ga naar I)**

___ dag(en) per week

Op de dagen dat u zwaar lichamelijk fysiek actief was, hoeveel tijd heeft u daar dan gewoonlijk aan besteed?

___ uur/uren ___ minuten per dag

[**VERDUIDELIJKING INTERVIEWER:** deelnemer mag enkel rekening houden met zware lichamelijke fysieke activiteiten die ten minste 10 min hebben geduurd.]

[**TIP VOOR DE INTERVIEWER:** Er wordt gepeild naar een gemiddeld aantal uur per dag. Wanneer de deelnemer niet kan antwoorden omwille van een te grote variatie van dag tot dag, vraag dan 'Hoeveel tijd spendeerde u in totaal gedurende de laatste 7 dagen aan zware lichamelijke fysieke activiteiten?" Deel dit totaal dan zelf door 7!]

I) Informatief gedeelte

I1) Wat is uw moedertaal? (gelieve 1 antwoord te geven)

☐ Nederlands
☐ Frans
☐ Turks
☐ Arabisch
☐ Engels
☐ Andere (noteer) ____________________

I2) Wat is het hoogst behaalde diploma of getuigschrift dat u (* en eventueel uw partner) behaald heeft?
[overloop onderstaande mogelijkheden]

UZELF	(*) PARTNER
☐ Lager onderwijs<br>☐ Beroeps secundair onderwijs (BSO)<br>☐ Technisch secundair onderwijs (TSO)<br>☐ Algemeen secundair onderwijs (ASO)<br>☐ Hoger onderwijs, niet-universitair<br>☐ Universitair onderwijs	☐ Weet ik niet<br>☐ Lager onderwijs<br>☐ Beroeps secundair onderwijs (BSO)<br>☐ Technisch secundair onderwijs (TSO)<br>☐ Algemeen secundair onderwijs (ASO)<br>☐ Hoger onderwijs, niet-universitair<br>☐ Universitair onderwijs<br>☐ Niet van toepassing

6171

I3) Hoe kan u uw huidige werksituatie het best omschrijven? [overloop onderstaande mogelijkheden]

- ☐ Ik werk voltijds
- ☐ Ik werk halftijds of parttime
- ☐ Ik ben werkloos/werkzoekende
- ☐ Ik heb een loopbaanonderbreking
- ☐ Ik ben gepensioneerd
- ☐ Invalide (waardoor persoon niet kan werken)

I4) Wat is/was uw hoofdberoep? [overloop onderstaande mogelijkheden]

- ☐ Huishouden
- ☐ Onderwijs
- ☐ Bediende
- ☐ Kaderpersoneel
- ☐ Zelfstandige (geen vrij beroep)
- ☐ Vrij beroep
- ☐ Arbeider
- ☐ Andere (noteer) ____________________

[**Tip interviewer:** Indien je niet weet waar het beroep thuishoort, schrijf het beroep op bij andere.]

I5) Hoeveel motorvoertuigen (wagens, moto's of brommers,...) die nog werken, zijn er aanwezig in uw huishouden? Gelieve de voertuigen waarvan u geen gebruik maakt OOK mee te rekenen.

[**VERDUIDELIJKING INTERVIEWER:** elektrische fietsen horen hier niet bij]

__ __ (Noteer hier het aantal)

LEES: met behulp van onderstaande vragen kan worden bijgehouden hoe u zich voelt en hoe goed u in staat bent uw gebruikelijke bezigheden uit te voeren. Ik herhaal nog eens dat alles wat u vertelt strikt vertrouwelijk blijft.

I6) Wat vindt u, over het algemeen genomen, van uw gezondheid? [overloop onderstaande mogelijkheden]

- ☐ Uitstekend
- ☐ Zeer goed
- ☐ Goed
- ☐ Matig
- ☐ Slecht

De volgende vragen gaan over dagelijkse bezigheden. Wordt u door uw gezondheid op dit moment beperkt bij deze bezigheden? Zo ja, in welke mate?

I7) Forse inspanningen, zoals hardlopen, zware voorwerpen tillen, inspannend sporten?
[overloop onderstaande mogelijkheden]

- ☐ Ja, ernstig beperkt
- ☐ Ja, een beetje beperkt
- ☐ Nee, helemaal niet beperkt

I8) Een paar trappen lopen?
[overloop onderstaande mogelijkheden]

- ☐ Ja, ernstig beperkt
- ☐ Ja, een beetje beperkt
- ☐ Nee, helemaal niet beperkt

Had u, ten gevolge van uw lichamelijke gezondheid, de afgelopen 4 weken een van de volgende problemen bij uw dagelijkse bezigheden?

I9) Hebt u minder bereikt dan u zou willen?
[overloop onderstaande mogelijkheden]

- ☐ Ja
- ☐ Nee

I10) U was beperkt in het soort bezigheden?
[overloop onderstaande mogelijkheden]

- ☐ Ja
- ☐ Nee

Had u, ten gevolge van een emotioneel probleem (bijvoorbeeld doordat u zich depressief of angstig voelde), de afgelopen 4 weken een van de volgende problemen bij uw dagelijkse bezigheden?

I11) Hebt u minder bereikt dan u zou willen?
[overloop onderstaande mogelijkheden]

- ☐ Ja
- ☐ Nee

I12) Hebt u dingen niet zo zorgvuldig gedaan als u gewend bent?
[overloop onderstaande mogelijkheden]

- ☐ Ja
- ☐ Nee

I13) In welke mate heeft pijn u de afgelopen weken belemmerd bij uw bezigheden?
[overloop onderstaande mogelijkheden]

☐ Helemaal niet ☐ Een klein beetje ☐ Nogal ☐ Veel ☐ Heel erg veel

Deze vragen gaan over hoe u zich de afgelopen 4 weken heeft gevoeld. Wilt u bij elke vraag het antwoord geven dat het beste aansluit bij hoe u zich heeft gevoeld?

I14) Voelde u zich kalm en rustig? [overloop onderstaande mogelijkheden]

☐ Voortdurend ☐ Meestal ☐ Vaak ☐ Soms ☐ Zelden ☐ Nooit

I15) Voelde u zich energiek? [overloop onderstaande mogelijkheden]

☐ Voortdurend ☐ Meestal ☐ Vaak ☐ Soms ☐ Zelden ☐ Nooit

I16) Voelde u zich neerslachtig en somber? [overloop onderstaande mogelijkheden]

☐ Voortdurend ☐ Meestal ☐ Vaak ☐ Soms ☐ Zelden ☐ Nooit

I17) Hoe vaak hebben uw <u>lichamelijke gezondheid of emotionele problemen</u> gedurende de afgelopen 4 weken uw sociale activiteiten (zoals bezoek aan vrienden of familie) belemmerd?
[overloop onderstaande mogelijkheden]

☐ Voortdurend ☐ Meestal ☐ Vaak ☐ Soms ☐ Zelden ☐ Nooit

I18) Hoe vaak heeft u angst om te vallen? [overloop onderstaande mogelijkheden]

☐ Heel vaak ☐ Vaak ☐ Soms ☐ Zelden ☐ Nooit

6171

J) Lichaamsmetingen

1. Lengte: ☐☐☐,☐ cm

2. leeftijd: ☐☐☐ jaar

3. Gewicht: ☐☐☐,☐ kg

4. Lendenomtrek

Meting 1: ☐☐☐,☐ cm Meting 2: ☐☐☐,☐ cm Meting 3: ☐☐☐,☐ cm

5. Handknijpkracht

Meting 1: ☐☐ kg Meting 2: ☐☐ kg

6. Repeated chair stands:

Tijd: ☐☐,☐☐ sec

☐ Deelnemer was niet in staat deze meting uit te voeren

7. Balans:

Moeilijkste stand uitgevoerd	Duur (sec)
☐ Side-by-side	☐☐,☐☐
☐ Semi-tandem	☐☐,☐☐
☐ Volledige tandem	☐☐,☐☐

8. Wandelen 3m

Tijd 1: ☐☐,☐☐ sec

Tijd 2: ☐☐,☐☐ sec

☐ Deelnemer was niet in staat deze meting uit te voeren

LEES:

Dit is het einde van het interview! **Bedankt voor uw deelname aan ons onderzoek**!
Indien u een **cadeaubon** gewonnen heeft, zult u daar persoonlijk van op de hoogte worden gebracht!

K) Bewegingsmeters

OPMERKING: deze pagina enkel voor hoofdonderzoeker (Cedric Busschaert)

Bent u bereid om gedurende 1 week twee kleine bewegingsmetertjes te dragen, zodat we uw mate van beweging in kaart kunnen brengen? Deze bewegingsmeters (gedragen op heup en bovenbeen) hinderen u niet met uw dagelijkse bezigheden. U bent volledig vrij bij uw keuze en dit zal niets veranderen aan de samenwerking met de onderzoekers. Indien u na het dragen van de bewegingsmetertjes graag uw persoonlijke resultaten krijgt, sturen we u deze graag door! Deze meters worden later ook de onderzoekers bij uw thuis opgehaald. (TOON BEWEGINGSMETERS)

- ☐ Ja
- ☐ Neen

Op welk telefoonnummer kunnen we u bereiken?

[**VERDUIDELIJKING INTERVIEWER:** dit nummer zal enkel gebruikt worden bij onduidelijke gegevens. Dit nummer zal nooit worden doorgegeven aan derden.]

Hoofdonderzoeker:

A) geen bewegingsmeters --> doorloop interview vanaf begin

B) wel bewegingsmeters --> start met lichaamsmetingen, dan uitleg meters en afsluiten met het afspreken van nieuwe datum (vervolg interview +/- 60 min & ophalen bewegingsmeters)

C) reeds 100 personen bewegingsmeters gedragen --> doorloop interview vanaf begin
