# Supplementary material for: Reliability and validity of three questionnaires measuring context-specific sedentary behaviour and associated correlates in adolescents, adults and older adults
Source: Int J Behav Nutr Phys Act. 2015 Sep 17;12:117. doi: 10.1186/s12966-015-0277-2 (PMC4574538; doi:10.1186/s12966-015-0277-2)
Supplement: Additional file 4: — Results of the test-retest reliability study of the adolescents: Intraclass Correlation Coefficients (ICC), kappa and percentage agreement (item-specific). (PDF 405 kb) [file 12966_2015_277_MOESM4_ESM.pdf]

Additional file 4 Results of the test-retest reliability study of the adolescents: Intraclass Correlation Coefficients (ICC), kappa and percentage agreement (item-specific)

**Item (per part of the questionnaire)**	**Test-retest reliability**			**Usability**
	**ICC (95% CI)**	**kappa**	**agree**	
**A) Sedentary-related equipment**				
How many of the following non-portable electronic devices do you use and are present at your home?				
Number of TVs	0.95 (0.89; 0.98)	/	85.0	V
Number of DVD players/video players	0.38 (-0.06; 0.70)	/	80.0	V
Number of music players (radio, CD player, stereo,…)	0.87 (0.69; 0.94)	/	70.0	V
Number of computers (desktop)	0.96 (0.89; 0.98)	/	83.3	V
Number of gaming consoles (Xbox, PlayStation,…)	0.95 (0.88; 0.98)	/	78.9	V
How many of these non-portable electronic devices are present in your bedroom?				
Number of TVs	0.72 (0.41; 0.88)	/	95.0	V
Number of DVD players/video players	0.80 (0.56; 0.92)	/	95.0	V
Number of music players (radio, CD player, stereo,…)	0.83 (0.61; 0.93)	/	85.0	V
Number of computers (desktop)	0.66 (0.31; 0.85)	/	95.0	V
Number of gaming consoles (Xbox, PlayStation,…)	0.53 (0.12; 0.78)	/	95.0	V
How many of the following portable electronic devices do you use and are present at your home?				
Number of laptops	0.93 (0.83; 0.97)	/	90.0	V
Number of mobile phones (no smartphone)	0.84 (0.65; 0.94)	/	80.0	V
Number of smartphones	0.84 (0.65; 0.94)	/	70.0	V
Number of music players (iPod, MP3,…)	0.78 (0.51; 0.90)	/	70.0	V
Number of tablets (iPad, Samsung Galaxy Tab,…)	0.87 (0.69; 0.95)	/	89.5	V
Number of gaming consoles (PlayStation portable, Game Boy,…)	0.83 (0.62; 0.93)	/	80.0	V
**B) Potential correlates of TV viewing**				
I think watching TV is pleasant	0.83 (0.62; 0.93)	/	75.0	V
Watching TV takes time away from doing other important things	0.87 (0.70; 0.95)	/	65.0	V
I enjoy watching TV for many hours at a time	0.85 (0.65; 0.94)	/	52.6	V
Watching TV is my way to relax	0.72 (0.41; 0.88)	/	70.0	V
My family members think I spend too much time watching TV	0.64 (0.28; 0.84)	/	55.0	V
I think that I spend too much time watching TV	0.79 (0.54; 0.91)	/	65.0	V
I consider it possible to reduce my TV time	0.49 (0.06; 0.77)	/	57.9	V
I consider it possible to turn off the TV during weekend days until 5:00 p.m.	0.63 (0.27; 0.83)	/	65.0	V
I consider it possible to turn off the TV during meals	0.67 (0.31; 0.86)	/	88.9	V
My family members encourage me to watch less TV	0.63 (0.28; 0.84)	/	65.0	V
My friends encourage me to watch less TV	0.57 (0.17; 0.81)	/	84.2	V
I think that youth my age should not spend more than … (hours/minutes per day) in front of TV	0.72 (0.41; 0.88)	/	55.0	V
In which room do you watch TV most often?	0.63 (0.26; 0.83)	/	90.0	V
How long, on average, do your parents/care givers spend watching TV in leisure time?	0.53 (0.13; 0.78)	/	70.0	V
How long, on average, do your siblings spend watching TV in leisure time?	0.96 (0.90; 0.99)	/	73.7	V
How often do you watch TV with your parents/care givers in leisure time?	0.92 (0.82; 0.97)	/	90.0	V
How often do you watch TV with your siblings in leisure time?	0.92 (0.80; 0.97)	/	80.0	V
To which extent do you consider it possible to implement standing tasks while watching TV (LPA)?	0.84 (0.63; 0.93)	/	60.0	V
To which extent do you consider it possible to replace watching TV for 1 hour by standing tasks with light effort (LPA)?	0.54 (0.14; 0.79)	/	60.0	V
To which extent do you consider it possible to stand up (short bouts) during advertisements while watching TV?	0.59 (0.21; 0.81)	/	55.0	V
To which extent do you consider it possible to stand up for switching the TV station?	0.90 (0.75; 0.96)	/	65.0	V
Do your parents/care givers have rules about how many hours per day you are allowed to watch TV?	/	0.44	88.9	V
The remote controller (TV) can always be found closely to me when I need it	0.85 (0.65; 0.94)	/	75.0	V
The couches at our place are comfortable to sit for a long time	0.83 (0.62; 0.93)	/	70.0	V
**C) Potential correlates of gaming**				
I think playing computer/video games is pleasant	0.74 (0.38; 0.90)	/	66.7	V
I enjoy playing computer/video games for many hours at a time	0.49 (-0.01; 0.80)	/	66.7	V
Playing computer/video games takes time away from doing other important things	0.85 (0.62; 0.95)	/	66.7	V
Playing computer/video games is my way to relax	0.78 (0.45; 0.92)	/	80.0	V
My parents would be pleased if I spent less time playing computer/video games	0.80 (0.50; 0.93)	/	53.3	V
My family members think I spend too much time playing computer/video games	0.59 (0.13; 0.84)	/	53.3	V
I think that I spend too much time playing computer/video games	0.70 (0.31; 0.89)	/	66.7	V
I consider it possible to reduce my time playing computer/video games	0.24 (-0.32; 0.67)	/	57.1	X
My family members encourage me to spend less time playing computer/video games	0.58 (0.11; 0.83)	/	60.0	V
My friends encourage me to spend less time playing computer/video games	0.67 (0.24; 0.88)	/	78.6	V

How long, on average, do your parents/care givers sit when playing computer/video games in leisure time?	0.32 (-0.19; 0.70)	/	81.3	V
How long, on average, do your siblings sit when playing computer/video games in leisure time?	0.92 (0.78; 0.97)	/	56.3	V
How often do you play computer/video games (seated) with your parents/care givers in leisure time?	0.32 (-0.19; 0.70)	/	81.3	V
How often do you play computer/video games (seated) with your siblings in leisure time?	0.88 (0.70; 0.96)	/	75.0	V
How often do you play computer/video games (seated) with your friends in leisure time?	0.77 (0.45; 0.91)	/	75.0	V
To which extent do you consider it possible to stand up while playing computer/video games?	0.24 (-0.28; 0.65)	/	62.5	V
To which extent do you consider it possible to replace playing computer/video games for 1 hour by standing tasks (LPA)?	0.56 (0.10; 0.82)	/	68.8	V
To which extent do you consider it possible to stand up for a couple of minutes after playing computer/video games while sitting for 30 minutes?	0.45 (-0.04; 0.77)	/	50.0	V
Do your parents/care givers have rules about how many hours per day you are allowed to play computer/video games?	/	0.63	92.9	V
**D) Potential correlates of computer use**				
How long, on average, do your parents/care givers sit/lying down when using the computer in leisure time?	0.86 (0.68; 0.94)	/	55.0	V
How long, on average, do your siblings givers sit when using the computer in leisure time?	0.92 (0.80; 0.97)	/	75.0	V
How often do you use the computer (seated) at the same moment with your parents/care givers using a different computer in the same room in leisure time?	0.95 (0.87; 0.98)	/	80.0	V
How often do you use the computer (seated) at the same moment with your siblings using a different computer in the same room in leisure time?	0.84 (0.64; 0.93)	/	70.0	V
To which extent do you consider it possible to stand up while using a computer?	0.87 (0.69; 0.95)	/	65.0	V
To which extent do you consider it possible to replace using a computer for 1 hour by standing tasks (LPA)?	0.47 (0.05; 0.75)	/	60.0	V
To which extent do you consider it possible to stand up for a couple of minutes after using a computer while sitting for 30 minutes?	0.71 (0.39; 0.88)	/	57.9	V
Do your parents/care givers have rules about how many hours per day you are allowed to use a computer?	/	0.49	82.4	V
I think using a computer is pleasant in leisure time	0.82 (0.59; 0.92)	/	70.0	V
Using a computer takes time away from doing other important things	0.78 (0.53; 0.91)	/	80.0	V
I enjoy using a computer for many hours at a time	0.81 (0.58; 0.92)	/	75.0	V
Using a computer is my way to relax	0.80 (0.57; 0.92)	/	75.0	V
My family members think I spend too much time using a computer	0.29 (-0.17; 0.65)	/	57.9	X
I think that I spend too much time using a computer in leisure time	0.16 (-0.30; 0.55)	/	70.0	V
I consider it possible that I do not use a computer for some days in the week (leisure time)	0.50 (0.08; 0.77)	/	45.0	V
I consider it possible to reduce my computer time in leisure time	0.75 (0.47; 0.89)	/	65.0	V
My family members encourage me to spend less time using a computer in leisure time	0.59 (0.21; 0.81)	/	65.0	V
My friends encourage me to spend less time using a computer in leisure time	0.46 (0.03; 0.74)	/	70.0	V
**E) Potential correlates of motorized transport**				
I think using motorized transport is pleasant	0.70 (0.37; 0.87)	/	68.4	V
I think it is pleasant to work (e.g. school-related work, call someone,…) or to rest as a passenger during motorized transport	0.29 (-0.17; 0.64)	/	55.0	X
I feel lazy arriving at my destination after motorized transport	0.86 (0.67; 0.94)	/	68.4	V
I think that I spend too much time using motorized transport	0.73 (0.44; 0.88)	/	70.0	V
I consider it possible to get off the bus/metro spontaneously 1 stop before my destination and to walk the remaining distance	0.55 (0.15; 0.79)	/	50.0	V
I consider it possible to take the bicycle or to go by foot spontaneously even if it is possible to use a bus/metro or ride in a car	0.18 (-0.27; 0.57)	/	55.0	X
My family members encourage me to use (more often) active transport (to bicycle or to walk)	0.61 (0.23; 0.83)	/	63.2	V
My friends encourage me to use (more often) active transport (to bicycle or to walk)	0.57 (0.18; 0.81)	/	75.0	V
My family members think I spend too much time using motorized transport	0.56 (0.17; 0.80)	/	70.0	V
The most chosen transportation possibility to go to work/school from my parents/care givers is …	/	0.30	89.5	V
The most chosen transportation possibility to go to work/school from my siblings is …	/	0.83	90.0	V
The most chosen transportation possibility in leisure time from my parents/care givers is …	/	0.63	85.0	V
The most chosen transportation possibility in leisure time from my siblings is …	/	0.84	90.0	V
To which extent do you consider it possible to stand up spontaneously in a bus, train or metro (instead of sitting)?	0.75 (0.46; 0.89)	/	75.0	V
To which extent do you consider it possible to stand up (for a short duration) after	0.80 (0.57; 0.92)	/	75.0	V

sitting for 30 minutes in bus, train or metro?				
How often do you use motorized transport to and from work/school together with your parents/care givers?	0.93 (0.82; 0.97)	/	80.0	V
How often do you use motorized transport to and from work/school together with your siblings?	0.99 (0.96; 1.00)	/	89.5	V
How often do you use motorized transport together with your parents/care givers in leisure time on a weekday?	0.71 (0.39; 0.87)	/	65.0	V
How often do you use motorized transport together with your parents/care givers in leisure time on a weekend day?	0.72 (0.42; 0.88)	/	70.0	V
How often do you use motorized transport together with your siblings in leisure time on a weekday?	0.91 (0.80; 0.97)	/	80.0	V
How often do you use motorized transport together with your siblings in leisure time on a weekend day?	0.89 (0.73; 0.96)	/	66.7	V
**F) Potential correlates of school**				
How many hours physical education or sport do you have during the week at school (= during classes; include 'swimming classes'; do not include 'after school activities')	0.92 (0.81; 0.97)	/	85.0	V
What is your most common behaviour during school breaks at school?	0.57 (0.18; 0.80)	/	75.0	V
What is your most common behaviour during lunch break at school?	0.79 (0.55; 0.91)	/	75.0	V
**G) Sedentary behaviours**				
In the last 7 days, on average, how long did you spend sitting or lying down watching TV in leisure time per weekday? (do include meals while sitting and watching TV)	0.79 (0.54; 0.91)	/	45.0	V
In the last 7 days, on average, how long did you spend sitting or lying down watching TV in leisure time per weekend day? (do include meals while sitting and watching TV)	0.65 (0.31; 0.85)	/	55.0	V
In the last 7 days, on average, how long did you spend sitting or lying down during playing computer/video games in leisure time per weekday?	0.10 (-0.40; 0.56)	/	18.8	X
In the last 7 days, on average, how long did you spend sitting or lying down during playing computer/video games in leisure time per weekend day?	0.36 (-0.17; 0.73)	/	53.3	X
In the last 7 days, on average, how long did you spend sitting or lying down during computer use in leisure time per weekday?	0.50 (0.09; 0.77)	/	40.0	V
In the last 7 days, on average, how long did you spend sitting or lying down during computer use in leisure time per weekend day?	0.50 (0.07; 0.77)	/	42.1	V
In the last 7 days, on average, how long did you sit a day while using motorized transport to and from your school (in a car, bus, train, on a motorbike, etc.; do not include cycling on a pedal bicycle)?	0.86 (0.66; 0.95)	/	52.9	V
In the last 7 days, on average, how long did you sit using motorized transport in leisure time (e.g. to shops, friends,…), apart from your transport to and from school, per weekday (in a car, bus, train, on a motorbike, etc.; do not include cycling on a pedal bicycle)?	0.33 (-0.14; 0.67)	/	57.9	X
In the last 7 days, on average, how long did you sit using motorized transport in leisure time (e.g. to shops, friends,…), apart from your transport to and from school, per weekend day (in a car, bus, train, on a motorbike, etc.; do not include cycling on a pedal bicycle)?	-0.06 (-0.49; 0.40)	/	26.3	X
In the last 7 days, on average, how long did you spend sitting during classes at school per day (do not include study time at home)?	-0.01 (-0.44; 0.43)	/	35.0	X
In the last 7 days, on average, how long did you spend sitting or lying down during the following activities per day?				
Sitting while reading (book, magazine,…) on a weekday – only printed documents	0.76 (0.48; 0.90)	/	65.0	V
Sitting while reading (book, magazine,…) on a weekend day – only printed documents	0.92 (0.80; 0.97)	/	73.7	V
Sitting for hobbies (voluntary work, drawing classes,…) on a weekday	0.19 (-0.27; 0.57)	/	70.0	V
Sitting for hobbies (voluntary work, drawing classes,…) on a weekend day	0.18 (-0.27; 0.57)	/	70.0	V
Sitting for socializing (visiting friends, cinema,…) on a weekday	0.55 (0.15; 0.79)	/	25.0	V
Sitting for socializing (visiting friends, cinema,…) on a weekend day	0.43 (0.001;0.73)	/	55.0	V
Sitting while listening to music (radio, MP3,…) on a weekday	0.48 (0.02; 0.77)	/	44.4	V
Sitting while listening to music (radio, MP3,…) on a weekend day	0.38 (-0.09; 0.71)	/	33.3	X
Sitting during meals (breakfast – dinner) on a weekday – do NOT include meals while sitting and watching TV	0.13 (-0.32; 0.53)	/	55.0	X
Sitting during meals (breakfast – dinner) on a weekend day – do NOT include meals while sitting and watching TV	0.03 (-0.41; 0.46)	/	55.0	X
Sitting during school work (at home: making homework, studying, using the computer for school,…) on a weekday	0.52 (0.11; 0.78)	/	60.0	V
Sitting during school work (at home: making homework, studying, using the computer for school,…) on a weekend day	0.45 (0.02; 0.74)	/	30.0	V
Sitting for using mobile phone (calling and texting) on a weekday	0.40 (-0.04; 0.71)	/	50.0	X
Sitting for using mobile phone (calling and texting) on a weekend day	0.49 (0.08; 0.76)	/	50.0	V
**H) Simultaneous behaviour**				
How often do you do the following situations at the same time (simultaneously)?				
Watching TV AND using mobile phone	0.87 (0.71; 0.95)	/	70.0	V
Watching TV AND using computer/tablet	0.85 (0.67; 0.94)	/	65.0	V

Using computer AND using mobile phone	0.71 (0.39; 0.88)	/	63.2	V
Using computer AND listening to music	0.71 (0.38; 0.88)	/	68.4	V
Using mobile phone (texting) AND having conversation with friends/family	0.88 (0.72; 0.95)	/	70.0	V
Using mobile phone AND listening to music	0.83 (0.61; 0.93)	/	75.0	V

Note: Agree (% agreement), LPA (light physical activities), CI (confidence interval).
Interpretation 'usability': V (item has moderate-to-excellent reliability, so can be used as a reliable item); X (item showed to have poor reliability, so cannot be used as a reliable single item)
