# Supplementary material for: Reliability and validity of three questionnaires measuring context-specific sedentary behaviour and associated correlates in adolescents, adults and older adults
Source: Int J Behav Nutr Phys Act. 2015 Sep 17;12:117. doi: 10.1186/s12966-015-0277-2 (PMC4574538; doi:10.1186/s12966-015-0277-2)
Supplement: Additional file 5: — Results of the test-retest reliability study of the adults: Intraclass Correlation Coefficients (ICC), kappa and percentage agreement (item-specific). (PDF 424 kb) [file 12966_2015_277_MOESM5_ESM.pdf]

**Additional file 5** Results of the test-retest reliability study of the adults: Intraclass Correlation Coefficients (ICC), kappa and percentage agreement (item-specific)

| Item (per part of the questionnaire) | Test-retest reliability | | | Usability |
|---|---|---|---|---|
| | ICC (95% CI) | kappa | agree | |
| **A) Potential correlates of TV viewing** | | | | |
| I think watching TV is pleasant | 0.75 (0.49; 0.89) | / | 59.1 | V |
| Watching TV takes time away from doing other important things | 0.67 (0.35; 0.85) | / | 50.0 | V |
| I enjoy watching TV for many hours at a time | 0.73 (0.46; 0.88) | / | 72.7 | V |
| Watching TV is my way to relax after a school day/workday | 0.67 (0.36; 0.85) | / | 45.5 | V |
| My family members think I spend too much time watching TV | 0.87 (0.71; 0.95) | / | 81.0 | V |
| I think that I spend too much time watching TV | 0.82 (0.62; 0.92) | / | 81.8 | V |
| I consider it possible to reduce my TV time | 0.31 (-0.14; 0.66) | / | 35.0 | X |
| I consider it possible to turn off the TV during weekend days until 5:00 p.m. | 0.57 (0.21; 0.80) | / | 81.8 | V |
| I consider it possible to turn off the TV during meals | 0.74 (0.47; 0.88) | / | 81.8 | V |
| My family members encourage me to watch less TV | 0.38 (-0.05; 0.69) | / | 76.2 | V |
| My friends encourage me to watch less TV | 0.59 (0.23; 0.81) | / | 52.4 | V |
| I think that adults should not spend more than … (hours/minutes per day) in front of TV | 0.76 (0.49; 0.89) | / | 57.1 | V |
| In which room do you watch TV most often? | 0.35 (-0.07; 0.67) | / | 95.5 | V |
| How long, on average, does your partner spend watching TV? | 0.84 (0.65; 0.93) | / | 47.6 | V |
| How long, on average, do your children (still living at home) spend watching TV? | 0.88 (0.69; 0.96) | / | 50.0 | V |
| How often do you watch TV with your partner? | 0.88 (0.73; 0.95) | / | 71.4 | V |
| How often do you watch TV with your children (still living at home)? | 0.93 (0.80; 0.98) | / | 66.7 | V |
| To which extent do you consider it possible to implement standing tasks while watching TV (e.g. ironing, doing chores)? | 0.55 (0.17; 0.79) | / | 42.9 | V |
| To which extent do you consider it possible to replace watching TV for 1 hour by standing tasks with light effort (e.g. cleaning, doing chores)? | 0.57 (0.20; 0.80) | / | 28.6 | V |
| To which extent do you consider it possible to stand up (short bouts) during advertisements while watching TV? | 0.43 (0.003; 0.72) | / | 47.6 | V |
| **B) Potential correlates of computer use** | | | | |
| How long, on average, does your partner sit when using the computer (tablet, internet on smartphone, laptop, desktop,…) in leisure time? | 0.20 (-0.26; 0.58) | / | 25.0 | X |
| How long, on average, do your children (still living at home) sit when using the computer (tablet, internet on smartphone, laptop, desktop,…) in leisure time? | 0.83 (0.60; 0.93) | / | 52.6 | V |
| How often do you use the computer (seated) at the same moment with your partner using a different computer in the same room in leisure time? | 0.67 (0.33; 0.85) | / | 40.0 | V |
| How often do you use the computer (seated) at the same moment with your children (still living at home) using a different computer in the same room in leisure time? | 0.92 (0.79; 0.97) | / | 73.3 | V |
| To which extent do you consider it possible to stand up while using a computer? | 0.34 (-0.09; 0.67) | / | 52.4 | X |
| To which extent do you consider it possible to replace using a computer for 1 hour by standing tasks with light effort (e.g. cleaning, doing chores)? | 0.43 (0.01; 0.72) | / | 33.3 | V |
| To which extent do you consider it possible to stand up for a couple of minutes after using a computer while sitting for 30 minutes? | 0.18 (-0.26; 0.56) | / | 52.4 | X |
| I think using a computer is pleasant in leisure time | 0.75 (0.49; 0.89) | / | 47.6 | V |
| Using a computer takes time away from doing other important things | 0.59 (0.21; 0.81) | / | 55.0 | V |
| I enjoy using a computer for many hours at a time | 0.79 (0.56; 0.91) | / | 76.2 | V |
| Using a computer is my way to relax after a school day/workday | 0.66 (0.33; 0.85) | / | 52.4 | V |
| My family members think I spend too much time using a computer | 0.35 (-0.09; 0.67) | / | 57.1 | X |
| I think that I spend too much time using a computer | 0.55 (0.15; 0.79) | / | 60.0 | V |
| I consider it possible that I do not use a computer for some days in the week (leisure time) | 0.55 (0.17; 0.79) | / | 52.4 | V |
| I consider it possible to reduce my computer time in leisure time | 0.70 (0.39; 0.86) | / | 61.9 | V |
| My family members encourage me to spend less time using a computer in leisure time | 0.38 (-0.06; 0.70) | / | 60.0 | X |
| My friends encourage me to spend less time using a computer in leisure time | 0.52 (0.12; 0.77) | / | 52.4 | V |
| **C) Potential correlates of motorized transport** | | | | |
| I feel lazy arriving at my destination after motorized transport | 0.43 (0.004; 0.72) | / | 52.4 | V |
| I think using motorized transport is pleasant | 0.72 (0.43; 0.88) | / | 60.0 | V |
| I think it is pleasant to work or to rest as a passenger during motorized transport | 0.58 (0.20; 0.81) | / | 45.0 | V |
| I think that I spend too much time using motorized transport | 0.66 (0.33; 0.85) | / | 61.9 | V |
| I consider it possible to get off the bus/metro spontaneously 1 stop before my destination and to walk the remaining distance | 0.67 (0.30; 0.86) | / | 55.6 | V |
| I consider it possible to park the car somewhat further spontaneously and to walk the remaining distance | 0.66 (0.32; 0.84) | / | 38.1 | V |
| I consider it possible to take the bicycle or to go by foot spontaneously even if it is possible to use a car | 0.75 (0.47; 0.89) | / | 61.9 | V |
| My family members encourage me to use (more often) active transport (to bicycle or to walk) | 0.45 (0.03; 73) | / | 38.1 | V |

| | | | | |
|---|---|---|---|---|
| My friends encourage me to use (more often) active transport (to bicycle or to walk) | 0.55 (0.16; 0.79) | / | 42.9 | V |
| My family members think I spend too much time using motorized transport | 0.50 (0.10; 0.76) | / | 61.9 | V |
| The most chosen transportation possibility to go to work/school from my partner is … | / | 1.00 | 100 | V |
| The most chosen transportation possibility to go to work/school from my children (still living at home) is … | / | 0.59 | 73.7 | V |
| The most chosen transportation possibility in leisure time from my partner is … | / | 0.29 | 75.0 | V |
| The most chosen transportation possibility in leisure time from my children (still living at home) is … | / | 0.75 | 84.2 | V |
| To which extent do you consider it possible to stand up spontaneously in a bus, train or metro (instead of sitting)? | 0.78 (0.52; 0.91) | / | 50.0 | V |
| To which extent do you consider it possible to stand up (for a short duration) after sitting for 30 minutes in bus, train or metro? | 0.72 (0.41; 0.88) | / | 55.0 | V |
| It is easy to park the car at public places (shops, station,…) | 0.70 (0.40; 0.87) | / | 66.7 | V |
| I receive a lot of information about 'sitting activities' like cinema or sport games via billboards along the road and/or the radio | 0.63 (0.27; 0.83) | / | 55.0 | V |
| How often do you use motorized transport to and from work/school together with your partner? | 0.87 (0.70; 0.94) | / | 81.0 | V |
| How often do you use motorized transport to and from work/school together with your children (still living at home)? | 0.81 (0.58; 0.92) | / | 76.2 | V |
| How often do you use motorized transport in leisure time together with your partner on a weekday? | 0.77 (0.51; 0.90) | / | 57.1 | V |
| How often do you use motorized transport in leisure time together with your partner on a weekend day? | 0.42 (-0.004; 0.72) | / | 71.4 | V |
| How often do you use motorized transport in leisure time together with your children (still living at home) on a weekday? | 0.94 (0.86; 0.98) | / | 80.0 | V |
| How often do you use motorized transport in leisure time together with your children (still living at home) on a weekend day? | 1.00 (1.00; 1.00) | / | 100 | V |
| **D) Potential correlates of occupation** | | | | |
| My attention decreases while sitting for a long time | 0.63 (0.27; 0.83) | / | 35.0 | V |
| I think it is pleasant to sit for a long time during working hours | 0.52 (0.11; 0.78) | / | 50.0 | V |
| Sitting at work is an ideal opportunity to rest | 0.73 (0.43; 0.88) | / | 75.0 | V |
| My colleagues think I spend too much time sitting | 0.60 (0.19; 0.83) | / | 44.4 | V |
| I think that I spend too much time sitting at work | 0.72 (0.41; 0.88) | / | 57.9 | V |
| I consider it possible to do certain tasks (calling,…) while standing | 0.66 (0.31; 0.85) | / | 60.0 | V |
| I consider it possible to stand up for a while after a period of uninterrupted sitting | 0.54 (0.14; 0.79) | / | 55.0 | V |
| I consider it possible to stand up more often during breaks | 0.48 (0.07; 0.76) | / | 55.0 | V |
| My colleagues encourage me to sit less | 0.31 (-0.15; 0.65) | / | 55.0 | X |
| My colleagues at my office and/or department stand up regularly during working hours and/or breaks | 0.50 (0.08; 0.76) | / | 45.0 | V |
| What action do you do most often when you have a question for a colleague at another office? | 0.87 (0.70; 0.95) | / | 94.7 | V |
| How many persons are there in your office (including yourself)? | 0.94 (0.81; 0.98) | / | 76.9 | V |
| At work I have bicycles provided by the employer | / | 0.69 | 90.0 | V |
| At work I have employer subsidized public transport | / | 0.77 | 90.0 | V |
| The office chairs are comfortable to sit | / | 0.35 | 70.6 | V |
| There is a spacious and/or easy parking | / | 0.44 | 89.5 | V |
| There is a bicycle parking | / | 0.73 | 89.5 | V |
| There are standing desks (heightened desks at which one can work while standing) | / | 1.00 | 100 | V |
| To which extent do you consider it possible to stand up while working at your desk? | 0.74 (0.40; 0.90) | / | 68.8 | V |
| To which extent do you consider it possible to stand up while calling? | 0.55 (0.13; 0.81) | / | 44.4 | V |
| To which extent do you consider it possible to go to a colleague yourself instead of calling him/her? | 0.40 (-0.09; 0.73) | / | 58.8 | X |
| To which extent do you consider it possible to stand up for a couple of minutes after sitting for 30 minutes? | 0.63 (0.24; 0.84) | / | 72.2 | V |
| To which extent do you consider it possible to remove appliances so that you would have to stand up to operate them (e.g. printer, telephone,…)? | 0.49 (-0.01; 0.80) | / | 53.3 | V |
| I think interrupting sitting activities by standing up at work is disturbing for colleagues | 0.78 (0.50; 0.92) | / | 52.9 | V |
| I think interrupting sitting activities by standing up at work is ridiculous | 0.06 (-0.44; 52) | / | 25.0 | X |
| I think interrupting sitting activities by standing up at work is good for my health | 0.76 (0.42; 0.91) | / | 73.3 | V |
| I am sure that I can stand up (more often) as a break during my work, even if my colleagues are not doing this | 0.39 (-0.10; 0.72) | / | 35.3 | X |
| I am sure that I can stand up (more often) as a break during my work, even if my colleagues do not support me | 0.51 (0.04; 0.80) | / | 62.5 | V |
| How would you like to perform the following tasks at work? | | | | |
| Making phone calls | / | 0.74 | 88.2 | V |
| Having short meetings | / | 0.78 | 88.9 | V |

| | | | | |
|---|---|---|---|---|
| Doing desk work | / | Δ | 94.7 | V |
| Taking coffee breaks | / | 0.89 | 95.0 | V |
| Taking lunch breaks | / | -0.07 | 85.0 | V |
| **E) Potential correlates of household tasks and making phone calls** | | | | |
| I think it is pleasant to sit while doing household tasks | 0.66 (0.34; 0.84) | / | 45.5 | V |
| I work faster when I sit during household tasks | 0.63 (0.29; 0.83) | / | 45.5 | V |
| I would like to stand more often while doing household tasks | 0.38 (-0.04; 0.68) | / | 54.5 | X |
| I think that I can stand up more often while doing household tasks | 0.54 (0.15; 0.79) | / | 50.0 | V |
| I consider it possible to stand up while calling | 0.74 (0.46; 0.88) | / | 68.2 | V |
| I consider it possible to stand up while doing tasks in the kitchen | 0.76 (0.51; 0.89) | / | 81.8 | V |
| My family members encourage me to sit less while doing household tasks | 0.27 (-0.17; 0.61) | / | 45.5 | X |
| Household tasks are usually done while sitting by my children | 0.85 (0.68; 0.94) | / | 72.7 | V |
| Household tasks are usually done while sitting by my partner | 0.26 (-0.17; 0.61) | / | 40.9 | X |
| How would you like to perform the following household tasks? | | | | |
| Making phone calls | / | 0.70 | 86.4 | V |
| Peeling potatoes/cutting vegetables | / | 0.61 | 90.9 | V |
| Ironing | / | Δ | 95.5 | V |
| Folding laundry | / | 0.49 | 86.4 | V |
| Brushing teeth | / | Δ | 100 | V |
| To repair something | / | 0.31 | 76.2 | V |
| To which extent do you consider it possible to do more household tasks while being upright? | 0.55 (0.18; 0.79) | / | 45.5 | V |
| To which extent do you consider it possible to remove appliances so that you would have to stand up to operate them (e.g. printer, remote controller TV,…)? | 0.62 (0.27; 0.82) | / | 50.0 | V |
| I am sure that I can interrupt my sitting activities (more often) at home, even if my family members are not doing this | 0.61 (0.26; 0.81) | / | 63.6 | V |
| I am sure that I can interrupt my sitting activities (more often) at home, even if my family members do not support/understand me | 0.56 (0.17; 0.80) | / | 50.0 | V |
| **F) Sedentary-related equipment** | | | | |
| How many of the following non-portable electronic devices do you use and are present at your home? | | | | |
| Number of TVs (pay TV) | 0.86 (0.69; 0.94) | / | 90.9 | V |
| Number of TVs (no pay TV) | 0.77 (0.52; 0.90) | / | 81.8 | V |
| Number of DVD players/video players | 0.86 (0.69; 0.94) | / | 86.4 | V |
| Number of music players (radio, CD player, stereo,…) | 0.74 (0.46; 0.89) | / | 47.6 | V |
| Number of computers (desktop) | 0.76 (0.50; 0.89) | / | 81.8 | V |
| Number of gaming consoles for TV (Xbox, PlayStation,…) | 0.67 (0.35; 0.85) | / | 85.7 | V |
| Number of telephones (dedicated line) | 0.91 (0.80; 0.96) | / | 86.4 | V |
| How many of these non-portable electronic devices are present in your bedroom? | | | | |
| Number of TVs (pay TV) | 1.00 (1.00; 1.00) | / | 100 | V |
| Number of TVs (no pay TV) | 1.00 (1.00; 1.00) | / | 100 | V |
| Number of DVD players/video players | 1.00 (1.00; 1.00) | / | 100 | V |
| Number of music players (radio, CD player, stereo,…) | 0.70 (0.40; 0.86) | / | 86.4 | V |
| Number of computers (desktop) | ^ | / | 95.2 | V |
| Number of gaming consoles for TV (Xbox, PlayStation,…) | ^ | / | 100 | V |
| Number of telephones (dedicated line) | 0.84 (0.65; 0.93) | / | 95.5 | V |
| How many of the following portable electronic devices do you use and are present at your home? | | | | |
| Number of laptops | 0.29 (-0.14; 0.63) | / | 68.2 | V |
| Number of mobile phones (no smartphone) | 0.49 (0.10; 0.75) | / | 77.3 | V |
| Number of smartphones | 0.87 (0.72; 0.95) | / | 86.4 | V |
| Number of music players (iPod, MP3,…) | 0.51 (0.12; 0.76) | / | 90.9 | V |
| Number of gaming consoles (PlayStation portable, Game Boy,…) | 0.31 (-0.13; 0.65) | / | 95.2 | V |
| Number of tablets (iPad, Samsung Galaxy Tab,…) | 0.89 (0.76; 0.96) | / | 95.2 | V |
| The remote controller (TV) can always be found closely to me when I need it | 0.50 (0.11; 0.76) | / | 63.6 | V |
| The couches at our place are comfortable to sit for a long time | 0.56 (0.19; 0.79) | / | 68.2 | V |
| **G) Sedentary behaviours** | | | | |
| In the last 7 days, on average, how long did you spend sitting or lying down watching TV in leisure time per weekday? (do include meals while sitting and watching TV) | 0.77 (0.50; 0.90) | / | 45.0 | V |
| In the last 7 days, on average, how long did you spend sitting or lying down watching TV in leisure time per weekend day? (do include meals while sitting and watching TV) | 0.73 (0.44; 0.88) | / | 25.0 | V |
| In the last 7 days, on average, how long did you spend sitting during computer use in leisure time per weekday? | 0.89 (0.75; 0.95) | / | 71.4 | V |
| In the last 7 days, on average, how long did you spend sitting during computer use in leisure time per weekend day? | 0.90 (0.76; 0.96) | / | 60.0 | V |

| | | | | |
|---|---|---|---|---|
| In the last 7 days, on average, how long did you sit a day while travelling to and from your occupation/school (moped, in a car, bus, train, metro or on a motorbike; do not include cycling on a pedal bicycle)? | 0.95 (0.88; 0.98) | / | 68.4 | V |
| In the last 7 days, on average, how long did you sit a day while travelling as part of your occupation (during working hours) (moped, in a car, bus, train, metro or on a motorbike; do not include cycling on a pedal bicycle)? | 0.86 (0.66; 0.94) | / | 52.6 | V |
| In the last 7 days, on average, how long did you sit while travelling in leisure time (e.g. to shops, friends,...), apart from your transport to and from occupation/school, per weekday (in a car, bus, train, metro, on a motorbike, moped; do not include cycling on a pedal bicycle)? | 0.73 (0.45; 0.88) | / | 38.1 | V |
| In the last 7 days, on average, how long did you sit while travelling in leisure time (e.g. to shops, friends,...), apart from your transport to and from occupation/school, per weekend day (in a car, bus, train, metro, on a motorbike, moped; do not include cycling on a pedal bicycle)? | 0.22 (-0.24; 0.61) | / | 31.6 | X |
| In the last 7 days, on average, how much time per day did you spend sitting while doing your occupation? | 0.88 (0.73; 0.95) | / | 50.0 | V |
| In the last 7 days, on average, how much time per day did you spend sitting while doing household tasks (e.g. cooking, ironing, to repair something) and making phone calls at home per weekday? | 0.49 (0.09; 0.75) | / | 45.5 | V |
| In the last 7 days, on average, how much time per day did you spend sitting while doing household tasks (e.g. cooking, ironing, to repair something) and making phone calls at home per weekend day? | 0.45 (0.05; 0.73) | / | 40.9 | V |
| In the last 7 days, on average, how long did you spend sitting or lying down during the following activities per day? | | | | |
| Sitting while reading (book, magazine, newspaper,…) on a weekday | 0.53 (0.14; 0.78) | / | 42.9 | V |
| Sitting while reading (book, magazine, newspaper,…) on a weekend day | 0.63 (0.28; 0.83) | / | 38.1 | V |
| Sitting while caring (children, family members,…) on a weekday | 0.51 (0.11; 0.77) | / | 66.7 | V |
| Sitting while caring (children, family members,…) on a weekend day | 0.68 (0.34; 0.86) | / | 68.4 | V |
| Sitting for hobbies (drawing, playing cards,…) on a weekday | 0.67 (0.35; 0.85) | / | 38.1 | V |
| Sitting for hobbies (drawing, playing cards,…) on a weekend day | 0.49 (0.07; 0.76) | / | 40.0 | V |
| Sitting for socializing (visiting friends, in a pub, cinema,…) on a weekday | 0.44 (0.02; 0.73) | / | 47.6 | V |
| Sitting for socializing (visiting friends, in a pub, cinema,…) on a weekend day | 0.81 (0.59; 0.92) | / | 42.9 | V |
| Sitting while listening to music (radio, CD, iPod,…) on a weekday | 0.74 (0.47; 0.88) | / | 45.5 | V |
| Sitting while listening to music (radio, CD, iPod,…) on a weekend day | 0.69 (0.38; 0.86) | / | 57.1 | V |
| Sitting during meals (breakfast,...) on a weekday | 0.66 (0.33; 0.84) | / | 68.2 | V |
| Sitting during meals (breakfast,...) on a weekend day | 0.63 (0.28; 0.83) | / | 52.4 | V |
| **H) Simultaneous behaviour** | | | | |
| How often do you do the following situations at the same time? | | | | |
| Watching TV AND using mobile phone | 0.87 (0.70; 0.94) | / | 66.7 | V |
| Watching TV AND using computer/tablet | 0.87 (0.71; 0.95) | / | 71.4 | V |
| Using computer AND using mobile phone | 0.86 (0.70; 0.94) | / | 68.2 | V |
| Using computer AND listening to music | 0.69 (0.39; 0.86) | / | 54.5 | V |
| Using mobile phone AND having conversation with friends/family | 0.61 (0.26; 0.82) | / | 59.1 | V |
| Using mobile phone AND listening to music | 0.67 (0.36; 0.85) | / | 68.2 | V |

*Note:* ^ (scale has zero variance items), Δ (*At least one variable in test-retest analyses is a constant). Agree (% agreement), LPA (light physical activities), CI (confidence interval). Interpretation 'usability': V (item has moderate-to-excellent reliability, so can be used as a reliable item ); X (item showed to have poor reliability, so cannot be used as a reliable single item)*
