# Supplementary material for: Reliability and validity of three questionnaires measuring context-specific sedentary behaviour and associated correlates in adolescents, adults and older adults
Source: Int J Behav Nutr Phys Act. 2015 Sep 17;12:117. doi: 10.1186/s12966-015-0277-2 (PMC4574538; doi:10.1186/s12966-015-0277-2)
Supplement: Additional file 6: — Results of the test-retest reliability study of the older adults: Intraclass Correlation Coefficients (ICC), kappa and percentage agreement (item-specific). (PDF 395 kb) [file 12966_2015_277_MOESM6_ESM.pdf]

Additional file 6 Results of the test-retest reliability study of the older adults: Intraclass Correlation Coefficients (ICC), kappa and percentage agreement (item-specific)

Item (per part of the questionnaire)	Test-retest reliability			Usability
	ICC (95% CI)	kappa	agree	
**A) Potential correlates of TV viewing**				
I think watching TV is pleasant	0.69 (0.36; 0.86)	/	60.0	V
Watching TV takes time away from doing other important things	0.28 (-0.18; 0.63)	/	70.0	V
I enjoy watching TV for many hours at a time	0.79 (0.55; 0.91)	/	50.0	V
Watching TV is my way to relax	0.43 (0.001;0.73)	/	45.0	V
My partner thinks I spend too much time watching TV	0.00 (-0.47; 0.47)	/	82.4	V
I think that I spend too much time watching TV	0.34 (-0.12; 0.68)	/	57.9	X
I consider it possible to reduce my TV time	0.44 (0.01; 0.73)	/	35.0	V
I consider it possible to turn off the TV during weekend days until 5:00 p.m.	0.88 (0.72; 0.95)	/	70.0	V
I consider it possible to turn off the TV during meals	-0.06 (-0.48;0.39)	/	80.0	V
My partner encourages me to watch less TV	0.00 (-0.47; 0.47)	/	88.2	V
My friends encourage me to watch less TV	0.00 (-0.43; 0.43)	/	90.0	V
I think that people my age should not spend more than … (hours/minutes per day) in front of TV	0.82 (0.60; 0.93)	/	75.0	V
In which room do you watch TV most often?	1.00 (1.00; 1.00)	/	100	V
How long, on average, does your partner spend watching TV?	0.94 (0.83; 0.98)	/	76.5	V
How often do you watch TV with your partner?	0.57 (0.14; 0.82)	/	52.9	V
To which extent do you consider it possible to implement standing tasks while watching TV (e.g. ironing, doing chores, playing with (grand)children - light effort)?	-0.19 (-0.57;0.27)	/	65.0	V
To which extent do you consider it possible to replace watching TV for 1 hour by tasks with light effort (e.g. cleaning, doing chores)?	0.00 (-0.43; 0.43)	/	35.0	X
To which extent do you consider it possible to stand up (short bouts) during advertisements while watching TV?	-0.02 (-0.45;0.42)	/	75.0	V
To which extent do you consider it possible to stand up to switch TV station?	0.51 (0.11; 0.78)	/	45.0	V
**B) Potential correlates of computer use**				
How long, on average, does your partner sit when using the computer (tablet, internet on smartphone, laptop, desktop,…)?	0.90 (0.74; 0.97)	/	60.0	V
How often do you use the computer (seated) at the same moment with your partner using a different computer in the same room?	0.93 (0.79; 0.97)	/	66.7	V
To which extent do you consider it possible to stand up while using a computer?	0.92 (0.79; 0.97)	/	81.3	V
To which extent do you consider it possible to replace using a computer for 1 hour by tasks with light effort (e.g. cleaning, doing chores)?	0.24 (-0.32; 0.67)	/	35.7	X
To which extent do you consider it possible to stand up for a couple of minutes after using a computer while sitting for 30 minutes?	0.03 (-0.46; 0.50)	/	56.3	X
I think using a computer is pleasant	0.76 (0.45; 0.91)	/	56.3	V
Using a computer takes time away from doing other important things	0.27 (-0.25; 0.66)	/	68.8	V
I enjoy using a computer for many hours at a time	0.92 (0.78; 0.97)	/	62.5	V
Using a computer is my way to relax	0.63 (0.21; 0.85)	/	31.3	V
My partner thinks I spend too much time using a computer	0.76 (0.42; 0.91)	/	73.3	V
I think that I spend too much time using a computer	0.49 (0.004;0.78)	/	62.5	V
I consider it possible that I do not use a computer for some days in the week	0.44 (-0.06; 0.76)	/	25.0	V
I consider it possible to reduce my computer time	0.34 (-0.17; 0.71)	/	31.3	X
My partner encourages me to spend less time using a computer	0.53 (0.05; 0.81)	/	66.7	V
My friends encourage me to spend less time using a computer	-0.15 (-0.59;0.36)	/	62.5	V
**C) Potential correlates of motorized transport**				
I think using motorized transport is pleasant	0.48 (0.06; 0.76)	/	45.0	V
I think it is pleasant to work or to rest (e.g. drawing, making phone calls,…) as a passenger during motorized transport	0.05 (-0.39; 0.47)	/	60.0	X
I feel lazy arriving at my destination after motorized transport	0.19 (-0.27; 0.57)	/	65.0	V
I think that I spend too much time using motorized transport	0.13 (-0.33; 0.53)	/	70.0	V
I consider it possible to get off the bus/metro spontaneously 1 stop before my destination and to walk the remaining distance	0.66 (0.31; 0.85)	/	60.0	V
I consider it possible to park the car somewhat further spontaneously and to walk the remaining distance	0.58 (0.19; 0.81)	/	60.0	V
I consider it possible to take the bicycle or to go by foot spontaneously even if it is possible to use a car	0.50 (0.9; 0.77)	/	65.0	V
My partner encourages me to use (more often) active transport (to bicycle or to walk)	0.74 (0.41; 0.90)	/	70.6	V
My friends encourage me to use (more often) active transport (to bicycle or to walk)	0.04 (-0.40; 0.46)	/	55.0	X
My partner thinks I spend too much time using motorized transport	-0.17 (-0.59;0.32)	/	64.7	V
The most chosen transportation possibility from my partner is …	/	0.51	76.5	V
To which extent do you consider it possible to stand up spontaneously in a bus, train or metro (instead of sitting)?	0.76 (0.49; 0.90)	/	75.0	V
To which extent do you consider it possible to stand up (for a short duration) after sitting for 30 minutes in bus, train or metro?	0.73 (0.43; 0.88)	/	70.0	V
It is easy to park the car at public places (shops, station,…)	0.91 (0.78; 0.96)	/	80.0	V

I receive a lot of information about 'sitting activities' like cinema/theatre or sport games via billboards along the road and/or the radio	0.04 (-0.40; 0.47)	/	45.0	X
How often do you use motorized transport together with your partner per weekday?	0.23 (-0.27; 0.63)	/	29.4	X
How often do you use motorized transport together with your partner per weekend day?	-0.20 (-0.61;0.30)	/	47.1	X
**D) Potential correlates of household tasks and making phone calls**				
I think it is pleasant to sit while doing household tasks	0.75 (0.47; 0.89)	/	75.0	V
I work faster when I sit during household tasks	0.54 (0.14; 0.79)	/	80.0	V
I would like to stand more often while doing household tasks	0.52 (0.11; 0.78)	/	85.0	V
I think that I can stand up more often while doing household tasks	0.42 (-0.02; 0.72)	/	80.0	V
I consider it possible to stand up while calling	0.48 (0.06; 0.76)	/	65.0	V
I consider it possible to stand up while doing tasks in the kitchen	0.88 (0.73; 0.95)	/	90.0	V
My partner encourages me to sit less while doing household tasks	0.73 (0.40; 0.89)	/	82.4	V
Household tasks (and calling) are usually done while sitting by my partner	0.66 (0.28; 0.86)	/	76.5	V
How would you like to perform the following household tasks?				
Making phone calls	/	0.89	95.0	V
Peeling potatoes/cutting vegetables	/	0.39	80.0	V
Ironing	/	1.00	100	V
Folding laundry	/	1.00	100	V
Brushing teeth	/	Δ	100	V
To repair something small	/	0.38	80.0	V
To which extent do you consider it possible to do more household tasks while being upright?	0.65 (0.30; 0.84)	/	90.0	V
To which extent do you consider it possible to remove appliances so that you would have to stand up to operate them (e.g. printer, remote controller TV,…)?	0.67 (0.33; 0.85)	/	65.0	V
I am sure that I can interrupt my sitting activities (more often) at home, even if my partner is not doing this	-0.14 (-0.57;0.35)	/	64.7	V
I am sure that I can interrupt my sitting activities (more often) at home, even if my partner do not support/understand me	-0.19 (-0.61;0.30)	/	64.7	V
**E) Sedentary-related equipment**				
How many of the following non-portable electronic devices do you use and are present at your home?				
Number of TVs	1.00 (1.00; 1.00)	/	100	V
Number of DVD players/video players	0.92 (0.82; 0.97)	/	95.0	V
Number of music players (radio, CD player, stereo,…)	0.86 (0.67; 0.94)	/	75.0	V
Number of computers (desktop)	0.80 (0.56; 0.92)	/	90.0	V
Number of telephones (dedicated line)	1.00 (1.00; 1.00)	/	100	V
How many of these non-portable electronic devices are present in your bedroom?				
Number of TVs	1.00 (1.00; 1.00)	/	100	V
Number of DVD players/video players	^	/	100	V
Number of music players (radio, CD player, stereo,…)	0.00 (-0.43; 0.43)	/	90.0	V
Number of computers (desktop)	^	/	100	V
Number of telephones (dedicated line)	0.89 (0.75; 0.96)	/	95.0	V
How many of the following portable electronic devices do you use and are present at your home?				
Number of laptops	1.00 (1.00; 1.00)	/	100	V
Number of mobile phones (no smartphone)	1.00 (1.00; 1.00)	/	100	V
Number of smartphones	1.00 (1.00; 1.00)	/	100	V
Number of music players (iPod, MP3,…)	0.67 (0.34; 0.86)	/	90.0	V
Number of tablets (iPad, Samsung Galaxy Tab,…)	0.67 (0.34; 0.86)	/	85.0	V
The remote controller (TV) can always be found closely to me when I need it	0.11 (-0.34; 0.52)	/	80.0	V
The couches at our place are comfortable to sit for a long time	-0.07 (-0.49;0.37)	/	85.0	V
**F) Sedentary behaviours**				
In the last 7 days, on average, how long did you spend sitting or lying down watching TV per weekday? (do include meals while sitting and watching TV)	0.82 (0.60; 0.93)	/	70.0	V
In the last 7 days, on average, how long did you spend sitting or lying down watching TV per weekend day? (do include meals while sitting and watching TV)	0.63 (0.27; 0.84)	/	40.0	V
In the last 7 days, on average, how long did you spend sitting/lying down during computer use per weekday?	0.83 (0.58; 0.94)	/	56.3	V
In the last 7 days, on average, how long did you spend sitting during computer use per weekend day?	0.83 (0.59; 0.94)	/	50.0	V
In the last 7 days, on average, how long did you sit while using motorized transport (e.g. to shops, friends,...) per weekday (in a car, bus, train, metro, on a motorbike, moped; do not include cycling on a pedal bicycle or electric bicycle)?	0.48 (0.06; 0.76)	/	25.0	V
In the last 7 days, on average, how long did you sit while using motorized transport (e.g. to shops, friends,...) per weekend day (in a car, bus, train, metro, on a motorbike, moped; do not include cycling on a pedal bicycle or electric bicycle)?	0.71 (0.39; 0.87)	/	25.0	V

How long of previous-mentioned time did you drive a motorized vehicle yourself per weekday (moped, motorbike or car; do not include cycling on an electric bicycle)?	0.58 (0.20; 0.81)	/	40.0	V
How long of previous-mentioned time did you drive a motorized vehicle yourself per weekend day (moped, motorbike or car; do not include cycling on an electric bicycle)?	0.83 (0.60; 0.93)	/	44.4	V
In the last 7 days, on average, how much time per day did you spend sitting while doing household tasks (e.g. cooking, ironing, small repairs like changing a lamp/battery) per weekday?	0.79 (0.54; 0.91)	/	65.0	V
In the last 7 days, on average, how much time per day did you spend sitting while doing household tasks (e.g. cooking, ironing, small repairs like changing a lamp/battery) per weekend day?	0.77 (0.50; 0.90)	/	75.0	V
In the last 7 days, on average, how much time per day did you spend sitting/lying down while making phone calls at home per weekday?	0.41 (-0.03; 0.72)	/	60.0	V
In the last 7 days, on average, how much time per day did you spend sitting/lying down while making phone calls at home per weekend day?	0.25 (-0.20; 0.62)	/	55.0	X
In the last 7 days, on average, how long did you spend sitting or lying down during the following activities per day?				
Sitting while reading (book, newspaper,…) on a weekday	0.92 (0.81; 0.97)	/	45.0	V
Sitting while reading (book, newspaper,…) on a weekend day	0.68 (0.35; 0.86)	/	35.0	V
Sitting while caring (grandchildren, others) on a weekday	0.20 (-0.25; 0.58)	/	80.0	V
Sitting while caring (grandchildren, others) on a weekend day	^	/	95.0	V
Sitting for hobbies (voluntary work, playing cards, sewing, Sudoku, crossword,…) on a weekday	0.22 (-0.24; 0.60)	/	50.0	X
Sitting for hobbies (voluntary work, playing cards, sewing, Sudoku, crossword,…) on a weekend day	0.19 (-0.27; 0.57)	/	50.0	X
Sitting for socializing (visiting friends, in a pub,…) on a weekday	0.54 (0.14; 0.79)	/	40.0	V
Sitting for socializing (visiting friends, in a pub,…) on a weekend day	0.49 (0.07; 0.76)	/	10.0	V
Sitting while listening to music (radio, CD,…) on a weekday	-0.10 (-0.51;0.35)	/	50.0	X
Sitting while listening to music (radio, CD,…) on a weekend day	-0.13 (-0.53;0.33)	/	50.0	X
Sitting during meals (breakfast,...) on a weekday - do NOT include meals while sitting and watching TV	0.82 (0.60; 0.92)	/	80.0	V
Sitting during meals (breakfast,...) on a weekend day - do NOT include meals while sitting and watching TV	0.83 (0.61; 0.93)	/	80.0	V
Afternoon nap on a weekday	0.95 (0.88; 0.98)	/	70.0	V
Afternoon nap on a weekend day	0.80 (0.56; 0.92)	/	75.0	V
**G) Simultaneous behaviour**				
How often do you do the following situations at the same time (simultaneously)?				
Watching TV AND using mobile phone	0.95 (0.89; 0.98)	/	85.0	V
Watching TV AND using computer/tablet	0.92 (0.80; 0.97)	/	85.0	V
Using computer AND using mobile phone	0.85 (0.67; 0.94)	/	90.0	V
Using computer AND listening to music	0.88 (0.72; 0.95)	/	65.0	V
Using mobile phone AND having conversation with friends/family	0.99 (0.98; 1.00)	/	95.0	V
Using mobile phone AND listening to music	0.94 (0.86; 0.98)	/	85.0	V
Sewing (or similar tasks) AND watching TV	0.78 (0.53; 0.91)	/	95.0	V

Note: ^ (scale has zero variance items), Δ (*At least one variable in test-retest analyses is a constant). Agree (% agreement), LPA (light physical activities), CI (confidence interval). Interpretation 'usability': V (item has moderate-to-excellent reliability, so can be used as a reliable item); X (item showed to have poor reliability, so cannot be used as a reliable single item)*
